# Supplementary material for: Transmembrane protein 135 regulates lipid homeostasis through its role in peroxisomal DHA metabolism
Source: Commun Biol. 2023 Jan 4;6:8. doi: 10.1038/s42003-022-04404-7 (PMC9813353; doi:10.1038/s42003-022-04404-7)
Supplement: Supplementary file 2 — Supplementary Information [file 42003_2022_4404_MOESM2_ESM.pdf]

Supplemental Information For

Transmembrane Protein 135 Regulates Lipid Homeostasis through its Role in Peroxisomal DHA Metabolism

Michael Landowski[1,2], Vijesh J. Bhute[1,3], Samuel Grindel[1], Zachary Haugstad[1], Yeboah K. Gyening[4,5], Madison Tytanic[4], Richard S. Brush[4], Lucas J. Moyer[1], David W. Nelson[6], Christopher R. Davis[6], Chi-Liang Eric Yen[6], Sakae Ikeda[1,2], Martin-Paul Agbaga[4,5], and Akihiro Ikeda[1,2*]

*Correspondence to: aikeda@wisc.edu

This PDF file includes:

Supplementary Figures 1-12
Supplementary Tables 1-12

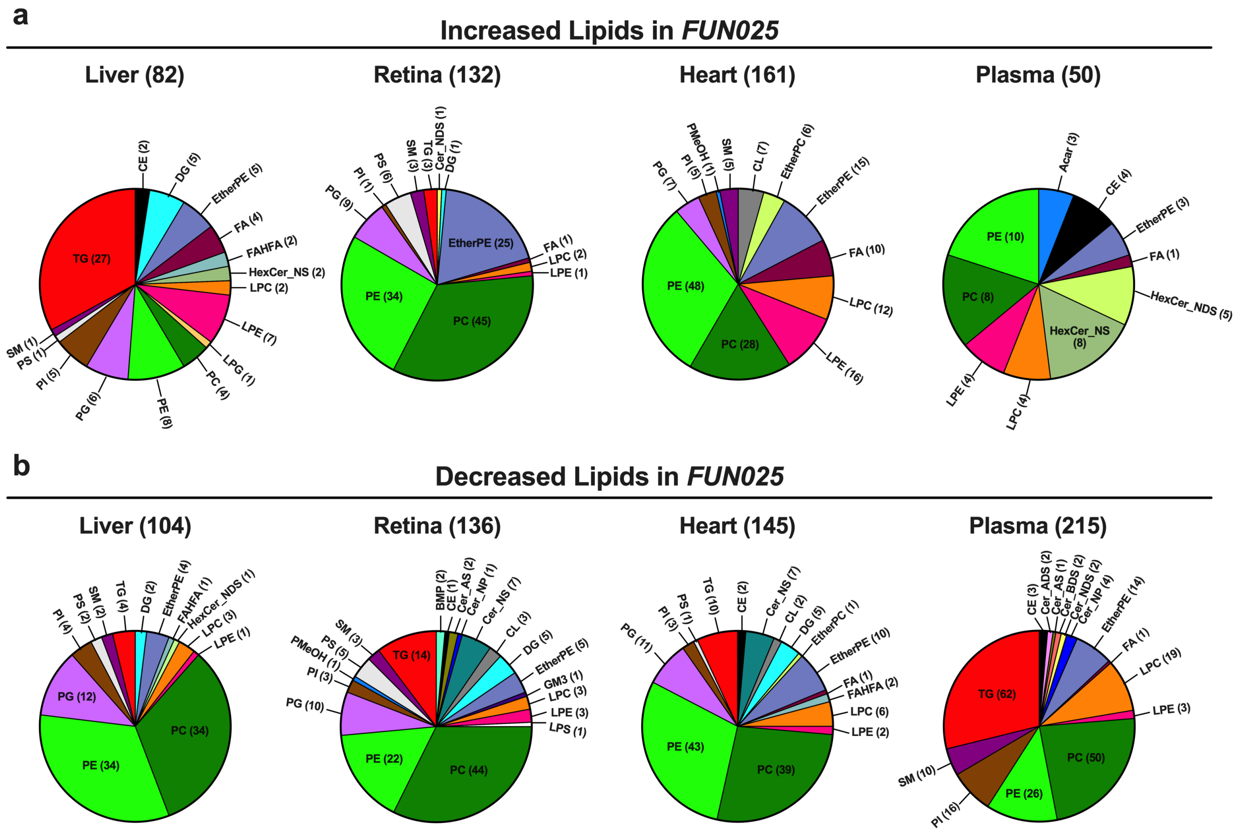

Supplementary Figure 1: Lipid class changes in *Tmem135* mutant tissues. Pie graphs of significantly (**a**) increased and (**b**) decreased lipids according to class in male *Tmem135*$^{FUN025/FUN025}$ (*FUN025*) livers, retinas, hearts, and plasmas compared to male WT controls. Numbers in brackets that are in bold font denote total number of lipids changed that reached statistical significance. Numbers in brackets next to lipid class denote total number of lipids changed within that class that reached statistical significance. Significance was determined by one-way ANOVA with post-hoc Tukey's HSD test between WT and *Tmem135*$^{FUN025/FUN025}$ livers (P<0.05). All altered lipid species can be found in **Supplementary Tables 6-9**. ACar, Acylcarnitine; BMP, Bismonoacylglycerophosphate; CE, Cholesteryl ester; CerADS, Ceramide alpha-hydroxy fatty acid-dihydrosphingosine; CerAS, Ceramide alpha-hydroxy fatty acid-sphingosine; CerBDS, Ceramide beta-hydroxy fatty acid-dihydrosphingosine; CerNDS, Ceramide non-hydroxy fatty acid-dihydrosphingosine; CerNP, Ceramide non-hydroxy fatty acid-phytosphingosine; CerNS, Ceramide non-hydroxy fatty acid-sphingosine; CL, Cardiolipin; DG, Diacylglycerol; EtherPC, Ether-linked phosphatidylcholine; EtherPE, Ether-linked phosphatidylethanolamine; FA, Free fatty acid; FAHFA, Fatty acid ester of hydroxyl fatty acid; GM3, Ganglioside; HexCerNDS, Hexosylceramide non-hydroxy fatty acid dihydrosphingosine; HexCerNS, Hexosylceramide non-hydroxy fatty acid sphingosine; LPA, Lysophosphatidic acid; LPC, Lysophosphatidylcholine; LPE, Lysophosphatidylethanolamine; LPG, Lysophosphatidylglycerol; PC, Phosphatidylcholine; PE, Phosphatidylethanolamine; PG, Phosphatidylglycerol; PI, Phosphatidylinositol; PMeOH, Phosphatidylmethanol; PS, Phosphatidylserine; SM, Sphingomyelin; and TG, Triacylglycerol.

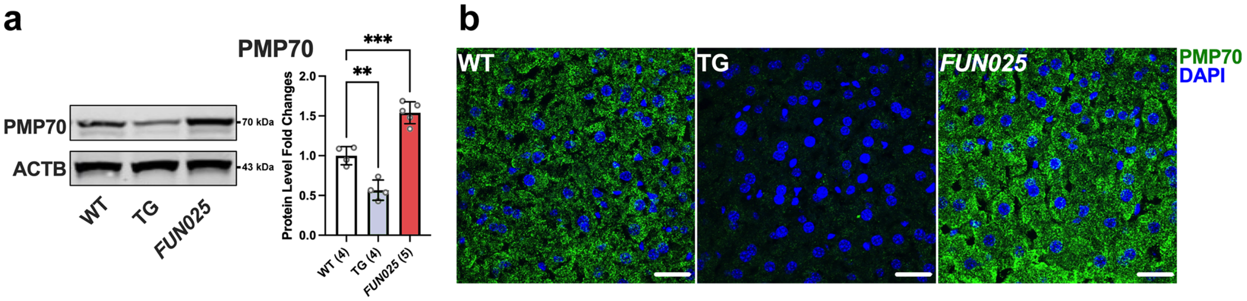

Supplementary Figure 2: Peroxisomal membrane protein 70 (PMP70) levels in livers of *Tmem135* mice. (**a**) Western blot analysis of PMP70 using livers from 2.5-month-old WT, *Tmem135* TG (TG), and *Tmem135*$^{FUN025/FUN025}$ (*FUN025*) mice. ACTB served as the loading control for this Western blot experiment. 4 WT (2 males/2 females), 4 *Tmem135* TG (2 males/2 females), and 5 *Tmem135*$^{FUN025/FUN025}$ (3 males/2 females) were used in this experiment. Asterisks ** and *** indicate post hoc Tukey test for a P<0.01 and P<0.001 significance following a significant difference detected by one-way ANOVA, respectively. The protein size next to the immunoblot images denote the size of the immunoband measured for this analysis. Dots represent individual data points. Data are presented as mean ± SD. (**b**) Representative 60X immunohistochemical images of PMP70 labeled (green) and DAPI stained (blue) WT, *Tmem135* TG, and *Tmem135*$^{FUN025/FUN025}$ livers. Scale bar for images = 30 microns.

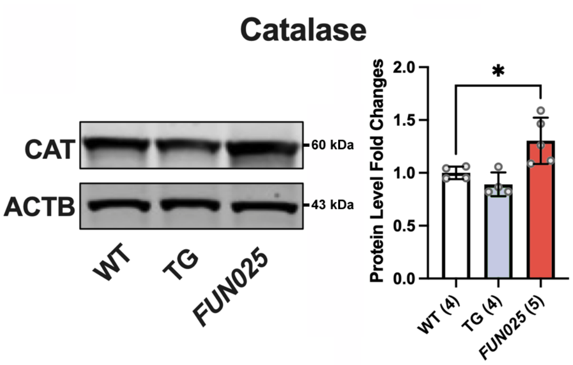

Supplementary Figure 3: Catalase levels in livers of *Tmem135* mice. Western blot analysis of catalase (CAT) using livers from 2.5-month-old WT, *Tmem135* TG (TG), and *Tmem135*$^{FUN025/FUN025}$ (*FUN025*) mice. ACTB served as the loading control for this western blot experiment. 4 WT (2 males/2 females), 4 *Tmem135* TG (2 males/2 females), and 5 *Tmem135*$^{FUN025/FUN025}$ (3 males/2 females) were used in this experiment. * indicates post hoc Tukey test for a P<0.05 significance following a significant difference detected by one-way ANOVA. The protein size next to the immunoblot images denotes the size of the immunoband measured for this analysis. Dots represent individual data points. Data are presented as mean ± SD.

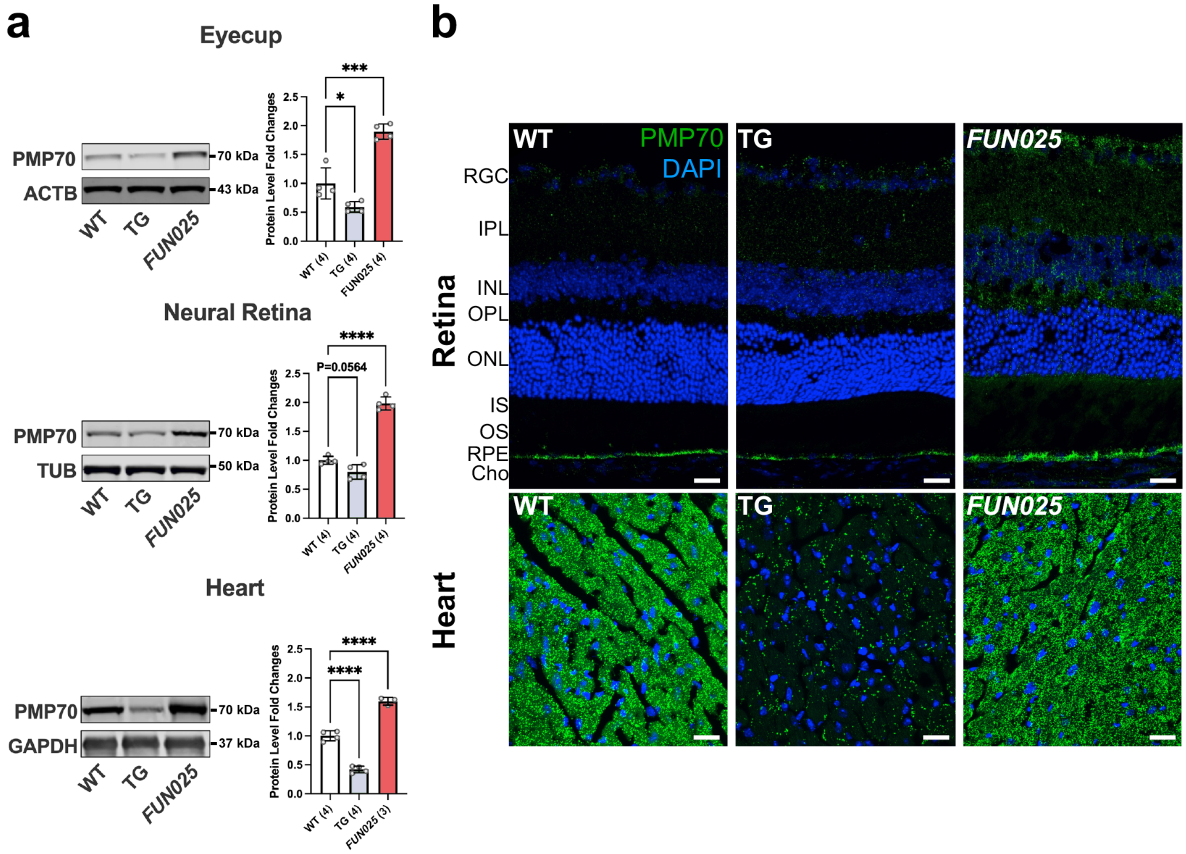

Supplementary Figure 4: Peroxisome membrane protein 70 (PMP70) levels in retinas and hearts of *Tmem135* mice. (**a**) Western blot analysis of PMP70 using eyecups, neural retinas, and heart from 2.5-month-old WT, *Tmem135* TG (TG), and *Tmem135*$^{FUN025/FUN025}$ (*FUN025*) mice. ACTB, TUB, and GAPDH served as the loading controls for these experiments. Asterisks (*, ***, and ****) indicates a P<0.05, P<0.001 and P<0.0001 significance by post hoc Tukey test following a significant difference detected by one-way ANOVA. Number in parentheses represent the N of independent mouse samples per genotype used in the experiment. The protein size next to the immunoblot images denotes the size of the immunoband measured for this analysis. Dots represent individual data points. Data are presented as mean ± SD. (**b**) Representative immunohistochemical images of PMP70 labeled (green) and DAPI stained (blue) WT, *Tmem135* TG, and *Tmem135*$^{FUN025/FUN025}$ retina and heart sections. For retina images, the magnification is 40X and scale bar is 20 microns. For heart images, the magnification is 60X and scale bar is 10 microns. RGC, retinal ganglion cells. IPL, inner plexiform layer. INL, inner nuclear layer. OPL, outer plexiform layer. ONL, outer nuclear layer. IS, inner segments. OS, outer segments. RPE, retinal pigmented epithelium. Cho, choroid.

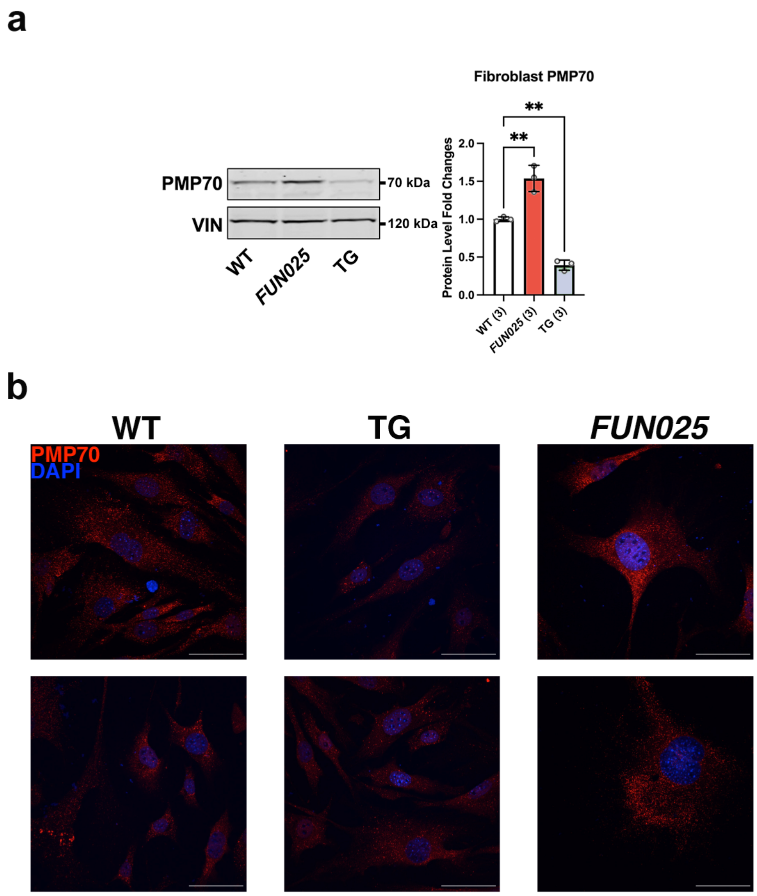

Supplementary Figure 5: Peroxisome levels in fibroblasts of *Tmem135* mice. (**a**) Western blot analysis of peroxisome membrane protein 70 (PMP70) using lysates derived from primary-cultured fibroblasts isolated from WT, *Tmem135* TG (TG), and *Tmem135*$^{FUN025/FUN025}$ (*FUN025*) mice. Vinculin (VIN) served as the loading control for this experiment. ** indicates a $P<0.01$ significance by post hoc Tukey test following a significant difference detected by one-way ANOVA. Number in parentheses represent the N of independent mouse samples per genotype used in the experiment. The protein size next to the immunoblot images denotes the size of the immunoband measured for this analysis. Dots represent individual data points. Data are presented as mean ± SD. (**b**) Representative immunohistochemical images of PMP70 labeled (red) and DAPI stained (blue) WT, *Tmem135* TG, and *Tmem135*$^{FUN025/FUN025}$ fibroblasts. Magnification is 60X and scale bar is 50 microns.

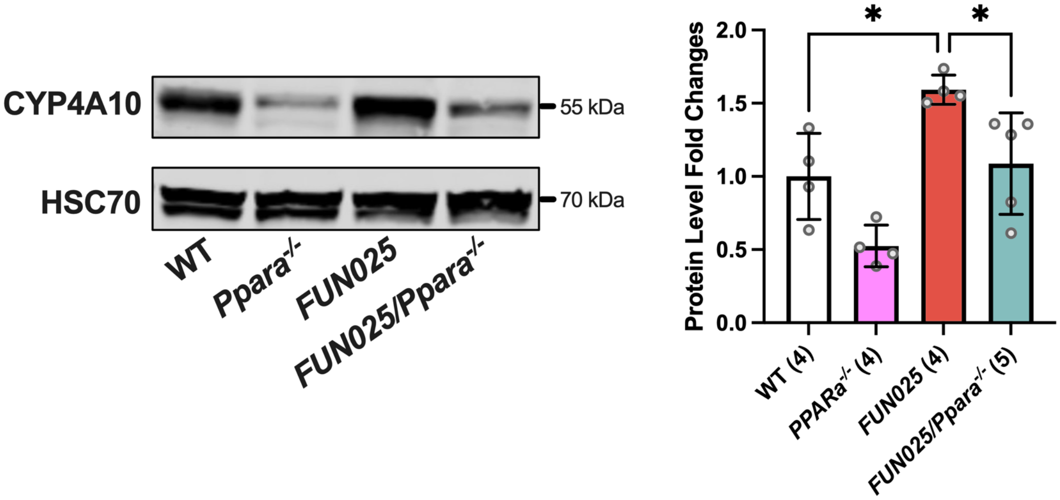

Supplementary Figure 6: Cytochrome P450, family 4, subfamily a, polypeptide 10 (CYP4A10) Protein Levels. Western blot analysis of CYP4A10 in the livers of 3-month-old WT (3 males/1 female), *Ppara*$^{-/-}$ (2 males /2 females), *Tmem135*$^{FUN025/FUN025}$ (2 males/2 females), and *Tmem135*$^{FUN025/FUN025}$/*Ppara*$^{-/-}$ (3 males/2 females) mice. HSC70 served as the loading control for this experiment. * indicates post hoc Tukey test for a $P<0.05$ significance following a significant difference detected by one-way ANOVA. Numbers in parentheses represent the number of samples used in the experiment. The protein size next to the immunoblot images denotes the size of the immunoband measured for this analysis. Dots represent individual data points. Data are presented as mean ± SD.

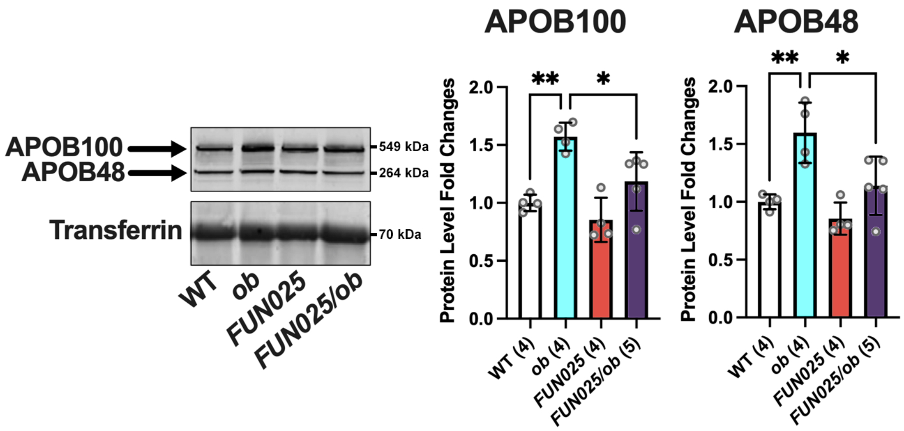

Supplementary Figure 7: Plasma apolipoprotein B in female mice. Western blot analysis of plasmas from 3-month-old WT, *Tmem135*$^{FUN025/FUN025}$ (*FUN025*), *Lep*$^{ob/ob}$ (*ob*), and *Tmem135*$^{FUN025/FUN025}$/*Lep*$^{ob/ob}$ (*FUN025/ob*) female mice for apolipoprotein B100 (APOB100) and B48 (APOB48). Transferrin served as a loading control for this experiment. Number in parentheses represent the N of independent mouse samples per genotype used in the experiment. The protein size next to the immunoblot images denotes the size of the immunoband measured for this analysis. Dots represent individual data points. Asterisks (* and **) indicates a $P<0.05$ and $P<0.01$ significance by post hoc Tukey test following a significant difference detected by one-way ANOVA. Data are presented as mean ± SD.

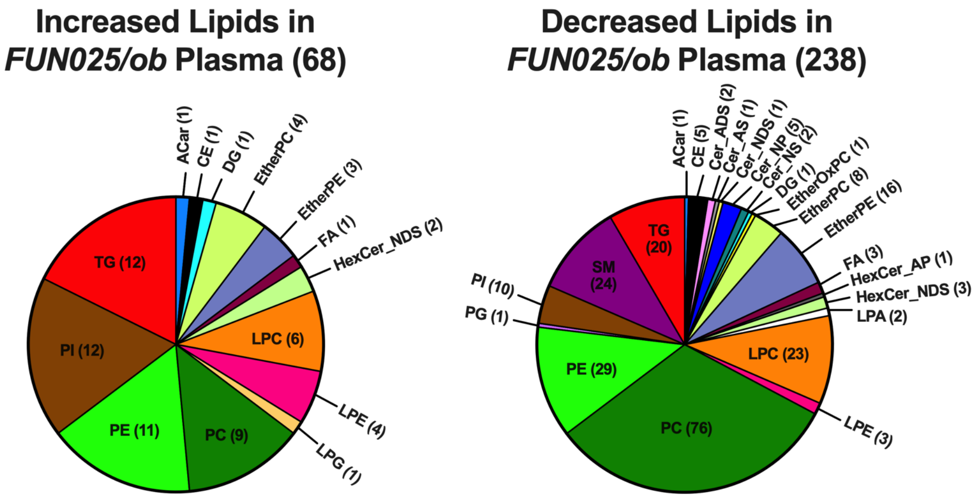

Supplementary Figure 8: Lipid class changes in *Tmem135* TG tissues. Pie graphs of significantly increased and decreased lipids according to class in male $Tmem135^{FUN025/FUN025}/Leptin^{ob/ob}$ (*FUN025/ob*) plasmas compared to male $Leptin^{ob/ob}$ controls. Numbers in brackets that are in bold font denote total number of lipids changed that reached statistical significance. Numbers in brackets next to lipid class denote total number of lipids changed within that class that reached statistical significance. Significance was determined by Student's T-Test ($P<0.05$). ACar, Acylcarnitine; CE, Cholesteryl ester; CerADS, Ceramide alpha-hydroxy fatty acid-dihydrosphingosine; CerAS, Ceramide alpha-hydroxy fatty acid-sphingosine; CerNDS, Ceramide non-hydroxy fatty acid-dihydrosphingosine; CerNP, Ceramide non-hydroxy fatty acid-phytosphingosine; CerNS, Ceramide non-hydroxy fatty acid-sphingosine; DG, Diacylglycerol; EtherOxPC, Ether-linked oxidized phosphatidylcholine; EtherPC, Ether-linked phosphatidylcholine; EtherPE, Ether-linked phosphatidylethanolamine; FA, Free fatty acid; HexCerAP, Hexosylceramide alpha-hydroxy fatty acid phytosphingosine; HexCerNDS, Hexosylceramide non-hydroxy fatty acid dihydrosphingosine; LPA, Lysophosphatidic acid; LPC, Lysophosphatidylcholine; LPE, Lysophosphatidylethanolamine; LPG, Lysophosphatidylglycerol; PC, Phosphatidylcholine; PE, Phosphatidylethanolamine; PG, Phosphatidylglycerol; PI, Phosphatidylinositol;
SM, Sphingomyelin; and TG, Triacylglycerol.

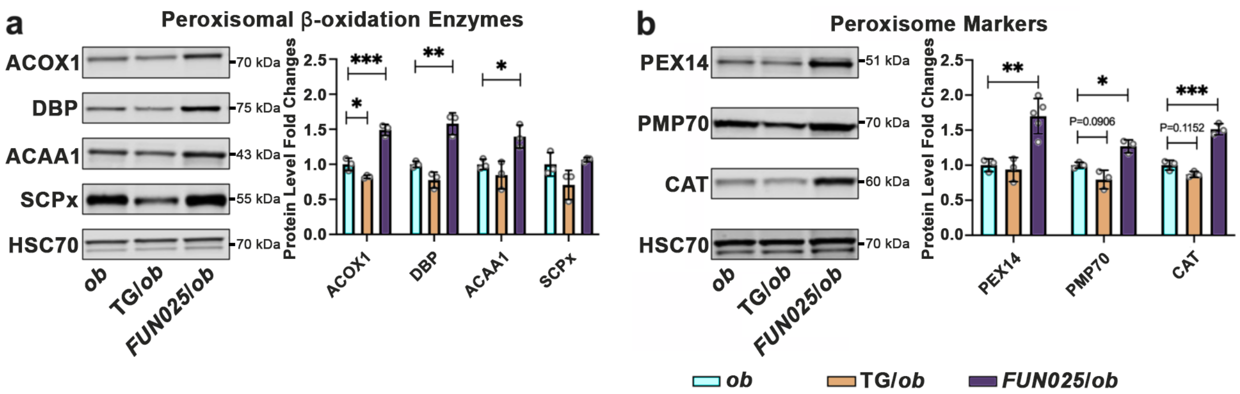

Supplementary Figure 9: Hepatic peroxisomal beta-oxidation enzyme levels and peroxisome markers in female mice. (**a**) Western blot analysis of peroxisomal beta-oxidation enzymes including Acyl-CoA Oxidase 1 (ACOX1), D-Bifunctional Protein (DBP), Acetyl-Coenzyme A Acyltransferase 1 (ACAA1), and Sterol Carrier Protein x (SCPx) using liver lysates from 3-month-old female $Lep^{ob/ob}$ (N=3), *Tmem135* TG/$Lep^{ob/ob}$ (TG/*ob*) (N=3) and $Tmem135^{FUN025/FUN025}$/$Lep^{ob/ob}$ (N=3) mice. (**d**) Western blot analysis of peroxisome biogenesis factor 14 (PEX14), peroxisome membrane protein 70 (PMP70), and catalase (CAT) using livers from 3-month-old female $Lep^{ob/ob}$ (N=3), *Tmem135* TG/$Lep^{ob/ob}$ (N=3) and $Tmem135^{FUN025/FUN025}$/$Lep^{ob/ob}$ (N=3) mice. For the PEX14 western, 5 female *Tmem135* TG/$Lep^{ob/ob}$ mice were used in this experiment. WT, *Tmem135* TG (TG), and $Tmem135^{FUN025/FUN025}$ mice. HSC70 served as the loading control for these experiments. Asterisks (*, **, and ***) indicates post hoc Tukey test for a P<0.05, P<0.01, and P<0.001 significance following a significant difference detected by one-way ANOVA. The protein size next to the immunoblot images denotes the size of the immunoband measured for this analysis. Dots represent individual data points. Data are presented as mean ± SD.

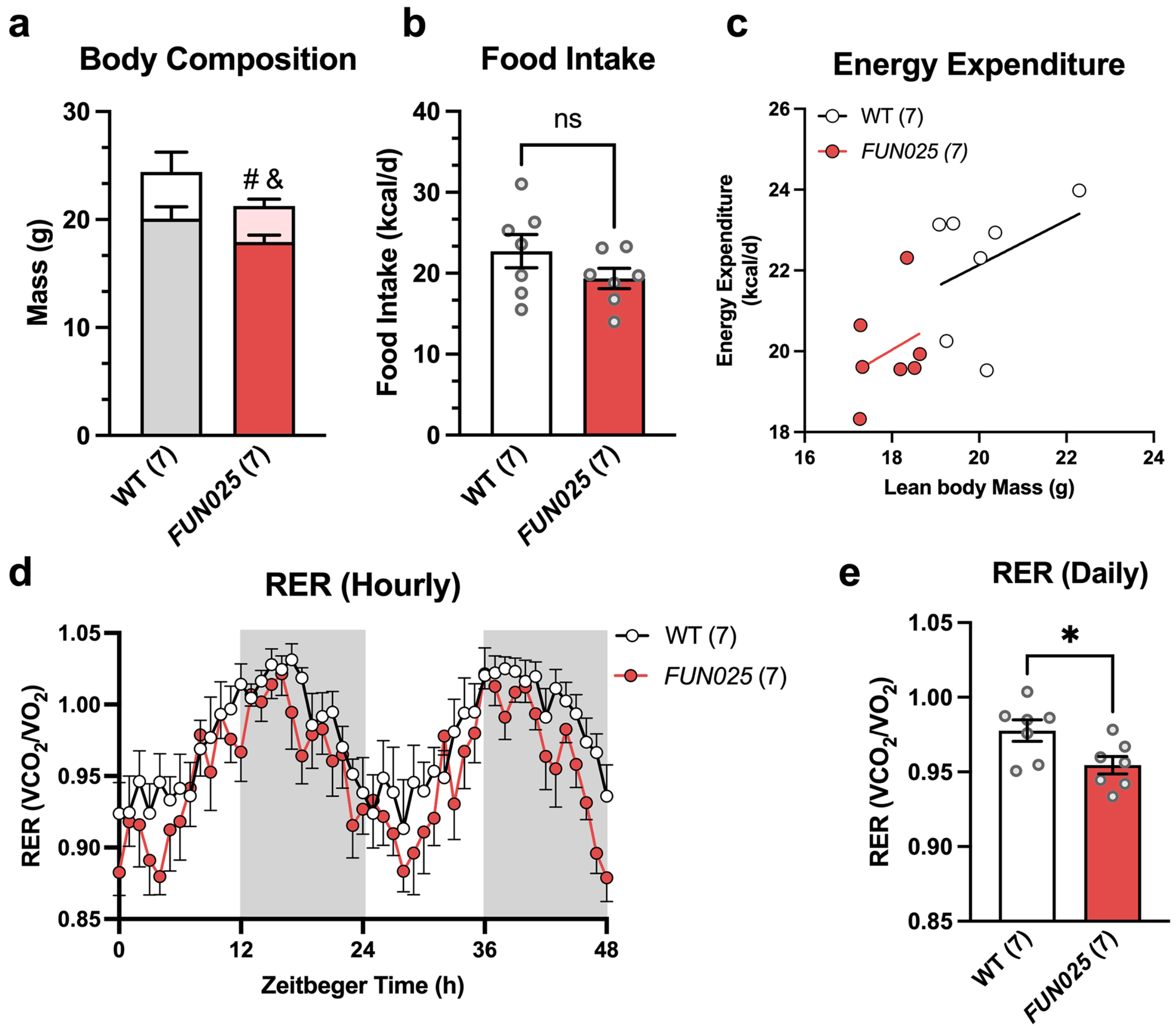

Supplementary Figure 10: *Tmem135* mutation increases fatty acid oxidation in chow fed mice. (**a**) Lean (dark bars) and fat (light bars) mass of 4-month-old male WT and *Tmem135*$^{FUN025/FUN025}$ (*FUN025*) mice measured by nuclear magnetic resonance (NMR) during 2 days of chow feeding. *Tmem135*$^{FUN025/FUN025}$ mice had significantly less total and lean mass after chow feeding than WT controls. Symbols (# and &) indicate a P<0.05 significance by Student's t-test between the lean and total mass of WT and *Tmem135*$^{FUN025/FUN025}$ mice, respectively. (**b**) Daily average of food intake per mouse during 2 days of chow feeding. Despite their smaller size, *Tmem135*$^{FUN025/FUN025}$ mice had similar intake of food intake compared to WT mice. (**c**) Linear regression of the daily average energy expenditure plotted against lean body mass of each WT and *Tmem135*$^{FUN025/FUN025}$ mouse. There were no significant differences in slopes or y-intercepts between WT and control *Tmem135*$^{FUN025/FUN025}$ mice (P=0.94 and 0.46, respectively). Thus, *Tmem135*$^{FUN025/FUN025}$ mice exhibited similar energy expenditure compared to WT control mice. (**d**) Hourly respiratory exchange ratio (RER) of WT and *Tmem135*$^{FUN025/FUN025}$ mice during 2 days of chow feeding. All mice preserved their circadian rhythm during metabolic phenotyping. Note the lower RER in the *Tmem135*$^{FUN025/FUN025}$ mice. Grey rectangles indicate the dark phase of the light cycle (7 P.M. to 7 A.M.). (**e**) Average daily RER of WT and *Tmem135*$^{FUN025/FUN025}$ mice during 2 days of chow feeding. *Tmem135*$^{FUN025/FUN025}$ mice had

lower average daily RER throughout the chow feeding experiment relative to WT controls. * indicates a $P<0.05$ significance by Student's t-test between the average daily RER of WT and *Tmem135*$^{FUN025/FUN025}$ mice. Together, our study found a significant decrease in the average daily RER but no changes in the food intake and energy expenditure between WT and *Tmem135*$^{FUN025/FUN025}$ mice. These results show a shift toward increased fatty oxidation in both the light and dark phases due to the *Tmem135* mutation in mice. Number in parentheses represent the N of independent mice per genotype used in the experiment. Dots represent individual data points. Data are presented as mean ± SD.

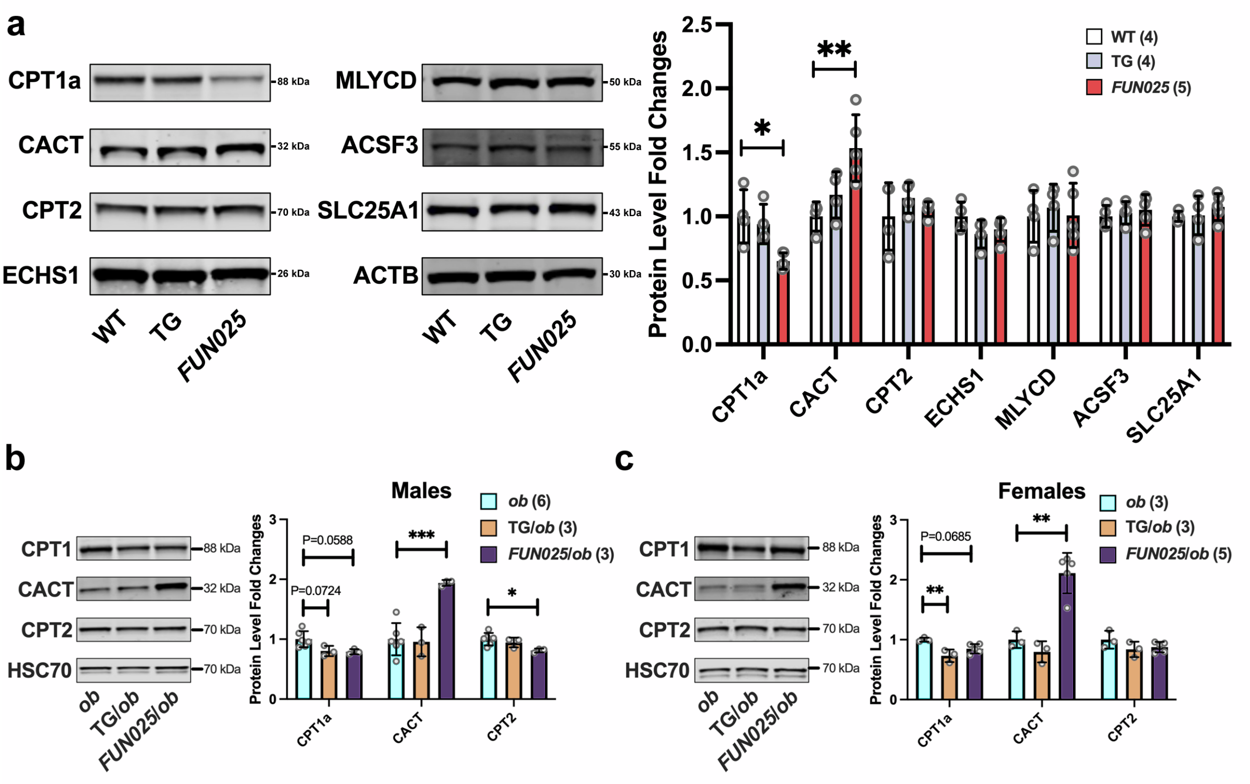

Supplementary Figure 11: Expression of mitochondrial proteins, CPT1a and CACT, is affected by the *Tmem135* mutation in mice. (**a**) Western blot analysis using 4 WT (2 males/2 females), 4 *Tmem135* TG (TG, 2 males/2 females), and 5 *Tmem135*$^{FUN025/FUN025}$ (*FUN025*, 3 males/2 females) mice was performed. ACTB served as the loading control for these experiments. CPT1A, carnitine palmitoyltransferase 1A. CACT, carnitine-acylcarnitine translocase. CPT2, carnitine palmitoyltransferase 2. ECHS1, enoyl-CoA hydratase, short chain 1. ACSF3, acyl-CoA synthetase family member 3. MLYCD, malonyl-CoA decarboxylase. SLC25A1, solute carrier family member 25 member 1. Western blot analysis of mitochondrial proteins CPT1a, CACT, and CPT2 in the livers of 3-month-old (**b**) male and (**c**) female *Lep*$^{ob/ob}$ (*ob*), *Tmem135* TG/*Lep*$^{ob/ob}$ (TG/*ob*) and *Tmem135*$^{FUN025/FUN025}$/*Lep*$^{ob/ob}$ (*FUN025/ob*) mice. HSC70 served as the loading control for these experiments. Number in parentheses represent the N of independent mouse samples per genotype used in the experiment. Asterisks (*, **, and ***) indicates a P<0.05, P<0.01, and P<0.001 significance by post hoc Tukey test following a significant difference detected by one-way ANOVA. The protein size next to the immunoblot images denotes the size of the immunoband measured for this analysis. Dots represent individual data points. Data are presented as mean ± SD.

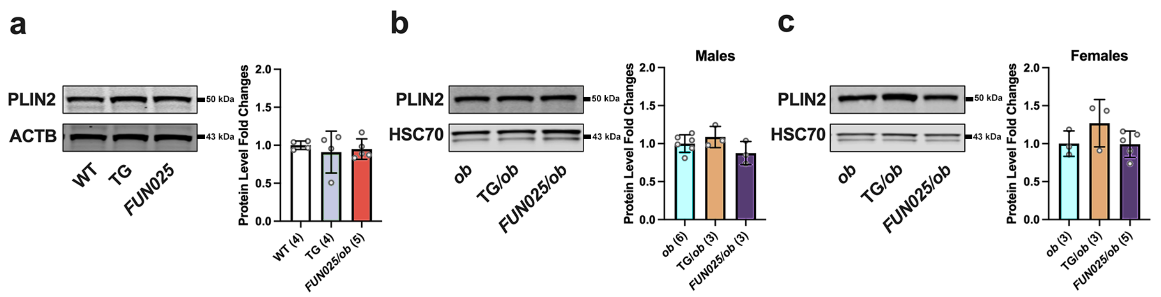

Supplementary Figure 12: Perilipin 2 (PLIN2) protein levels in *Tmem135* livers. (**a**) Western blot analysis of PLIN2 in 2.5-month-old liver lysates of WT, *Tmem135* TG (TG), and *Tmem135*$^{FUN025/FUN025}$ (*FUN025*) mice. ACTB served as the loading control for this experiment. 4 WT (2 males/2 females), 4 *Tmem135* TG (2 males/2 females), and 5 *Tmem135*$^{FUN025/FUN025}$ (3 males/2 females) were used in this experiment. (**b,c**) Western blot analysis of PLIN2 in the livers of 3-month-old male and female *Lep*$^{ob/ob}$ (*ob*), *Tmem135* TG/*Lep*$^{ob/ob}$ (TG/*ob*), and *Tmem135*$^{FUN025/FUN025}$/*Lep*$^{ob/ob}$ (*FUN025/ob*) mice. There were no significant changes in PLIN2 was observed in the experimental genotypes compared to their controls. Number in parentheses represent the N of independent mouse samples per genotype used in the experiment. The protein size next to the immunoblot images denote the size of the immunoband measured for this analysis. Dots represent individual data points. HSC70 served as the loading control for these experiments. Data are presented as mean ± SD.

Supplementary Table 1: Summary of Lipid Class Changes in *Tmem135*$^{FUN025/FUN025}$ (*FUN025*) Compared to WT.

Lipid Class	Liver			Retina			Heart			Plasmas		
	Total Detected	*FUN025*		Total Detected	*FUN025*		Total Detected	*FUN025*		Total Detected	*FUN025*	
		UP	DOWN		UP	DOWN		UP	DOWN		UP	DOWN
ACar							1	0	0	6	3	0
BMP				2	0	2						
CE	2	2	0	2	0	1	2	0	2	11	4	3
Cer_ADS				2	0	0				4	0	2
Cer_AP				1	0	0				5	0	0
Cer_AS	2	0	0	4	0	2				4	0	1
Cer_BDS				1	0	0				3	0	2
Cer_NDS	2	0	0	9	1	0				5	0	2
Cer_NP	7	0	0	6	0	1				8	0	4
Cer_NS	15	0	0	30	0	7	40	0	7	11	0	0
CL	12	0	0	12	0	3	18	7	2			
DG	17	5	2	14	1	5	9	0	5	1	0	0
EtherOxPC				1	0	0						
EtherPC	7	0	0	8	0	0	29	6	1	5	0	0
EtherPE	19	5	4	51	25	5	49	15	10	33	3	14
FA	9	4	0	11	1	0	31	10	1	23	1	1
FAHFA	7	2	1	5	0	0	8	0	2	4	0	0
GM3				6	0	1	4	0	0			
HBMP							6	0	0			
Hex_AP										1	0	0
HexCer_NDS	5	0	1	22	0	0				7	5	0
HexCer_NS	8	2	0	19	0	0	8	0	0	12	8	0
LPA										2	0	0
LPC	20	2	3	10	2	3	25	12	6	39	4	19

LPE	14	7	1	10	1	3	24	16	2	18	4	3
LPG	2	1	0				5	0	0	2	0	0
LPI	1	0	0	1	0	0	3	0	0			
LPS				1	0	1						
OxPl										1	0	0
PA				3	0	0						
PC	137	4	34	175	45	44	140	28	39	128	8	50
PE	99	8	34	111	34	22	141	48	43	67	10	26
PEtOH	7	0	0	1	0	0						
PG	32	6	12	32	9	10	45	7	11	3	0	0
PI	26	5	4	15	1	3	17	5	3	32	0	16
PMeOH	3	0	0	4	0	1	1	1	0			
PS	12	1	2	18	6	5	3	0	1			
SHexCer				1	0	0				1	0	0
SM	19	1	2	31	3	3	26	5	0	21	0	10
SQDG										1	0	0
TG	86	27	4	88	3	14	79	0	10	105	0	62
Total	570	82	104	707	132	136	714	161	145	563	50	215

Numbers denote number of lipid species within class that were detected. Grey indicates no detection of any lipid species within the lipid class in the tissue.

Supplementary Table 2: Summary of Acyl Side Chain Changes in *Tmem135*$^{FUN025/FUN025}$ (*FUN025*) Tissues Compared to WT.

Acyl Side Chain	Liver			Retina			Heart			Plasmas		
	Total Detected	*FUN025*		Total Detected	*FUN025*		Total Detected	*FUN025*		Total Detected	*FUN025*	
		UP	DOWN		UP	DOWN		UP	DOWN		UP	DOWN
C2				1	0	1				1	0	0
C12:0							3	0	0			
C13:0	1	0	0									
C14:0	12	2	2	13	6	0	14	2	2	7	1	0
C14:1	2	1	0	2	0	1				2	0	0
C14:2							1	0	0			
C15:0	9	0	3	12	2	1	9	3	1	12	0	6
C15:1				0	0	0	0	0	0	1	0	0
C16:0	93	26	9	119	20	20	85	21	8	95	7	30
C16:1	31	13	3	25	7	2	25	12	1	28	6	4
C16:2							3	2	0			
C16:3				2	1	0						
C17:0	16	0	6	9	0	1	18	4	3	19	0	7
C17:1	9	2	0	5	0	0	10	4	1	13	0	5
C17:2	1	0	0									
C18:0	68	7	14	96	16	17	72	18	15	66	6	22
C18:1	100	25	3	91	12	13	95	22	12	92	16	36
C18:2	91	20	10	59	21	7	133	58	13	89	6	39
C18:3	9	2	1	5	4	0	8	4	2	11	2	4
C18:4				1	0	1				1	0	0
C18:5										2	0	0
C19:0	14	0	2	7	1	1	29	7	6	17	0	11
C19:1	4	0	0	4	0	0	5	1	0	6	0	2
C19:2	2	0	0	1	0	0						

C20:0	21	1	4	18	0	5	26	6	5	19	3	6
C20:1	14	3	3	11	2	1	28	5	5	13	0	7
C20:2	7	0	0	9	1	0	12	1	2	8	1	1
C20:3	29	11	2	29	9	2	31	9	5	29	7	3
C20:4	77	0	23	61	6	9	73	15	19	69	2	43
C20:5	3	0	1	6	0	3	2	0	0	7	0	2
C21:0				1	0	0	8	2	0	4	1	0
C21:1	1	0	0							1	0	0
C21:2										1	0	0
C22:0	10	1	1	6	0	2	7	0	0	12	0	4
C22:1	2	0	0	4	0	0	6	0	0	8	0	2
C22:2				1	0	1	1	0	0	3	0	1
C22:3				5	2	0	5	2	0	1	0	0
C22:4	9	0	3	25	10	1	22	2	7	9	0	4
C22:5	23	1	15	31	8	14	54	2	36	25	0	14
C22:6	39	1	27	96	6	60	70	2	48	39	0	34
C22:7				1	0	1						
C23:0	3	0	0	3	0	1	3	0	0	6	0	3
C23:1	2	0	0	1	0	0						
C24:0	5	0	0	10	1	0	7	1	0	4	0	1
C24:1	4	0	1	6	0	1	5	0	0	5	1	0
C24:2				1	0	0	1	0	0	1	0	0
C24:4	1	0	0	8	4	1						
C25:0							1	0	0	1	0	0
C25:3	1	0	0									
C26:0				3	2	0	1	0	1	1	0	0
C26:1				2	2	0				1	0	0
C26:2				3	3	0				1	0	0

C26:4				6	3	2						
C27:0				1	0	0						
C28:2				1	0	0						
Ether Bond-Containing	26	5	4	60	26	4	78	21	11	38	3	14

Numbers denote number of lipid species that contain acyl side chain that were detected. Grey indicates no detection of any lipids containing acyl side chain within the tissue.

Supplementary Table 3: Relative Mole Percentages of Fatty Acids in WT and *Tmem135*$^{FUN025/FUN025}$ (*FUN025*) Tissues.

	Liver			Retina			Heart			Plasma		
Fatty Acid	WT (Avg ± StDev)	*FUN025* (Avg ± StDev)	Significance by T-test?	WT (Avg ± StDev)	*FUN025* (Avg ± StDev)	Significance by T-test?	WT (Avg ± StDev)	*FUN025* (Avg ± StDev)	Significance by T-test?	WT (Avg ± StDev)	*FUN025* (Avg ± StDev)	Significance by T-test?
14:0	0.272 ± 0.034	0.416 ± 0.079	*	ND	ND		ND	ND		ND	ND	
16:0	23.140 ± 1.250	24.288 ± 4.487	ns	27.119 ± 1.439	26.928 ± 0.374	ns	16.942 ± 0.898	15.477 ± 1.205	ns	24.021 ± 1.235	25.120 ± 3.851	ns
16:1	2.470 ± 0.197	4.313 ± 0.677	**	0.896 ± 0.154	2.870 ± 0.283	****	0.750 ± 0.098	2.361 ± 0.304	****	2.341 ± 0.401	4.417 ± 0.384	***
18:0	11.280 ± 0.804	10.791 ± 0.411	ns	21.999 ± 0.041	20.743 ± 0.384	***	18.715 ± 0.965	14.202 ± 0.879	***	9.609 ± 1.583	10.135 ± 1.920	ns
18:1	16.796 ± 3.088	27.069 ± 7.133	*	9.916 ± 0.525	14.538 ± 1.300	***	11.172 ± 1.165	17.304 ± 0.779	***	12.758 ± 1.281	22.092 ± 3.546	**
18:2n6	21.862 ± 2.458	18.062 ± 1.585	*	2.161 ± 0.372	5.076 ± 0.319	****	17.523 ± 1.660	34.785 ± 1.401	****	29.010 ± 2.484	27.788 ± 2.2023	ns
18:3n6	0.376 ± 0.072	0.428 ± 0.032	ns	ND	ND		ND	ND		0.434 ± 0.106	0.604 ± 0.064	*
18:3n3	0.537 ± 0.087	0.145 ± 0.025	***	ND	ND		ND	ND		0.186 ± 0.050	0.141 ± 0.046	ns
20:0	0.200 ± 0.092	0.123 ± 0.091	ns	0.685 ± 0.044	0.729 ± 0.031	ns	0.503 ± 0.038	0.315 ± 0.048	***	0.197 ± 0.073	0.149 ± 0.070	ns
20:1	0.479 ± 0.115	0.593 ± 0.059	ns	0.226 ± 0.022	0.497 ± 0.038	****	0.573 ± 0.130	0.447 ± 0.068	ns	0.510 ± 0.185	0.484 ± 0.074	ns
20:3n9	0.384 ± 0.041	0.224 ± 0.010	***	ND	ND		0.463 ± 0.009	0.195 ± 0.009	****	0.279 ± 0.030	0.222 ± 0.014	**
20:5n3	0.192 ± 0.036	0.119 ± 0.011	*	0.129 ± 0.011	0.059 ± 0.005	***	ND	ND		0.282 ± 0.060	0.110 ± 0.006	**
20:2n6	0.328 ± 0.026	0.484 ± 0.071	**	0.198 ± 0.035	0.281 ± 0.014	**	0.125 ± 0.017	0.238 ± 0.013	****	ND	ND	
20:3n6	1.126 ± 0.172	2.204 ± 0.536	**	0.622 ± 0.062	1.810 ± 0.242	****	0.828 ± 0.122	1.161 ± 0.140	*	1.0182 ± 0.099	2.013 ± 0.128	****
20:4n6	11.966 ± 0.316	8.580 ± 0.985	***	8.019 ± 0.512	11.095 ± 0.356	****	8.720 ± 0.757	11.496 ± 1.244	**	13.299 ± 2.147	4.928 ± 0.807	***
22:0	0.450 ± 0.127	0.328 ± 0.119	ns	0.618 ± 0.022	0.668 ± 0.036	ns	0.441 ± 0.015	0.381 ± 0.007	***	0.373 ± 0.079	0.297 ± 0.053	ns
22:1	0.432 ± 0.016	0.573 ± 0.054	**	0.684 ± 0.214	0.776 ± 0.330	ns	ND	ND		0.447 ± 0.035	0.583 ± 0.056	**
22:4n6	0.339 ± 0.039	0.179 ± 0.025	***	1.050 ± 0.041	2.786 ± 0.128	****	0.716 ± 0.046	0.453 ± 0.015	****	0.160 ±0.014	0.072 ± 0.005	****
22:5n6	0.465 ± 0.029	0.140 ± 0.007	****	0.327 ± 0.045	0.560 ± 0.063	**	2.646 ± 0.307	0.273 ± 0.013	****	0.341 ± 0.021	0.089 ± 0.017	****
22:5n3	0.334 ± 0.087	0.103 ± 0.020	**	0.453 ± 0.105	0.905 ± 0.079	***	0.917 ± 0.188	0.269 ± 0.015	***	0.233 ± 0.050	0.073 ± 0.009	***
22:6n3	5.938 ± 0.144	0.206 ± 0.018	****	24.781 ± 1.510	9.600 ± 0.780	****	18.642 ± 0.734	0.367 ± 0.026	****	3.884 ± 0.403	0.103 ± 0.014	****
24:0	0.301 ± 0.059	0.287 ± 0.066	ns	0.118 ± 0.064	0.078 ± 0.021	ns	0.162 ± 0.011	0.127 ± 0.010	**	0.186 ± 0.029	0.158 ± 0.007	ns
24:1	0.332 ± 0.044	0.343 ± 0.023	ns	ND	ND		0.161 ± 0.005	0.150 ± 0.005	ns	0.431 ± 0.079	0.421 ± 0.130	ns
n6/n3	5.207 ± 0.254	52.426 ± 2.892	****	0.489 ± 0.025	2.053 ± 0.127	****	1.564 ± 0.148	76.269 ± 3.994	****	9.676 ± 0.576	83.469 ± 5.292	****

ND, not detected. ns, not significant.

Supplementary Table 4: Summary of Lipid Class Changes in Male *Tmem135*/Leptin Mutant (FUN025/*ob*) Mice Compared to Leptin mutant (*ob*) Mice.

Lipid Class	Plasmas		
	Total Detected	*FUN025*	
		UP	DOWN
ACar	8	1	1
CE	11	1	5
Cer_ADS	5	0	2
Cer_AP	3	0	0
Cer_AS	4	0	1
Cer_BDS	1	0	0
Cer_NDS	5	0	1
Cer_NP	9	0	5
Cer_NS	11	0	2
DG	4	1	1
EtherOxPC	2	0	1
EtherPC	18	4	8
EtherPE	32	3	16
FA	13	1	3
FAHFA	5	0	0
HexCer_AP	2	0	1
HexCer_NDS	14	2	3
HexCer_NS	2	0	0
LPA	2	0	2
LPC	49	6	23
LPE	15	4	3
LPG	1	1	0
LPI	2	0	0

**OxPl**	**1**	**0**	**0**
**PC**	**118**	**9**	**76**
**PE**	**61**	**11**	**29**
**PG**	**4**	**0**	**1**
**PI**	**30**	**12**	**10**
**SM**	**30**	**0**	**24**
**TG**	**82**	**12**	**20**
**Total**	**544**	**68**	**238**

Numbers denote number of lipid species within class.

Supplementary Table 5: Summary of Acyl Side Chain Changes in Male *Tmem135*/Leptin Mutant (FUN025/*ob*) Mice Compared to Leptin mutant (*ob*) Mice.

Acyl Side Chain	Plasmas		
	Total Detected	*FUN025*	
		UP	DOWN
C8	1	0	1
C12:0	1	0	0
C14:0	8	1	4
C14:1	1	1	0
C14:3	1	0	1
C15:0	10	0	6
C15:1	1	0	1
C16:0	84	19	25
C16:1	25	8	6
C16:2	1	0	1
C17:0	14	0	6
C17:1	12	0	3
C18:0	69	15	27
C18:1	91	21	20
C18:2	83	11	22
C18:3	9	3	3
C19:0	7	1	1
C19:1	3	0	1
C20:0	10	2	4
C20:1	18	3	8
C20:2	10	3	4
C20:3	42	10	9
C20:4	61	0	49

**C20:5**	**7**	**0**	**6**
**C21:0**	**1**	**0**	**1**
**C22:0**	**8**	**0**	**2**
**C22:1**	**4**	**0**	**2**
**C22:3**	**2**	**1**	**0**
**C22:4**	**9**	**0**	**4**
**C22:5**	**0**	**0**	**16**
**C22:6**	**37**	**0**	**36**
**C23:0**	**3**	**0**	**2**
**C24:0**	**5**	**0**	**0**
**C24:1**	**6**	**0**	**3**
**C26:0**	**2**	**0**	**2**
**C26:1**	**1**	**0**	**0**
**C26:2**	**2**	**0**	**1**
**C28:2**	**1**	**0**	**1**
**Ether Bond-Containing**	**52**	**7**	**25**

Numbers denote number of lipid species within class.

Supplementary Table 6: Significantly-Altered Lipids in *Tmem135*$^{FUN025/FUN025}$ (*FUN025*) Livers Compared to WT.

Lipid Species	Normalized Log10 Lipid Concentrations								Contains DHA
	WT 1	WT 2	WT 3	WT 4	*FUN025* 1	*FUN025* 2	*FUN025* 3	*FUN025* 4	
CE 18:1	-0.2407	0.1055	0.1021	-0.4466	0.7132	0.3345	0.2407	0.5859	
CE 18:2	-0.0272	0.2904	-0.5617	-0.5082	0.8953	0.1164	0.3098	0.6858	
DG 16:0_16:1	0.0222	-0.0135	0.0702	-0.1075	0.8663	0.0860	0.5268	0.6037	
DG 16:0_18:1	0.0425	0.0668	0.0232	-0.3717	0.9682	0.1188	0.5393	0.5878	
DG 16:0_18:2	-0.0017	0.1713	0.1410	-0.5616	0.8609	0.0824	0.5503	0.6757	
DG 16:0_22:6	0.4891	0.6989	0.4422	0.4029	-0.5650	-1.2858	-0.9452	-1.1554	x
DG 16:1_18:2	-0.0992	0.2970	0.2457	-0.4073	0.8923	0.1313	0.7027	0.7108	
DG 18:1_18:1	-0.0303	0.0250	0.0563	-0.5381	0.9906	0.2510	0.7135	0.5608	
DG 18:2_22:5	0.2023	0.1780	0.0374	-0.1834	-0.4908	-0.9905	-0.8352	-0.5556	
EtherPE 14:1e_20:4	0.4048	0.4720	0.3482	0.3199	-1.1381	-1.2914	-1.3109	-0.2445	
EtherPE 16:1e_18:1	-0.3638	-0.0297	-0.1647	-0.2263	0.9649	0.6966	0.6345	0.7135	
EtherPE 16:1e_18:2	-0.3354	-0.2458	-0.3805	-0.3699	1.0656	0.7850	0.7463	0.8436	
EtherPE 16:1e_20:1	-0.2250	0.2141	-0.1160	-0.0945	0.8541	0.3651	0.5037	0.4879	
EtherPE 16:1e_20:3	-0.6912	0.1538	-0.1795	-0.1190	1.1196	0.8697	0.8039	0.9076	
EtherPE 18:1e_18:2	-0.1671	0.0716	-0.2073	-0.1156	0.7936	0.6422	0.4910	0.2169	
EtherPE 18:1e_20:4	0.1840	0.5510	0.1877	0.1660	-0.2206	-0.6248	-0.7139	-0.4197	
EtherPE 18:1e_22:4	0.4232	0.8026	0.0849	0.2171	-0.0107	-0.3621	-0.3461	-0.1018	
EtherPE 18:2e_22:5	0.1830	-0.4352	-0.1624	-0.1644	-0.9400	-1.1484	-0.5884	-0.6977	
FA 16:0_1	-1.0056	-0.9591	-0.3182	-0.7360	0.9491	0.5652	0.6078	0.7688	
FA 16:1	-0.2117	0.1541	0.1517	-0.2295	0.9945	0.4291	0.7446	0.8315	
FA 18:1	-0.1495	0.0961	0.0043	-0.3672	0.8588	0.2750	0.3957	0.6288	
FA 18:2	-0.1893	0.2406	0.0281	-0.5438	0.8087	0.1787	0.2878	0.6240	
FAHFA 16:1_18:3	0.0279	0.3346	0.4585	0.1437	1.0535	0.7337	1.0105	1.0017	
FAHFA 18:1_20:3	-0.1440	0.0738	-0.0060	-0.3880	0.9164	0.2981	0.3983	0.6695	
FAHFA 22:4_18:2	0.2901	0.7415	0.0654	0.0963	0.0031	-0.6261	-0.5720	-0.4025	

HexCer_NDS d40:1	-0.2111	0.2050	-0.2522	-0.3593	0.8040	0.3762	0.1763	0.3361	
HexCer_NS d18:1_20:0	-0.1811	-0.5131	-0.0458	-0.3490	0.9173	0.3666	0.3592	0.4432	
HexCer_NS d18:1_22:0	-0.2134	0.2029	-0.2544	-0.3654	0.8020	0.3741	0.1863	0.3340	
LPC 0:0/20:4	0.5281	0.6244	0.2442	0.6641	0.0984	-0.4541	-0.5018	-0.3086	
LPC 0:0/22:6	0.6644	0.7069	0.4911	0.7456	-0.8091	-0.9799	-1.8442	-0.8888	x
LPC 16:0/0:0	0.0216	0.0684	-0.2517	-0.0697	0.9183	0.2387	0.1168	0.4696	
LPC 18:2/0:0	0.0173	0.0673	-0.2325	-0.1936	0.9631	0.3793	0.1544	0.6156	
LPC 22:5/0:0	0.6971	0.7217	0.4723	0.8823	-0.8263	-0.9960	-1.0176	0.2135	
LPE 16:0	-0.3360	-0.2840	-0.3658	-0.2721	1.0510	0.3802	0.4930	0.8179	
LPE 16:0_1	-0.3337	-0.2818	-0.3634	-0.2698	1.0587	0.3653	0.4901	0.8198	
LPE 16:1	-0.3512	-0.0579	-0.0647	-0.0342	1.0057	0.4251	0.6068	0.8939	
LPE 16:1_1	-0.3512	-0.0579	-0.0647	-0.0342	1.0057	0.4251	0.6068	0.8939	
LPE 18:0	-0.4117	-0.1344	-0.3038	-0.3337	1.0305	0.2652	0.2745	0.7127	
LPE 18:0_1	0.3145	0.4660	0.3726	0.3739	-1.4530	-1.6386	0.7042	-1.2791	
LPE 18:1	-0.2270	-0.3527	-0.2670	-0.2234	1.0246	0.4104	0.5208	0.8036	
LPE 20:1	-0.5201	-0.1817	-0.4804	-0.2457	0.7420	0.1368	0.0793	0.6023	
LPG 16:0	-0.0931	-0.1717	-0.1857	-0.3450	0.9267	0.1970	0.2098	0.7668	
PC 14:0_20:4	-0.0288	0.4998	0.1888	0.1042	0.1236	-0.5604	-0.7537	-0.6220	
PC 15:0_18:2	0.1112	0.6354	-0.0414	0.1129	-0.2653	-0.8099	-0.5973	-0.4480	
PC 16:0_17:1	-0.3912	-0.3550	-0.4820	-0.5112	0.6667	0.8866	1.0544	1.2360	
PC 16:0_20:3	-0.3752	0.0133	-0.4573	-0.3684	0.8400	0.2188	0.3118	0.5120	
PC 16:0_20:4	0.3556	0.7436	0.2445	0.2631	-0.3762	-0.8558	-0.9446	-0.6499	
PC 16:0_20:5	-0.0126	0.6939	0.5769	0.1108	-0.9298	-1.0842	-1.1039	-0.9731	
PC 16:0_22:6	0.6009	0.8306	0.4930	0.4623	-1.1345	-1.3072	-1.3640	-1.2022	x
PC 16:1_20:4	0.4130	0.7428	0.3675	0.1460	0.0524	-0.3774	-0.6834	-0.1259	
PC 16:1_22:6	0.6953	0.8612	0.6911	0.4532	-0.9338	-0.8257	-1.5748	-1.1213	x
PC 17:0_18:2	0.4119	0.8658	0.6674	0.4100	-1.0769	-1.2374	0.4342	-1.1220	
PC 17:0_20:4	0.6004	-0.1809	0.4139	0.2424	-0.5381	-0.9823	-0.4137	-0.8398	

PC 18:0_20:3	-0.2677	0.6822	0.5910	-0.0034	-1.5488	-0.5487	-1.2622	-0.2223	
PC 18:0_20:4	0.3515	0.8483	0.1849	0.2921	-0.1465	-0.7119	-0.8432	-0.5417	
PC 18:0_22:5	0.5841	0.4510	0.5847	0.0123	-1.0730	-1.2367	-0.2571	-1.1189	
PC 18:0_22:6	-0.2956	-0.5400	-1.0138	-0.2190	0.6718	0.6855	0.6500	0.8277	x
PC 18:1_20:4	0.4646	0.7244	0.3531	0.2877	-0.4528	-0.7914	-0.9017	-0.6734	
PC 18:2_22:5	0.4277	0.4865	0.3020	0.1115	-0.2826	-0.6505	-0.7864	-0.3459	
PC 20:0_22:6	0.6113	0.6681	0.3385	-0.1459	-0.8139	-1.0285	-1.8785	-0.6586	x
PC 20:1_22:6	0.5026	0.5893	0.2439	0.2629	-0.4915	-0.8010	-0.8446	-0.8379	x
PC 31:0	-0.1315	-0.4135	-0.4571	-0.3803	0.9280	0.3221	0.4607	0.7386	
PC 34:0	0.1789	0.6690	0.2276	0.1888	-0.1209	-0.6229	-0.7660	-0.1602	
PC 36:5	0.2991	0.5181	0.0215	-0.1926	-0.3899	-0.7523	-0.6711	-0.3927	
PC 36:6	0.5843	0.8439	0.5263	0.4332	-1.4268	-1.5482	-1.5637	-0.7189	
PC 37:5	0.5415	0.9049	0.4509	0.0818	-0.6119	-0.6567	-1.3200	-0.8121	
PC 37:6	0.5665	0.6104	0.3870	0.2631	-0.8938	-1.0872	-1.6942	-0.0551	
PC 38:5	-0.1039	0.0470	-0.4001	-0.4112	-0.8360	-0.8291	-0.6887	-0.3845	
PC 39:4	0.3106	0.5349	0.1373	0.2560	-0.7418	-0.7819	-0.9180	-0.6853	
PC 39:6	0.5832	0.6859	0.3354	0.3336	-0.3251	-1.6624	-0.7006	-0.7091	
PC 39:7	0.6608	0.7209	0.4801	0.4967	-0.4789	-1.7548	-1.0001	-1.6641	
PC 40:4	0.4903	0.5055	0.1101	0.3352	-0.4370	-0.7590	-1.2639	-0.5004	
PC 40:5	0.3412	0.7016	0.1595	0.2779	-0.3040	-0.7433	-0.8029	-0.4699	
PC 40:5_1	-0.1642	0.2254	-0.2509	-0.0952	-0.4819	-0.7648	-0.8545	-0.8774	
PC 40:6	0.5364	0.7701	0.3618	0.4705	-1.0152	-1.1729	-1.1014	-1.2536	
PC 40:6_1	-0.0824	-0.0962	-0.3116	-0.4016	-0.6600	-0.9138	-0.6592	-0.6354	
PC 40:6_2	0.3890	0.4060	0.9604	-0.0213	-1.1174	-1.4055	-0.3734	-1.3829	
PC 40:7	0.5534	0.7728	0.4836	0.4382	-0.9865	-1.4264	-1.1662	-1.0733	
PC 40:8	0.5202	0.6816	0.3155	0.3335	-0.6818	-1.1237	-1.0259	-0.8438	
PC 42:10	0.5359	0.7184	0.4569	0.3351	-0.8522	-1.0200	-1.0414	-0.8993	
PE 14:0_22:6	0.4792	0.5948	0.3518	0.6013	-0.6252	-1.6137	-1.6314	-0.4742	x

PE 15:0_20:4	0.3656	0.3598	0.0226	0.0785	-0.1181	-0.5236	-1.3370	-0.2391	
PE 15:0_22:6	0.5914	0.6880	0.3637	0.5442	-0.7208	-1.4244	-1.6552	-0.9118	x
PE 16:0_18:1	-0.0938	-0.2218	-0.3116	0.0191	0.8380	0.2721	0.3143	0.5351	
PE 16:0_18:2	-0.3637	-0.3070	-0.5247	-0.3642	0.9130	0.3558	0.5681	0.8934	
PE 16:0_20:3	-0.2122	0.0429	-0.2698	-0.3682	0.7463	0.2972	0.3173	0.5606	
PE 16:0_22:5	-0.2038	0.0177	-0.2327	-0.0236	-0.6105	-0.8764	-0.9206	-0.6329	
PE 16:0_22:6	0.4652	0.6260	0.3950	0.4217	-0.8809	-1.2338	-1.1579	-0.9437	x
PE 16:1_18:2	-0.1752	-0.0598	-0.3039	-0.1481	0.8569	0.3085	0.4469	0.7907	
PE 16:1_22:6	0.4818	0.7563	0.5149	0.5441	-1.2086	-0.9282	-0.7987	-1.1698	x
PE 17:0_20:4	0.3490	0.3361	-0.0135	0.1860	-0.3257	-0.8609	-0.7470	-0.2802	
PE 17:0_20:4_1	0.2461	0.4975	0.1752	0.1895	-1.0176	-0.2733	-0.7955	-0.5597	
PE 17:0_22:6	0.4714	0.3396	0.3102	0.3331	-0.6603	-0.4152	-1.7840	-1.2247	x
PE 17:1_18:2	-0.0823	-0.0527	-0.3891	-0.1475	0.7304	0.0719	0.1651	0.7258	
PE 17:1_22:6	0.7985	0.6133	0.4790	0.4782	-1.1903	-1.1227	-0.6846	-0.4342	x
PE 18:0_18:2	-0.1643	-0.0916	-0.5899	-0.3914	0.7708	0.1272	0.2556	0.6983	
PE 18:0_20:1	0.0123	0.3096	-0.1685	-0.0532	-0.1592	-0.6147	-0.7854	-0.3181	
PE 18:0_20:3_1	-0.5930	-0.3706	-0.4621	-0.2653	0.7415	0.0875	0.1929	0.1329	
PE 18:0_20:4_2	0.0376	0.5934	0.1471	0.1369	-0.3243	-0.5539	-0.7726	-0.2512	
PE 18:0_22:4_1	0.2446	-0.1540	0.0997	-0.0177	-0.3349	-0.6957	-0.6908	-0.2729	
PE 18:0_22:5	-0.0032	0.2192	-0.0351	-0.0266	-0.7546	-1.1576	-1.1033	-0.9709	
PE 18:0_22:5_1	-0.0745	0.4349	0.7227	-0.3539	-1.0459	-1.1786	-1.1955	-1.0831	
PE 18:0_22:6	0.5122	0.6661	0.3560	0.4354	-1.0166	-1.2531	-1.1792	-1.0233	x
PE 18:1_18:2	-0.1479	-0.6503	-0.1830	-0.4942	0.7795	0.2366	0.3316	0.8870	
PE 18:1_22:6	0.4714	0.6296	0.4056	0.3929	-0.8665	-1.1669	-1.1829	-0.9021	x
PE 18:2_22:6	0.3412	0.4955	0.2346	0.1966	-0.5156	-0.9987	-0.9504	-0.6191	x
PE 19:0_20:4	0.3728	0.3345	-0.0635	0.0759	-0.6136	-0.7790	-0.9603	-0.5212	
PE 19:0_20:4_1	0.4251	0.3706	0.1905	0.2078	-0.7658	-1.3992	-0.9673	-0.6458	
PE 20:0_20:4	0.3054	0.1054	-0.1142	-0.0318	-0.3195	-0.7179	-0.7732	-0.1265	

PE 20:0_20:4_1	0.3580	0.5121	0.1910	0.1649	-0.1213	-0.4726	-1.0728	-0.3074	
PE 20:0_22:6	0.6501	0.6296	0.2559	0.5178	-0.4575	-1.4076	-1.4246	-1.3114	x
PE 20:1_22:6	0.2610	-0.2813	0.4263	0.5035	-0.8199	-1.9687	-0.2974	-0.6487	x
PE 20:4_22:6	0.5782	0.5723	0.2301	0.4105	-0.7051	-1.4972	-0.8707	-0.7288	x
PE 24:1_20:4	0.4451	0.6473	0.1176	0.5966	-0.0590	-0.1226	-1.1990	-1.9850	
PE 37:6	0.4449	0.5096	0.0206	0.3155	-0.3796	-0.9790	-0.7898	-0.4590	
PE 38:5	0.4703	0.6636	0.4854	0.6855	-0.1332	-1.1886	-2.0886	-0.1389	
PE 38:7	0.5642	0.8237	0.1944	0.3198	-0.7034	-0.3808	-0.4237	-0.5095	
PE 39:7	0.4780	0.6795	0.4488	0.4169	-0.3439	-0.7546	-1.7044	-0.5706	
PE 40:6	0.3497	0.8301	0.1693	0.2981	-0.2232	-0.7438	-0.8586	-0.5571	
PE 40:7	0.4461	0.7169	0.4252	0.3647	-0.4117	-0.8312	-0.9205	-0.6972	
PE 40:7_1	0.4954	0.7787	0.3828	0.3467	-0.8225	-1.1255	-1.1561	-0.9799	
PE 42:7	0.2954	0.6133	0.3580	0.2374	-0.0115	-0.6626	-0.6345	-0.4685	
PG 16:0_18:2	0.3145	0.5771	0.1753	0.2229	-0.2529	-0.6317	-0.7663	-0.1840	
PG 16:1_18:1_1	-0.1759	-0.0472	-0.0023	-0.1406	1.0424	0.5814	0.6590	0.8451	
PG 16:1_18:2	-0.2515	0.0877	0.0428	-0.1072	0.9380	0.5755	0.6992	0.8541	
PG 18:1_18:1	-0.2222	0.0909	0.0064	-0.1624	0.9439	0.4913	0.5293	0.6361	
PG 18:1_18:2	-0.3620	-0.0134	-0.0475	-0.2058	0.9631	0.5632	0.5906	0.8030	
PG 18:1_20:3	-0.2853	0.1636	0.1519	-0.0770	0.7248	0.7631	0.6045	0.7126	
PG 18:1_22:5	-0.0636	0.1441	0.0632	-0.0061	-0.4684	-1.4424	-0.7852	-0.4955	
PG 18:2_18:2_1	-0.3034	0.2078	0.0223	-0.2613	0.8221	0.4027	0.4495	0.7607	
PG 18:2_20:4	0.4394	0.7683	0.5691	0.1409	0.1017	-0.0942	-0.2285	-0.1202	
PG 18:2_22:5	0.3978	0.5875	0.4354	0.3337	-0.9391	-1.0054	-0.9716	-0.8504	
PG 18:2_22:6	0.5639	0.6988	0.5211	0.3450	-0.8969	-0.9971	-1.0672	-0.8495	x
PG 18:3_22:6	0.9119	1.0645	0.0832	0.2716	-0.9422	-1.0874	-1.1059	-0.9829	x
PG 20:3_22:5	0.3974	0.5505	-0.1850	0.4020	-0.9784	-1.1256	-1.1444	-1.0197	
PG 20:4_22:5	0.1223	0.1720	-0.0257	0.1358	-0.8724	-1.0295	-1.0495	-0.9165	
PG 20:4_22:6	0.6634	0.7107	0.4614	0.3304	-0.8104	-0.9843	-1.0064	-0.8592	x

PG 22:5_22:5	-0.0730	-0.0270	0.2116	0.2369	-1.3587	-1.4777	-0.7656	-1.3921	
PG 22:5_22:6	0.1004	0.1199	-0.0375	-0.0965	-0.8844	-1.0354	-1.0547	-0.9267	x
PG 22:6_22:6	0.6419	0.6517	0.3944	0.1451	-0.8294	-1.0005	-1.0223	-0.8774	x
PI 16:0_20:3	-0.5933	-0.2083	-0.0788	-0.3914	0.7436	0.3065	0.8149	0.8531	
PI 16:0_20:3_1	-0.4609	-0.3101	-0.2058	-0.4433	0.7365	0.1705	0.6882	0.5980	
PI 16:0_22:6	0.4025	0.3814	0.6460	0.5333	-0.7224	-1.1140	-1.1168	-0.8957	x
PI 17:0_20:4	0.5072	0.3597	0.2456	0.4249	-0.5137	-0.9468	-0.1790	-0.1336	
PI 18:0_20:3	-0.4073	-0.2955	-0.0491	-0.6002	0.7204	0.2720	0.7830	0.9505	
PI 18:0_20:3_1	-0.7757	-0.4316	-0.0771	-0.4112	0.6615	0.2097	0.7516	0.7169	
PI 18:0_22:5	-0.0112	-0.1592	-0.2274	-0.1915	-0.6514	-1.1255	-0.8140	-0.5039	
PI 18:0_22:6	0.4812	0.5420	0.6214	0.2274	-0.8729	-1.2572	-0.8711	-0.7343	x
PI 18:1_20:3	-0.0006	-0.4828	-0.2719	-0.2231	0.5683	0.2657	0.3957	0.7430	
PS 16:0_22:6	0.5781	0.5734	0.6591	0.2888	-0.8884	-0.6916	-1.3232	-1.4629	x
PS 18:0_22:6	0.4024	0.5217	0.5147	0.2108	-0.8257	-0.8729	-0.9139	-0.9000	x
PS 18:2_18:3	-0.0936	0.0423	-0.3051	-0.2308	0.9574	0.2031	0.2175	0.7492	
SM d18:0_22:0	0.3118	0.6385	0.2077	0.0837	-0.0441	-0.5498	-0.4821	-0.4173	
SM d41:2	0.1890	0.2772	-0.0193	0.1331	0.1016	-0.9525	-0.6439	-0.4255	
SM d41:3	0.7741	-0.6515	0.0701	-0.7766	1.2960	0.5717	1.0494	1.1882	
TG 14:0_16:0_18:1	-0.3577	-0.1605	-0.0861	-0.3440	1.3074	0.0968	0.6340	0.9033	
TG 14:0_16:1_18:2	-0.6707	-0.3106	0.1439	-0.2060	1.2646	0.8265	0.6056	1.0642	
TG 14:1_14:1_18:1	-0.7336	0.0790	-0.7375	-1.6102	0.5685	1.1082	1.1750	1.2717	
TG 16:0_16:0_16:0	-0.2002	-0.3940	-0.3545	-0.0156	0.9890	0.5064	0.2096	0.9470	
TG 16:0_16:0_18:1	-0.4399	-0.1409	0.0577	-0.4408	1.3412	0.4132	0.7412	0.8993	
TG 16:0_16:1_18:1	-0.4692	-0.0559	0.1617	-0.2785	1.3744	0.3359	0.7492	0.8959	
TG 16:0_16:1_18:2	-0.3393	0.1054	0.2531	-0.3221	1.2094	0.3051	0.6512	0.8194	
TG 16:0_18:0_18:1	-0.3099	0.1848	-0.3952	-0.1472	1.1832	0.1593	0.4189	0.6227	
TG 16:0_18:1_18:1	-0.5314	-0.0355	0.2138	-0.4737	1.3139	0.4491	0.7676	0.8684	
TG 16:0_18:1_18:2	-0.5293	0.1144	0.2325	-0.5266	1.3290	0.2815	0.6520	0.8511	

TG 16:0_18:1_20:1	-0.4993	0.0715	0.1644	-0.5340	1.2498	0.1338	0.3456	0.6332	
TG 16:0_18:1_22:5	-0.9377	-1.2616	-0.4431	-0.2766	0.4446	0.0067	-0.4523	0.4821	
TG 16:0_18:2_18:2	-0.4975	0.3247	0.3011	-0.5134	1.1161	0.1773	0.4819	0.7614	
TG 16:1_16:1_18:2	-0.8556	0.3145	0.2625	-0.2175	1.0804	0.3784	0.6404	0.8516	
TG 48:2	-0.5754	-0.3180	-0.0453	-0.8044	1.1821	0.2483	0.6517	0.8697	
TG 48:2_1	-0.4731	-0.2366	0.0139	-0.5668	1.1415	0.4165	0.6100	0.8545	
TG 48:3	-0.6665	-0.3041	0.1591	-0.1989	1.2808	0.7511	0.6170	1.0539	
TG 50:1	-0.4543	-0.1535	0.0474	-0.4551	1.3432	0.4057	0.7364	0.8950	
TG 50:2	-0.4693	-0.0560	0.1616	-0.2786	1.3744	0.3359	0.7494	0.8958	
TG 50:3	-0.4392	0.1053	0.2531	-0.3225	1.2102	0.3052	0.6515	0.8198	
TG 50:4	-0.8342	0.2861	0.2711	-0.1946	1.0796	0.3857	0.6447	0.8534	
TG 52:1	-0.5464	0.2241	-0.3314	-0.0937	1.1804	0.1997	0.4484	0.6403	
TG 52:2	-0.5318	-0.0357	0.2137	-0.4741	1.3142	0.4491	0.7664	0.8686	
TG 52:3	-0.5293	0.1144	0.2325	-0.5266	1.3290	0.2815	0.6520	0.8511	
TG 52:4	-0.4986	0.3242	0.3005	-0.5145	1.1161	0.1767	0.4800	0.7612	
TG 54:4	-0.5409	0.1540	0.3072	-0.6524	1.2152	0.3383	0.6382	0.8390	
TG 56:4	-0.2747	-0.0271	0.1300	-0.7015	1.0434	0.2944	0.4684	0.6690	
TG 56:8	0.2785	0.6724	0.8572	0.4328	-1.6305	-1.1695	-0.8600	-0.4990	
TG 58:10	0.5725	0.7184	0.3768	0.1703	-0.5739	-0.2138	-1.9085	-0.7648	
TG 58:8	-1.1756	0.5441	0.6753	0.4467	-1.4156	-0.2330	-1.2576	-1.1926	
TG 58:9	0.1294	0.4146	0.3222	-0.0330	-0.3425	-0.8879	-1.3568	-0.6779	

Red denotes upregulation, blue denotes downregulation.

Supplementary Table 7: Significantly-Altered Lipids in *Tmem135*$^{FUN025/FUN025}$ (*FUN025*) Retinas Compared to WT.

Lipid Species	Normalized Log10 Lipid Concentrations								Contains DHA
	WT 1	WT 2	WT 3	WT 4	*FUN025* 1	*FUN025* 2	*FUN025* 3	*FUN025* 4	
BMP 22:5_22:6	0.2876	0.2769	0.3964	0.3320	-0.5968	-1.0183	-0.1523	-1.9294	x
BMP 22:6_22:6	0.3649	0.3052	0.4336	0.3237	-0.7597	-0.9087	-0.9058	-0.8890	x
CE 22:6	0.3906	0.0216	0.5295	0.3516	-0.8096	-0.7755	-0.9582	-1.8064	x
Cer_AS d18:1_16:0	0.3665	0.3304	-0.0938	0.2750	-0.3596	-0.0703	-0.7652	-0.7592	
Cer_AS d18:1_16:0_1	0.3649	0.3289	-0.0951	0.2681	-0.3609	-0.0717	-0.7661	-0.7602	
Cer_NDS d18:0_24:0	-0.4681	-0.5319	-0.5503	-0.5258	0.9750	1.1662	0.4818	0.6056	
Cer_NP t18:0_20:0	0.5235	0.4109	0.4131	0.4572	-1.4230	-1.4870	0.6306	-2.3382	
Cer_NS d18:1_16:0	0.1033	-0.0677	0.2009	0.0375	-0.0515	-0.3984	-0.4919	-0.3154	
Cer_NS d18:1_22:2	0.1144	-0.2408	0.5188	0.1867	-0.1446	-0.4831	-0.6933	-0.3039	
Cer_NS d18:1_23:0	0.1600	-0.0934	0.1631	0.0735	-0.0615	-0.4534	-0.4018	-1.0032	
Cer_NS d18:2_16:0	0.2804	0.0682	-0.0519	0.2471	-0.3895	-0.4621	-0.6047	-0.3516	
Cer_NS d18:2_18:0	0.2148	-0.0943	0.4146	-0.1116	-0.1507	-0.3707	-0.5273	-0.4997	
Cer_NS d18:2_22:0	0.1725	-0.2431	0.2432	-0.0098	-0.0914	-0.6352	-0.4075	-0.4790	
Cer_NS d18:2_24:1	0.1383	-0.2094	0.2551	0.0961	-0.1778	-0.5137	-0.5485	-0.4739	
CL 18:0_18:2_18:1_18:1	0.1963	-0.0053	0.3992	-0.3328	-0.2981	-0.4183	-0.5629	-0.3110	
CL 68:2	0.2363	0.1382	0.5639	-0.2568	-0.5175	-0.5346	-0.4227	-0.4092	
CL 70:3	-0.0098	0.2240	0.4763	-0.1145	-0.3115	-0.5929	-0.6578	-0.2087	
DG 14:0_16:0	-0.0977	-0.0441	-0.4831	-0.9435	0.7688	0.4386	0.5539	0.4758	
DG 16:0_20:4	0.0885	-0.0444	0.1021	0.0903	-0.2370	-0.6859	-0.4274	-0.4441	
DG 16:0_22:6	0.4736	0.4037	0.4220	0.4680	-0.9780	-0.9326	-1.0818	-1.0736	x
DG 18:0_20:5	0.1809	0.1873	0.2597	0.4391	-1.2146	-0.4136	-0.1651	-1.0126	
DG 18:0_22:6	0.4396	0.2445	0.3482	0.3521	-0.9648	-1.1091	-0.9802	-0.6076	x
DG 22:6_22:6	0.3445	0.0599	0.3343	0.1673	-0.4777	-0.6306	-0.6529	-0.8163	x
EtherPE 14:1e_20:4	0.3546	0.3170	0.0337	0.2192	-0.1469	-0.6962	-0.5621	-0.5725	
EtherPE 16:0e_16:0	-0.3612	-0.3115	0.1294	0.1297	1.0782	0.7224	0.6941	0.7948	

EtherPE 16:0e_18:2	-0.2347	-0.2991	-0.2897	-0.2084	1.0662	0.6349	0.7569	0.6905	
EtherPE 16:0e_22:4	-0.2738	-0.4719	-0.3286	-0.3739	1.2369	0.9125	1.0204	0.9463	
EtherPE 16:1e_16:0	-0.2407	-0.2207	-0.1891	-0.4431	0.7901	0.3454	0.2851	0.3449	
EtherPE 16:1e_16:1	0.1225	-0.1859	-0.2193	-1.3173	0.9429	0.8157	0.7977	0.7692	
EtherPE 16:1e_16:3	-0.7609	0.1505	0.0175	-0.8081	1.1832	1.0634	0.9684	1.0284	
EtherPE 16:1e_18:1	-0.0791	-0.3541	-0.2301	-0.2068	0.8524	0.4393	0.6358	0.3909	
EtherPE 16:1e_18:2	0.1711	0.2987	-1.4778	-1.4623	1.3202	1.1775	1.1865	1.1875	
EtherPE 16:1e_18:3	-0.0674	-0.1581	-1.2677	-0.0539	1.1153	0.9510	0.8811	1.1442	
EtherPE 16:1e_20:3	0.0181	-0.0693	0.0349	-0.3161	1.0597	0.8604	0.9146	0.9286	
EtherPE 16:1e_20:3_1	0.2212	-0.5136	0.2267	-0.7001	0.9184	0.9014	0.9359	0.8561	
EtherPE 16:1e_22:4	-0.3844	-0.4553	-0.2568	-0.2936	0.8945	0.6250	0.6346	0.6486	
EtherPE 16:1e_22:5	-0.4628	-0.5933	-0.4360	-0.5837	0.4617	0.0588	0.1115	0.1303	
EtherPE 16:1e_22:6	0.2823	0.1859	0.2737	0.1631	-0.1757	-0.6980	-0.5683	-0.6111	x
EtherPE 18:0e_22:4	-0.4035	-0.4412	-0.3113	-0.3991	1.0222	0.6496	0.7802	0.7678	
EtherPE 18:0e_22:5	0.0192	0.0624	0.3223	-0.0016	1.1887	1.0609	1.0543	1.1253	
EtherPE 18:1e_16:0	-0.7941	-0.2114	-0.1162	-0.1237	0.8590	0.5856	0.6162	0.5285	
EtherPE 18:1e_18:2	-0.1563	-0.3929	-0.2826	-0.2374	0.9182	0.6552	0.6852	0.6505	
EtherPE 18:1e_20:3	0.2777	-0.3430	-0.1734	0.2215	1.1798	0.2620	0.9564	0.9497	
EtherPE 18:1e_22:3	-0.1604	-0.9591	-0.4966	-0.4316	0.8215	0.5417	0.5231	0.7259	
EtherPE 18:1e_22:4	-0.1057	-0.4869	-0.3358	-0.3679	0.6906	0.3504	0.3636	0.3223	
EtherPE 18:1e_22:5	-0.4373	-0.7937	-0.4824	-0.5211	-0.0042	-0.3039	-0.2278	-0.2951	
EtherPE 18:1e_22:5_1	-0.0007	-0.2818	-0.2096	-0.1266	0.7910	0.4576	0.4291	0.4486	
EtherPE 18:1e_22:6	0.3393	0.2627	0.3072	0.2530	-0.6005	-0.8877	-0.8457	-0.8436	x
EtherPE 18:1e_24:4	-0.1724	-0.9620	-0.8251	-0.1303	0.6959	0.6828	0.7400	0.6113	
EtherPE 18:2e_22:6	0.3180	0.2659	0.2727	0.2730	-0.4174	-0.7242	-0.7633	-0.7802	x
EtherPE 18:3e_18:2	-0.2700	-0.5800	0.0844	-0.1158	1.0771	0.7278	0.7865	0.8949	
EtherPE 38:5e	0.0001	0.0337	-1.1432	-1.0331	0.7909	0.5804	0.5489	0.6093	
EtherPE 40:5e	0.1667	-1.0789	-1.0910	-1.0749	1.3251	1.3337	1.2717	1.3603	

FA 20:0	0.2678	-0.0155	0.0407	0.3818	-0.2993	-0.4919	-0.4113	-0.6693	
GM3 d36:1	0.1792	-0.0782	0.4517	0.0179	-0.1096	-0.2689	-1.0672	-0.3313	
LPC 0:0/16:0	0.2576	-0.2728	0.4501	-0.0529	-0.0387	-0.5670	-0.4052	-0.6003	
LPC 0:0/16:1	-0.4792	-0.1204	0.1106	-0.2212	0.9482	0.5816	0.7372	0.6043	
LPC 0:0/18:2	-0.1552	-0.6180	-0.0338	-0.2027	0.7652	0.1303	0.3417	0.2593	
LPC 0:0/22:5	-0.0349	-0.3825	-0.0585	-0.1909	-0.4138	-0.8726	-0.6774	-0.9009	
LPC 0:0/22:6	0.5021	0.2908	0.5848	0.4068	-0.9152	-1.1970	-1.1029	-1.1688	x
LPE 18:1	-0.2423	-0.4740	0.0440	-0.2653	0.7055	0.0262	0.4386	0.4531	
LPE 22:6	0.2702	0.2793	0.5438	0.2060	-0.4284	-1.4835	-0.9995	-0.7369	x
LPE 22:6_1	0.2746	0.2837	0.4947	0.2102	-0.4258	-1.4832	-0.9981	-0.7348	x
LPS 22:6	0.4273	0.1870	1.0111	-0.0623	-0.4516	-1.6882	-0.6733	-0.4450	x
PC 14:0_14:0	-0.2595	-0.3208	-0.1743	-0.2610	0.7573	0.3581	0.3618	0.4404	
PC 14:0_16:1	-1.1476	-0.2179	-0.3951	-0.2109	1.1242	0.9747	0.9390	0.9279	
PC 14:0_18:2	-0.3015	-0.0348	-0.2351	-0.5735	0.3893	0.2486	1.0592	0.9570	
PC 15:0_16:0	-0.1427	-0.5783	0.1582	-0.1080	0.6694	0.2197	0.2147	0.3522	
PC 15:0_18:0	-0.3177	-0.6908	0.0692	-0.2354	0.7073	0.4925	0.2613	0.4259	
PC 16:0_16:0	0.1324	0.0045	0.0654	0.0444	-0.1132	-0.4838	-0.5401	-0.4529	
PC 16:0_16:1	-0.2953	-0.4994	-0.0339	-0.3025	0.9749	0.7402	0.6988	0.7265	
PC 16:0_16:3	-0.1783	-0.4274	-0.0484	-0.5343	0.7051	0.1988	0.2572	0.4892	
PC 16:0_17:0	0.5385	0.5127	0.3297	0.3717	-0.4899	-0.2402	-0.7237	-1.0654	
PC 16:0_18:0	0.1014	-0.0653	0.2017	0.0626	-0.2313	-0.6114	-0.6184	-0.5786	
PC 16:0_18:2	-0.3160	-0.5576	-0.0818	-0.3225	0.7868	0.5471	0.5395	0.5986	
PC 16:0_18:3	-0.1679	-0.8705	-0.1574	-0.2724	0.6825	0.2981	0.4371	0.4390	
PC 16:0_20:3	-0.1525	-0.5797	0.1722	-0.2394	0.6611	0.2307	0.2293	0.2810	
PC 16:0_22:4_1	-0.1586	-0.8722	-0.0461	-0.4080	0.5162	0.4656	0.4434	0.2247	
PC 16:0_22:5	-0.3323	-0.1880	0.2641	-0.2780	-0.6551	-0.6460	-0.9805	-0.4811	
PC 16:0_22:6	0.4983	0.4305	0.5285	0.4679	-1.0784	-1.2045	-1.2224	-1.2186	x
PC 16:1_16:1_1	0.3038	1.1530	0.3642	1.2097	-1.1638	-1.2535	-1.2518	-1.2416	

PC 16:1_18:2	-0.2800	-0.4284	-0.2368	-0.4000	0.8618	0.8257	0.6762	0.6539	
PC 16:1_22:6	0.6796	-0.5840	-0.6008	-0.5784	1.1221	0.3408	1.0832	1.0848	x
PC 18:0_18:1	0.3340	-0.0050	-0.1266	0.1217	-0.3340	-0.2835	-0.4884	-0.3391	
PC 18:0_20:3	-0.3431	-0.5626	-0.0643	-0.3060	0.9170	0.5146	0.5631	0.6280	
PC 18:0_20:4	-0.2483	-0.5361	-0.3337	-0.2747	0.5155	0.1679	0.2232	0.2481	
PC 18:0_22:6	0.4276	0.3464	0.4312	0.3972	-0.9047	-1.0764	-1.1001	-1.0865	x
PC 18:1_18:2	-0.3375	-0.5818	-0.1095	-0.4202	0.7304	0.5182	0.4629	0.5140	
PC 18:1_20:2	-0.5425	-0.6822	0.0518	-0.9250	0.8788	0.8434	0.0970	0.6724	
PC 18:1_22:5	0.0458	0.1612	0.3529	0.0512	-0.4009	-0.5302	-1.3718	-1.3374	
PC 18:1_22:6	0.4716	0.4001	0.5500	0.4578	-0.9646	-1.0938	-1.3109	-1.1955	x
PC 18:2_18:2	-0.2811	-0.4851	-0.2017	-0.4020	0.7240	0.6240	0.5301	0.5867	
PC 18:2_22:6	0.3664	0.1667	0.4776	0.2609	-0.6495	-0.7420	-0.8170	-0.8149	x
PC 19:0_22:6	-0.5649	-0.6899	-0.0578	0.3109	0.7656	0.4171	0.5859	0.5467	x
PC 2:0_22:6	0.3737	0.3783	0.4586	0.0924	-0.8499	-0.9845	-0.9819	-0.9667	x
PC 20:0_22:6	0.3595	0.1380	0.4347	0.2422	-0.3939	-0.6039	-0.9889	-1.5736	x
PC 20:1_22:6	0.3814	0.2711	0.3967	0.3400	-0.9604	-0.8524	-0.8507	-0.7487	x
PC 20:3_22:6	0.4986	0.4285	0.3681	0.4693	-0.2745	-1.5725	-0.6064	-1.1151	x
PC 20:4_22:6	0.3410	0.2100	0.4458	0.3443	-0.9657	-1.1242	-1.1606	-1.1586	x
PC 20:5_22:6	0.5811	0.4834	0.6590	0.4859	-1.4366	-1.5327	-1.5309	-0.7726	x
PC 22:0_22:6	0.5614	0.3567	0.4618	0.3990	-0.6499	-0.7335	-1.9602	-1.1077	x
PC 22:5_22:6_1	0.3875	0.2469	0.4222	0.4173	-1.1940	-1.4588	-1.2360	-1.3177	x
PC 22:6_22:6	0.7420	0.6953	0.8019	0.7217	-1.5918	-1.7653	-1.9353	-1.9718	x
PC 26:0_26:2	-0.3775	-1.2292	-1.2434	-1.2245	1.2835	0.8237	1.0805	0.8596	
PC 26:0_26:4	-0.6807	-1.0018	-0.4857	-0.5119	1.3099	1.1565	1.1461	1.1830	
PC 26:1_26:2	-0.4527	-0.6426	-1.1306	-0.5482	1.1930	0.9902	1.0679	1.1072	
PC 26:1_26:4	-0.8190	-0.5411	-0.5019	-0.2955	1.2170	1.0041	1.0628	1.0952	
PC 26:2_26:4	-0.2660	-0.3954	-0.1747	-0.4672	0.9670	0.2168	0.3431	0.8087	
PC 26:4_22:6	0.2385	0.1204	0.1879	0.1139	-0.8033	-0.8340	-0.8133	-1.6405	x

PC 26:4_26:4	0.4702	0.1199	0.2937	0.2553	-0.1012	-0.4068	-1.6434	-0.6998	
PC 29:0	-0.2315	-0.4961	-0.2426	-0.3554	0.8944	0.6270	0.6207	0.7029	
PC 31:1	-0.3397	-0.4120	-0.2714	0.1284	0.8022	0.4352	0.6832	0.4814	
PC 33:0	-0.5655	-0.6752	-0.1776	-0.4838	0.6147	0.3034	0.2966	0.4181	
PC 34:0	-0.3608	-0.5288	0.2201	-0.2057	0.6380	0.1685	0.1934	0.2265	
PC 34:3	-0.2896	-0.5614	-0.2463	-0.2589	0.9743	0.7011	0.7803	0.8161	
PC 34:4	0.0645	0.0029	0.4780	-0.1847	-0.4807	-0.5477	-0.6388	0.0332	
PC 35:0_1	-0.0923	-0.8026	0.0108	-0.4283	0.5620	0.3649	0.3419	0.3391	
PC 35:7	-0.3523	-0.5999	-0.0338	-0.3327	0.7705	0.4773	0.4660	0.5549	
PC 36:5	-0.1129	-0.0981	0.1321	-0.6388	0.6607	0.4322	0.2478	0.4115	
PC 36:5_1	0.4072	0.2303	0.6389	0.7302	-0.4319	-0.9744	-0.8044	-0.4665	
PC 36:6	0.3692	0.3637	0.5463	0.4089	-1.0205	-0.9883	-1.1057	-1.2505	
PC 36:8	0.4570	0.1714	0.3206	0.2368	-0.8067	-0.9475	-0.9447	-0.9288	
PC 38:2	-0.1869	-0.6529	-0.4079	-0.2256	0.5623	0.1108	0.0649	0.2225	
PC 38:4	-0.2406	-0.9097	-0.1496	-0.3885	0.8589	0.6668	0.5961	0.5454	
PC 38:6	-0.4583	-0.5276	-0.5477	-0.5211	0.7175	0.7184	0.6565	0.7607	
PC 38:7	0.3421	0.4223	0.6179	0.5131	-1.0024	-1.1548	-0.5820	-1.1807	
PC 38:7_1	0.5032	0.4920	0.5961	0.4745	-0.6310	-0.8915	-0.6180	-1.8137	
PC 38:9	0.2922	0.3038	0.3344	0.2168	-0.8112	-0.9511	-0.9484	-0.9326	
PC 39:6	0.3233	0.3246	0.3961	0.2290	-0.6329	-1.0276	-0.9041	-0.9359	
PC 40:3	-0.7514	-0.7909	-0.1600	-0.7872	1.5957	1.6350	1.5649	1.3968	
PC 40:5	-0.2682	-0.4759	-0.3065	-0.2981	0.7560	0.3692	0.4554	0.5447	
PC 40:5_1	-0.2864	-0.3042	-0.3253	-0.3168	0.7661	0.3626	0.4675	0.5154	
PC 40:7	-0.3699	-0.5852	-0.2228	-0.3931	0.8472	0.5910	0.5794	0.6256	
PC 40:7_1	0.0764	0.0009	1.0353	0.3136	-0.2003	-1.7724	-0.4110	-0.6484	
PC 40:9	0.1784	0.1705	0.6583	-0.1033	-0.7019	-0.9132	-0.6789	-0.6617	
PC 41:6	0.3398	0.1819	0.2700	0.0347	-0.4245	-0.8307	-0.8571	-1.3435	
PC 42:5	-0.5755	-0.4385	-0.5791	-0.8940	0.1777	0.3795	0.2788	0.2702	

PC 42:6	0.1418	-0.1180	0.2897	0.1283	-0.5836	-0.6942	-0.8449	-0.6145	
PC 42:8	0.4789	0.3476	0.4863	0.4062	-1.4263	-1.9439	-0.5563	-0.7904	
PC 42:9	0.0109	0.2643	0.3160	0.0619	-0.4474	-0.7180	-1.1377	-0.6272	
PC 44:2	0.3113	0.2103	0.4078	0.2034	-0.8510	-0.8117	-1.2582	-0.8726	
PC 44:7	0.4331	-0.0757	0.6639	0.1290	-0.3547	-1.4683	-0.7900	-0.7188	
PC 44:7_1	-1.1106	-0.0953	-0.3000	-0.2858	0.6695	0.5769	0.4037	0.6401	
PC 46:6	0.3508	-0.5064	0.7481	0.6774	-0.5988	-1.4369	-1.4350	-1.4242	
PC 46:6_1	0.3905	0.2567	0.4910	0.4841	-0.5492	-1.0156	-1.0001	-1.8529	
PC 46:7_1	0.4264	-0.1116	0.2891	0.2514	-0.4720	-1.5036	-1.5014	-0.3077	
PC 46:9	-0.5168	-0.2097	-0.5081	-0.1661	0.6491	0.3450	0.1132	0.4763	
PC 48:6	0.4809	0.3817	0.5087	0.4051	-0.8178	-1.7352	-0.9652	-0.9654	
PC 48:7	0.7679	0.7224	0.1462	0.5327	-0.5378	-1.7503	-0.7300	-0.8112	
PC 50:3	-1.1987	-1.2410	-0.0431	-0.4689	1.3530	1.3299	1.3042	1.3477	
PC 50:4	-0.6146	-0.6998	-0.4911	-0.7946	1.1590	1.0020	1.0764	1.0948	
PC 50:5	-0.3465	-0.4872	-0.2162	-0.3159	0.9524	0.5770	0.7055	0.7479	
PC 50:6	0.2749	0.2441	0.4581	0.2879	-0.6245	-1.7924	-0.9985	-0.0272	
PE 14:0_16:0	-0.4383	-0.3083	-0.1797	-0.2597	0.9505	0.5024	0.5464	0.2795	
PE 14:0_18:2	-0.4688	-0.2663	-0.4661	-0.2500	1.1768	0.7472	0.7233	0.5668	
PE 15:0_22:6	0.4327	0.2060	0.2300	0.2991	-0.2164	-0.6843	-0.5090	-0.6367	x
PE 16:0_14:1	-0.6250	-0.3905	-0.2445	-0.2814	0.7236	0.5881	0.6880	0.7582	
PE 16:0_16:1	-0.2220	-0.5884	-0.1443	-0.5108	0.7769	0.8754	0.8714	0.9082	
PE 16:0_16:1_1	-0.1948	-0.3594	-0.1101	-0.5083	1.0297	0.6317	0.7448	0.6752	
PE 16:0_18:2	-0.3875	-0.3817	-0.3458	-0.3613	0.9715	0.6955	0.7418	0.7468	
PE 16:0_22:4	-0.4493	-0.5986	-0.4704	-0.5029	0.4178	0.6832	0.7390	0.7322	
PE 16:0_22:5	-0.4712	-0.5662	-0.6287	-0.7134	0.3262	0.0115	0.0500	0.0112	
PE 16:0_22:6	0.3191	0.2405	0.2714	0.2391	-0.4360	-0.7440	-0.7001	-0.7013	x
PE 16:1_18:2	-0.3827	-0.3881	-0.4191	-0.3426	1.0267	0.8794	0.8624	0.7975	
PE 16:1_20:4	-0.1332	-0.1758	-0.1479	-0.1289	0.6495	0.4882	0.4888	0.4541	

PE 18:0_18:1	-0.1716	-0.4414	-0.2353	-0.3772	0.7431	0.2765	0.3550	0.3585	
PE 18:0_18:2	-0.2948	-0.4356	-0.3130	-0.3971	0.8215	0.5490	0.6193	0.6488	
PE 18:0_18:2_1	0.0132	-0.1637	-0.5733	-0.2471	0.9132	0.5732	0.0842	0.4959	
PE 18:0_20:1	-0.2831	-0.6964	-0.3614	-0.4347	0.6549	0.4168	0.3626	0.2759	
PE 18:0_20:3_1	-0.1123	-0.4025	-0.0553	-0.4909	0.9273	0.6734	0.5726	0.3424	
PE 18:0_20:4_2	0.6241	0.6568	0.2246	0.4952	-1.6207	0.0469	-0.8941	-1.7135	
PE 18:0_22:3	-0.1207	-0.4860	-0.3104	-0.3872	0.7064	0.4114	0.3371	0.3827	
PE 18:0_22:4	-0.4340	-0.6227	-0.4164	-0.4911	0.6029	0.2733	0.3517	0.3250	
PE 18:0_22:5_1	0.3416	0.2512	0.3023	0.2450	-0.6113	-0.8985	-0.7907	-0.8123	
PE 18:0_22:6_1	0.2963	0.5031	0.2188	0.4099	-1.3723	-0.7359	-0.6176	-0.5911	x
PE 18:1_18:2	-0.3581	-0.4832	-0.3521	-0.5025	0.9379	0.6671	0.7158	0.5334	
PE 18:1_20:4	-0.3925	-0.5692	-0.1282	-0.2698	0.8217	0.6084	0.5401	0.4123	
PE 18:1_22:5	-0.9099	-0.4378	-0.1256	-0.2658	0.6303	0.3364	0.2928	0.3298	x
PE 18:1_22:6	0.2795	0.2016	0.1672	0.1689	-0.1042	-0.6137	-0.4620	-0.5241	
PE 18:2_18:2	-0.1982	-0.2684	-0.3288	-0.3308	0.6550	-0.1131	0.4820	0.4811	
PE 18:2_22:5	0.0062	-0.1901	-0.3066	-0.1250	0.5349	0.3173	0.4176	0.2933	
PE 18:3_22:6	-0.1526	-0.1674	-0.4791	-0.0795	0.7449	0.2899	0.5186	0.5152	x
PE 18:3_22:6_1	-0.0937	-0.1829	-0.2818	-0.1921	0.7537	0.2496	0.4939	0.4782	x
PE 18:4_22:6	0.4358	0.3866	0.6049	0.4268	-0.1772	-1.4680	-1.4656	-0.4685	x
PE 19:0_22:6	0.2016	-0.3410	0.3978	0.1987	-0.7109	-0.4537	-0.5159	-1.1277	x
PE 20:0_22:6	0.5682	0.5161	0.1893	0.5501	-0.6628	-0.8789	-1.0105	-1.5194	x
PE 20:0_22:6_1	0.3580	0.3070	0.2793	0.1848	-0.6430	-0.7141	-0.9892	-0.6689	x
PE 20:1_20:4	-0.0032	-0.0891	-0.1685	-0.2302	0.8704	0.8060	0.7770	-0.0433	
PE 20:4_22:6	0.1128	-0.1210	-0.0741	0.0650	-0.4653	-0.7347	-0.6753	-0.6729	x
PE 20:4_24:4	-0.4307	0.2192	-0.6100	-0.5169	1.0857	0.8806	0.9949	0.9681	
PE 20:5_22:6	0.5073	0.4968	0.5444	0.5107	-1.2537	-1.1392	-1.1892	-1.2597	x
PE 22:4_22:6	0.2082	0.1232	0.1242	0.1102	-0.5047	-0.8475	-0.7333	-0.7607	x
PE 22:5_22:6	0.1239	0.1393	-0.0401	-0.0426	-0.9640	-1.2276	-1.1476	-1.1306	x

PE 22:5_22:6_1	0.4825	0.4423	0.4081	0.4108	-0.7936	-1.0484	-0.9331	-0.9494	x
PE 22:6_22:6	0.6560	0.6304	0.6203	0.6099	-1.4268	-1.5671	-1.5223	-1.5640	x
PE 24:4_22:6	-0.5242	-0.6149	-0.5036	-0.3320	0.5978	0.1916	0.2479	0.3353	x
PE 36:4	-0.2511	-0.4038	-0.2700	-0.3085	0.9506	0.6875	0.5029	0.8044	
PE 36:5	-0.0994	-0.9930	0.0591	-0.1764	0.7672	0.8023	0.6633	0.5924	
PE 37:6	0.2025	-0.2130	0.2814	-0.0412	-0.0847	-0.4553	-0.6076	-0.3967	
PE 38:7_1	0.3280	0.3930	0.6294	0.3069	-0.1682	-0.4834	-0.3648	-1.9455	
PE 39:6	-0.2073	0.2000	0.2645	0.2912	-0.1868	-0.5461	-0.8498	-0.6173	
PE 39:7	0.4696	-0.1284	-0.0060	0.3140	-0.2620	-0.7378	-0.4100	-0.4331	
PE 40:4	-0.4634	-0.6873	-0.1833	-0.3521	0.6086	0.2730	0.3637	0.3107	
PE 40:7	-0.3782	-0.4477	-0.4678	-0.4412	0.9635	0.9437	0.9494	0.6743	
PE 40:7_1	0.5014	0.4233	0.5413	0.4775	-1.0114	-1.3064	-1.1616	-1.3077	
PE 42:6	-0.5062	-0.7766	-0.3133	-0.4066	0.8925	0.4469	0.6102	0.5726	
PE 42:7	-0.4329	-0.4893	-0.5055	-0.4839	1.8946	0.4048	0.8383	0.8613	
PE 42:9	0.1582	0.0069	0.0686	0.0409	-0.3395	-0.3923	-0.5590	-0.4786	
PE 44:7	-0.4367	-0.4957	-0.5127	-0.4901	1.3647	0.4394	1.1841	1.0501	
PG 14:0_16:0	-0.2491	-0.3752	-0.1495	-0.4721	0.8647	0.3214	0.4873	0.4169	
PG 16:0_16:0	-0.4114	-0.4135	0.1678	-0.3991	0.4644	0.3216	0.5736	0.4451	
PG 16:0_20:4	0.2935	-0.1850	0.5165	0.0448	-0.3528	-0.2690	-0.5564	-0.4524	
PG 16:0_22:6	0.3658	0.3978	0.4713	0.4252	-0.7783	-1.0119	-0.9372	-1.1159	x
PG 18:0_22:6	0.3488	0.1724	0.5740	0.1617	-0.3840	-1.2783	-0.3206	-0.3394	x
PG 18:1_18:1	-1.6144	-0.4732	-0.2288	-0.0329	1.3617	1.0234	1.2092	0.9829	
PG 18:1_20:3	-0.3934	-0.3768	-0.1917	-0.2535	0.8703	0.7220	0.7188	0.4868	
PG 18:1_20:4_1	0.0323	-0.1501	-0.0523	-0.1220	0.7929	0.5871	0.7140	0.5194	
PG 18:1_22:4	-0.2923	-0.4889	0.1141	0.3401	0.6112	0.8201	0.5430	0.3114	
PG 18:1_22:6	0.1321	-0.2171	0.3351	0.2513	-0.4604	-0.7099	-0.6626	-0.7259	x
PG 18:2_18:2	-0.1492	-0.6893	0.0049	-0.2408	0.9025	0.8592	0.8249	0.6843	
PG 18:2_20:3	-0.1481	-0.1168	0.0710	-0.1816	0.8547	0.5939	0.6520	0.5046	

PG 18:2_22:6	0.4620	0.2316	1.0120	0.2071	-0.3243	-0.4663	-0.5369	-0.5427	x
PG 20:4_22:5	-0.2636	-0.2023	0.0016	-0.1518	-0.4496	-0.8828	-0.6089	-0.5286	
PG 20:4_22:6	0.3823	0.3140	0.2807	0.3247	-0.5967	-0.6492	-0.7277	-0.8716	x
PG 22:4_22:4	-0.9642	-0.5547	-0.0440	-0.8447	0.6490	0.3097	0.7191	0.4167	
PG 22:5_22:5	-0.3726	-0.3976	-0.2789	-0.3305	-0.6974	-0.5811	-0.8765	-0.8166	
PG 22:5_22:6	0.0425	-0.0902	-0.0442	0.0295	-0.9399	-0.9182	-0.8314	-0.7928	x
PG 22:6_22:6	0.6573	0.6157	0.6212	0.6026	-1.2499	-1.5867	-1.4388	-1.4651	x
PI 16:0_22:6	0.3948	0.2879	0.9125	0.2750	-0.8051	-0.9429	-0.9402	-0.9246	x
PI 18:0_20:3_1	-0.2009	-0.3744	0.2951	-0.5454	0.8391	0.3614	0.5393	0.4789	
PI 18:0_22:6	0.3203	0.2707	0.8772	0.1038	-0.4751	-1.2123	-0.9142	-0.9212	x
PI 20:4_22:6	0.3036	0.1903	0.6073	0.1162	-0.8309	-0.9663	-0.9637	-0.9484	x
PMeOH 16:0_22:6	0.5550	-0.2718	0.2460	0.4841	-0.6044	-0.7404	-1.2292	-0.7265	x
PS 18:0_18:2	-1.9018	-1.1466	-0.5249	-0.8615	0.9467	0.1532	0.8361	0.8873	
PS 18:0_20:3	0.0789	-0.0447	0.0460	0.1323	-0.1861	-0.6059	-0.4285	-0.4840	
PS 18:0_22:4	-0.1774	-0.8334	0.1732	-0.6221	0.9291	0.1009	0.5139	0.6673	
PS 18:0_22:5	-0.6445	-1.3342	-0.3111	-1.6385	0.5946	0.2233	0.2860	0.3455	
PS 18:0_22:6	0.1694	-0.0814	1.0150	-0.0309	-0.3391	-0.9647	-0.6551	-0.6015	x
PS 18:1_20:4	-0.2056	-0.4164	0.4163	-0.6979	0.9048	0.4286	0.6068	0.2291	
PS 22:5_22:6	0.2919	0.3241	1.0463	0.2957	-0.6777	-0.9285	-0.9197	-0.8154	x
PS 22:5_22:6_1	0.3154	0.0748	0.4425	0.2972	-0.6438	-1.0178	-1.4243	-1.1074	x
PS 22:6_22:6	0.5265	0.4509	0.9173	0.4017	-1.1892	-1.4233	-1.3927	-1.2576	x
PS 24:4_22:6	-0.4906	-0.6945	0.0094	-0.6545	0.9115	0.5124	0.7393	0.8404	x
PS 40:5	-1.0499	0.2861	-1.1074	-0.0247	1.1027	1.0398	1.0545	0.9011	
SM d18:1_18:0	0.1230	-0.0488	0.1159	0.0231	-0.1076	-0.4930	-0.5207	-0.4397	
SM d32:1	-0.1514	-0.3096	0.1126	-0.0925	0.6498	0.1901	0.1794	0.4616	
SM d36:2	-0.2460	-0.3953	0.1656	-0.0871	0.7298	0.3183	0.3340	0.3931	
SM d36:2_1	-0.2309	-0.4819	-0.0177	-0.3093	0.7954	0.6883	0.5968	0.7169	
SM d38:1	0.0411	-0.1694	0.1932	0.0189	-0.1159	-0.5035	-0.5452	-0.5017	

SM d42:3	0.1552	0.1865	-0.0055	-0.0156	-0.3918	-0.7076	-0.5691	-0.5294	
TG 16:0_16:0_18:0	-0.4270	-0.4606	-0.3981	-0.1771	0.9891	0.5900	0.4335	0.5972	
TG 16:0_20:4_22:6	0.3477	0.2028	0.3332	0.3301	-0.7367	-1.1284	-1.0023	-0.7431	x
TG 16:0_22:5_22:7	0.5889	0.5289	0.5490	0.5618	-0.9248	-1.1656	-1.4617	-1.7356	
TG 16:0_22:6_22:6	0.6045	0.5467	0.5675	0.5894	-0.8445	-1.0806	-1.8820	-1.6280	x
TG 18:0_22:6_22:6	0.3247	0.2372	0.3575	0.3212	-0.7757	-0.9214	-0.9186	-0.9022	x
TG 22:6_22:6_22:6	0.3481	0.2555	0.2833	0.3406	-1.0320	-1.0576	-0.9371	-0.7803	x
TG 50:0	-0.4326	-0.4509	-0.4032	-0.1839	0.9768	0.5795	0.5072	0.5867	
TG 52:6	0.4756	0.2630	-0.0821	0.3562	-1.3946	-0.3742	-0.5853	-1.4980	
TG 56:6	-0.6288	-0.1558	-0.3834	-0.1896	0.5807	0.2544	0.3696	0.0405	
TG 56:7	0.4486	0.1423	0.2677	0.3341	-0.3023	-0.9189	-1.3539	-1.0698	
TG 58:10	0.3491	0.2045	0.3276	0.3316	-0.7334	-1.1244	-1.0181	-0.7397	
TG 58:7	0.3655	0.3655	0.4022	0.2714	-1.3443	-1.3746	-0.4628	-0.2387	
TG 60:10	0.2130	-0.2696	0.2013	0.2563	-0.6236	-1.0481	-1.0174	-0.5810	
TG 60:11	-0.0852	0.2152	0.2319	0.1222	-1.0130	-1.9195	-0.8397	-0.6289	
TG 60:11_1	0.5571	0.3571	0.5505	0.2564	-0.7961	-1.2089	-0.4957	-0.7650	
TG 62:12	0.5592	0.5035	0.5820	0.5640	-1.0537	-0.7356	-1.8469	-1.8367	
TG 62:13	0.5258	0.4654	0.4687	0.4792	-2.0014	-1.3025	-1.0720	0.1379	

Red denotes upregulation, blue denotes downregulation.

Supplementary Table 8: Significantly-Altered Lipids in *Tmem135*$^{FUN025/FUN025}$ (*FUN025*) Hearts Compared to WT.

Lipid Species	Normalized Log10 Lipid Concentrations								Contains DHA
	WT 1	WT 2	WT 3	WT 4	*FUN025* 1	*FUN025* 2	*FUN025* 3	*FUN025* 4	
CE 20:4	0.2517	0.3937	0.6052	0.2445	-1.0339	-0.0891	-0.6064	-0.4373	
CE 22:6	0.6773	0.6948	0.5588	0.7370	-1.2261	-1.1880	-1.2241	-0.2933	x
Cer_NS d18:1_17:0	0.2929	0.5240	0.8362	0.7754	-0.3708	-0.2759	-0.2416	-0.3365	
Cer_NS d18:1_26:0	0.0741	0.5680	0.6043	-0.0146	-0.4459	-0.4403	-0.4742	-0.2195	
Cer_NS d18:2_18:0	0.2090	0.3984	0.6488	0.7574	-0.2090	-0.2235	-0.0828	-0.3236	
Cer_NS d18:2_18:0_1	0.2090	0.3984	0.6487	0.7574	-0.2089	-0.2234	-0.0827	-0.3235	
Cer_NS d18:2_19:0	0.3003	0.4790	0.6066	0.7574	-0.9169	-1.1681	-0.3625	-0.4080	
Cer_NS d37:1	0.1706	0.6418	0.8063	0.5630	-0.7079	-0.1284	-0.1498	-0.0027	
Cer_NS d41:2	0.0967	0.2343	0.4914	0.2147	-1.3730	-0.8127	0.1168	-1.1787	
CL 68:6	-0.3825	0.1860	0.0596	-0.0730	1.2600	0.1838	0.5783	0.8310	
CL 70:7	0.0396	-0.0748	-0.0008	-0.0944	1.1100	0.7933	0.5043	1.1422	
CL 70:8	0.0775	0.1835	0.1473	0.0318	1.3527	0.9439	0.8584	1.1849	
CL 72:10	0.0146	-0.1884	-0.0611	-0.1724	1.1761	0.7964	0.5344	1.2471	
CL 72:8	-0.0638	0.0679	-0.0887	-0.3266	1.4443	0.4444	0.4247	1.0614	
CL 72:9	-0.1527	0.0245	-0.0555	-0.4524	1.0092	0.4260	0.2013	0.7004	
CL 74:11	0.0156	0.0769	-0.0308	-0.1953	1.0703	0.3989	0.3526	0.8054	
CL 74:7_1	0.2340	-0.1386	0.1369	0.0725	0.0246	-0.3933	-0.5935	-0.7328	
CL 76:11	0.5852	0.5734	0.3939	0.6736	-0.5222	-0.6166	-1.3361	-0.5027	
DG 16:0_22:6	0.7857	0.6227	0.5509	0.6520	-1.9924	-1.1431	-1.0470	-0.1976	x
DG 18:1_22:5	0.2562	0.5822	0.5694	0.2552	-1.0176	-1.5075	-1.3716	-1.0701	
DG 18:2_20:4	0.7763	0.6696	0.5909	0.6107	-0.1588	-0.6810	-0.9198	-0.2933	
DG 18:2_22:5	0.4512	0.5593	0.5209	0.2772	-1.1326	-1.0658	-1.9087	-0.8285	
DG 18:2_22:6	0.7862	0.7531	0.6584	0.3757	-0.7261	-0.9502	-1.0605	-1.0290	x
EtherPC 16:0e_20:4	-1.0412	-0.9705	-0.9300	-0.0032	1.0005	0.8085	0.4377	1.1862	
EtherPC 16:0e_22:5	0.6144	0.2642	0.3629	0.7501	-0.0435	-0.0997	-0.0186	-0.1915	

EtherPC 16:1e_18:1	-0.1296	-0.8817	-0.8472	-0.8823	0.8069	0.6194	1.5048	1.7250	
EtherPC 16:1e_18:2	-0.8213	-0.1282	-0.5307	-0.4321	0.4562	1.2838	1.3369	1.4548	
EtherPC 18:1e_22:6	-0.8790	0.5727	-0.7760	-0.8142	0.7139	0.8278	0.9047	1.0711	x
EtherPC 18:2e_22:5	-2.0250	-0.0931	0.1133	-0.2835	0.6653	0.7643	0.6468	0.7933	
EtherPC 39:7e	-0.7447	-0.6907	0.0216	-0.6912	1.7419	1.1377	1.0242	1.7805	
EtherPE 14:1e_20:4	0.4231	0.3646	0.3048	0.4945	-0.3158	-0.4872	-0.4544	-0.3831	
EtherPE 16:1e_16:0	-0.3026	-0.2622	-0.2145	-0.2464	0.9391	0.5807	0.5769	0.8284	
EtherPE 16:1e_16:1	0.1340	-0.8383	-0.3370	0.3645	1.0561	0.9660	0.9405	1.0718	
EtherPE 16:1e_18:1	-0.0136	-0.1836	-0.1692	0.1950	0.8643	0.5590	0.5392	0.7186	
EtherPE 16:1e_18:2	-0.4537	-0.3402	-0.3027	-0.4302	1.1468	0.9622	0.9560	1.0936	
EtherPE 16:1e_18:3	-0.1238	-0.2556	-0.2299	-0.3637	1.0287	0.8512	0.8275	0.9573	
EtherPE 16:1e_20:1	-0.0855	-0.2179	-0.2774	0.4747	0.8517	0.5585	0.4774	0.6855	
EtherPE 16:1e_22:3	-0.1554	-0.1924	-0.2676	-0.0980	1.1204	0.7114	0.8696	0.7577	
EtherPE 16:1e_22:4	-0.0395	0.0622	-0.0410	0.1251	0.6889	0.5434	0.3364	0.6536	
EtherPE 16:1e_22:5	-0.1744	0.0622	-0.0029	-0.1518	-0.4087	-0.6338	-0.8012	-0.4188	
EtherPE 16:2e_20:4	0.5047	0.4011	0.5570	0.8593	-0.7464	-0.4654	0.5999	-0.5785	
EtherPE 18:0e_18:2_1	0.0446	0.0753	-0.1392	-0.0219	0.6398	0.4941	0.4725	0.5832	
EtherPE 18:1e_16:0	-0.7386	-0.0724	-0.1203	0.1291	0.9756	0.7655	0.6702	0.8621	
EtherPE 18:1e_18:2	-0.6618	0.0964	-0.0160	0.2046	0.7968	0.3854	0.3897	0.6678	
EtherPE 18:1e_22:3	-0.7238	-0.0611	0.3527	-0.8888	1.2978	0.3919	0.1191	0.7554	
EtherPE 18:1e_22:5	0.1541	0.2566	0.1447	0.1709	-0.6347	-0.6109	-1.8479	-0.8658	
EtherPE 18:1e_22:5_1	0.4306	0.7535	0.5434	0.4841	0.0509	-0.0150	-0.6885	-0.2545	
EtherPE 18:1e_22:6	0.5052	0.6953	0.6228	0.4972	-0.8267	-1.2947	-1.3539	-1.0075	x
EtherPE 18:1e_22:6_1	0.5176	0.7089	0.7776	0.5066	-0.6516	-1.6511	-1.0489	-0.7565	x
EtherPE 18:2e_18:2	-0.4251	-0.2880	-0.2291	-0.4634	1.0799	0.9218	0.8826	0.9995	
EtherPE 18:2e_22:5	0.0173	0.2300	0.2334	-0.0899	-0.5472	-0.8319	-0.6859	-1.1756	
EtherPE 18:2e_22:6	0.6002	0.7768	0.7219	0.5925	-1.0347	-1.0110	-1.0922	-0.9131	x
EtherPE 18:3e_18:2	-0.3610	-0.1238	-0.1209	-0.3917	0.9375	0.7567	0.6866	0.9111	

EtherPE 18:3e_20:4	0.0119	-0.7481	-0.2197	-0.0418	0.9389	0.5926	0.5555	0.8916	
EtherPE 18:3e_22:6	0.5446	0.7007	0.6612	0.5696	-0.2593	-1.7816	-0.8249	-0.5413	x
FA 16:0_1	-0.3600	-0.9209	0.3199	-0.3740	1.0895	0.7489	0.7796	0.8850	
FA 18:0_1	-0.1440	0.3094	-0.0154	-0.0483	1.0644	0.7813	0.5430	0.7133	
FA 18:1_1	-0.4221	-0.0398	-0.0772	-0.2780	1.2384	1.0476	0.9364	1.1057	
FA 18:1_3	-0.4211	-0.0369	-0.0745	-0.2763	1.2473	1.0558	0.9439	1.1139	
FA 18:2_1	-0.9969	-0.1784	-0.4108	-0.2844	1.0804	0.9482	0.9740	1.0917	
FA 18:2_3	-0.1829	0.6355	-0.1149	-0.9035	0.8118	0.6528	1.4373	1.5375	
FA 18:2_5	-0.4533	-0.3633	-0.3117	-0.3641	0.9779	0.8462	0.7517	0.9430	
FA 18:2_6	-0.4486	-0.3574	-0.3052	-0.3583	1.0504	0.8173	0.7748	0.8266	
FA 18:2_8	-0.9995	-0.1801	-0.4128	-0.2862	1.0802	0.9478	0.9697	1.0930	
FA 20:1_1	-0.4624	-0.3750	-0.3250	-0.3759	1.0415	0.7515	0.8255	0.9985	
FA 22:4	-0.0113	0.9043	0.9993	0.2881	-0.8044	-0.7168	-0.8534	-0.7866	
FAHFA 22:4_18:2	0.0541	0.5944	0.7422	0.4078	-0.5510	-0.5456	-0.6993	-0.3234	
FAHFA 22:5_20:4	0.0829	0.9680	1.0091	0.3650	-0.5089	-0.3036	-0.5111	-0.5419	
LPC 0:0/16:0	-0.5634	0.0716	0.2331	-0.2997	0.3994	0.5463	0.4106	0.4894	
LPC 0:0/16:1	-0.7251	-0.3958	-0.0921	-0.2970	0.4630	0.6804	0.5766	0.5749	
LPC 0:0/18:1	-0.7583	-0.0845	0.1774	-0.2686	0.5741	0.6925	0.5417	0.5296	
LPC 0:0/18:2	-0.7556	-0.2336	-0.0674	-0.5345	0.8159	0.8967	0.7497	0.8380	
LPC 0:0/20:3	-0.8803	-0.3285	0.2385	-0.0720	0.4655	0.8118	0.3888	0.6321	
LPC 0:0/20:4	-0.1084	0.5230	0.6313	0.2717	-0.7349	-0.5846	-0.8340	-0.6225	
LPC 0:0/22:4	-0.2427	0.4877	0.5446	0.0947	-0.5851	-0.3044	-0.5845	-0.5689	
LPC 0:0/22:5	0.2874	0.6275	0.6595	0.4124	-1.6871	-1.6598	-1.6857	-1.0321	
LPC 0:0/22:6	0.5691	0.9488	0.9729	0.7089	-1.3883	-1.2216	-1.3509	-2.0355	x
LPC 16:0/0:0	-0.2935	0.0861	0.2368	-0.1960	0.5015	0.6588	0.4684	0.6409	
LPC 16:1/0:0	-0.4051	-0.1779	-0.0024	-0.3880	0.9323	1.0826	0.9262	0.9698	
LPC 18:1/0:0	-0.3240	0.0859	0.2753	-0.1833	0.5337	0.5931	0.5039	0.6156	
LPC 18:2/0:0	-0.4255	-0.0933	0.0074	-0.4681	0.7800	0.8778	0.7849	0.8066	

**LPC 19:0/0:0**	**-0.2340**	**0.0690**	**0.0999**	**-0.1882**	**0.3630**	**0.4594**	**0.1658**	**0.5805**	
**LPC 20:0/0:0**	**-0.1852**	**0.0229**	**-0.1181**	**-0.0776**	**0.3918**	**0.6597**	**0.4462**	**0.7945**	
**LPC 20:1/0:0**	**-0.3980**	**0.0032**	**0.1419**	**-0.2497**	**0.6471**	**0.5955**	**0.3399**	**0.6859**	
**LPC 22:5/0:0**	**0.0611**	**0.7335**	**0.9483**	**0.3833**	**-0.6376**	**-0.5835**	**-0.8721**	**-0.7852**	
**LPC 22:5/0:0_1**	**-0.0770**	**0.3663**	**0.3849**	**0.0515**	**-0.9920**	**-0.9510**	**-0.9898**	**-0.9813**	
**LPE 16:0**	**-0.3298**	**0.0440**	**-0.0175**	**-0.2608**	**1.1315**	**0.7611**	**0.6848**	**0.8818**	
**LPE 16:1**	**-0.1963**	**0.1081**	**0.0652**	**-0.0426**	**1.2617**	**1.1026**	**0.9829**	**1.1264**	
**LPE 16:1_1**	**-0.1958**	**0.1087**	**0.0658**	**-0.0421**	**1.2627**	**1.1036**	**0.9759**	**1.1276**	
**LPE 17:0**	**-0.2036**	**0.1361**	**0.2245**	**-0.1426**	**1.0381**	**0.6946**	**0.6012**	**0.8951**	
**LPE 17:1**	**-0.4963**	**-0.4182**	**-0.3735**	**-0.4190**	**1.2170**	**1.0781**	**0.5496**	**1.1355**	
**LPE 18:0**	**-0.2520**	**0.0801**	**0.0382**	**-0.0593**	**0.9448**	**0.8481**	**0.5470**	**1.0609**	
**LPE 18:1**	**-0.3345**	**-0.1544**	**-0.1093**	**-0.3117**	**1.3035**	**1.0174**	**0.9087**	**1.0905**	
**LPE 18:2**	**-0.6992**	**-0.2027**	**-0.1283**	**-0.5137**	**1.2158**	**1.0716**	**0.9941**	**1.0950**	
**LPE 18:2_1**	**-0.2409**	**0.0855**	**0.0516**	**-0.2284**	**1.1485**	**0.9350**	**0.8624**	**1.1232**	
**LPE 19:0**	**-0.1671**	**0.2083**	**0.0562**	**-0.2406**	**0.9580**	**0.6794**	**0.6120**	**0.7857**	
**LPE 20:0**	**-0.5922**	**0.4771**	**-0.3417**	**-0.9029**	**0.6305**	**0.3560**	**0.2808**	**1.2925**	
**LPE 20:0_1**	**-0.8193**	**0.1562**	**0.0557**	**-0.1615**	**1.0374**	**0.7817**	**0.6031**	**0.9052**	
**LPE 20:1**	**-0.2876**	**0.0389**	**0.0115**	**-0.3583**	**1.0304**	**0.7998**	**0.6281**	**0.8452**	
**LPE 20:3**	**-0.1778**	**0.3266**	**-0.1403**	**-0.2869**	**0.8271**	**0.7292**	**0.5368**	**0.7316**	
**LPE 20:4**	**-0.5683**	**0.0624**	**0.0192**	**-0.2451**	**0.9702**	**0.9159**	**0.5984**	**0.9105**	
**LPE 20:4_1**	**-0.5695**	**0.0613**	**0.0282**	**-0.2463**	**0.9694**	**0.9150**	**0.5980**	**0.9095**	
**LPE 22:5**	**-0.0304**	**0.4826**	**0.2699**	**0.1522**	**-1.4159**	**-0.5553**	**-1.1809**	**-0.5822**	
**LPE 22:5_2**	**0.2747**	**1.0184**	**0.8464**	**0.0435**	**-0.5263**	**0.2601**	**-0.2687**	**-0.2343**	
**PC 14:0_22:6**	**0.6851**	**0.8409**	**0.9073**	**0.7017**	**-0.6050**	**-0.9988**	**-1.2688**	**-0.8553**	**x**
**PC 15:0_22:5**	**-0.4750**	**-0.3929**	**-0.3458**	**-0.3937**	**0.6223**	**1.3075**	**1.1019**	**0.7316**	
**PC 16:0_18:1**	**-0.3015**	**-0.1488**	**-0.0011**	**-0.0676**	**0.5633**	**0.6589**	**0.5262**	**0.6209**	
**PC 16:0_18:1_1**	**-0.1934**	**-0.1647**	**0.1984**	**-0.2164**	**0.6206**	**0.8225**	**0.6092**	**0.8939**	
**PC 16:0_18:2**	**-0.5245**	**-0.2825**	**-0.1263**	**-0.4458**	**0.7396**	**0.8233**	**0.7240**	**0.8317**	

PC 16:0_20:3	0.3051	0.3177	0.4423	0.2773	-1.0952	-0.6560	-0.3053	-0.6383	
PC 16:0_20:4	0.2590	0.5360	0.5751	0.4187	-0.7914	-0.5251	-0.8605	-0.5820	
PC 16:0_22:4	0.0374	0.0409	0.0479	0.0398	0.6059	0.7981	0.8019	0.7987	
PC 16:0_22:5	0.3591	0.4949	0.4777	0.3404	-1.5974	-1.5479	-0.9390	-1.2528	
PC 16:0_22:6	0.7362	0.9134	0.8952	0.7254	-1.4225	-1.3720	-1.5219	-1.3011	x
PC 16:1_16:1	-0.3790	-0.1256	-0.4283	-0.1961	0.8838	1.0131	0.7889	0.8780	
PC 16:1_18:2	-0.4729	-0.3437	-0.0810	-0.4275	1.1247	1.2485	1.1297	1.2087	
PC 17:0_18:2	-0.1323	0.2653	0.1184	-0.6438	0.9487	1.0052	0.8438	1.2162	
PC 17:1_20:1	-0.9646	-0.9166	1.1753	-0.9170	1.3822	1.4525	1.3480	1.5271	
PC 17:1_22:6	-0.1699	0.1285	0.1237	-0.1037	0.9272	0.9627	0.9097	0.8844	x
PC 18:0_18:2	-0.4953	-0.2786	-0.1443	-0.3509	0.8216	0.8904	0.6917	0.9181	
PC 18:0_20:3	-0.5800	0.0351	-0.0034	-0.3098	0.5651	0.7509	0.2550	0.8121	
PC 18:0_20:3_1	-0.0187	0.9591	0.3372	0.4432	-1.0027	-0.9626	-1.0006	-0.9922	
PC 18:0_20:4	0.3502	0.4777	0.5024	0.5441	-0.9300	-0.7486	-1.0639	-0.7408	
PC 18:0_22:4	0.2058	0.4690	0.5302	0.3773	-1.0158	-0.5069	-0.8928	-0.9668	
PC 18:0_22:5	0.3188	0.4681	0.4888	0.3635	-1.4764	-1.4378	-1.7778	-0.8951	
PC 18:0_22:5_1	0.4399	0.7531	0.7777	0.5603	-0.8842	-0.6940	-1.0968	-0.7484	
PC 18:0_22:6	0.7337	0.8885	0.8905	0.7912	-1.4294	-1.5924	-1.5426	-1.3205	x
PC 18:1_18:2	-0.5596	-0.2402	-0.1280	-0.4145	0.8079	0.8809	0.8225	0.8499	
PC 18:1_20:1	0.9579	0.2760	0.8141	1.1949	-0.6053	0.0918	-0.5981	-0.4072	
PC 18:1_22:5	0.3934	0.2914	0.1155	-0.1044	-0.8563	-0.5633	-0.9514	-0.6564	
PC 18:2_18:2	-0.5910	-0.4219	-0.2919	-0.5252	0.9749	1.0552	0.9839	1.0929	
PC 18:2_18:3	-0.3658	-0.1648	-0.5615	-0.5297	0.5465	0.7014	0.5278	0.7904	
PC 18:2_20:4	-0.7538	-1.0585	-0.1311	0.0270	0.7594	1.0005	0.8305	0.7214	
PC 18:2_22:5	0.3245	0.8128	0.8559	0.6164	-0.4431	-0.1576	-0.4216	-0.2099	
PC 18:2_22:6	0.6201	0.8553	0.8826	0.6277	-1.3421	-1.2163	-1.2959	-1.2307	x
PC 19:0_18:2	-0.5286	-0.4058	-0.1998	-0.5432	0.6843	0.8416	0.6726	1.0163	
PC 19:0_20:3	-0.0484	0.3168	0.3925	0.3095	-0.3774	-0.4772	-0.5803	-0.4379	

PC 19:0_20:4	0.2028	0.4630	0.2646	0.3080	-0.6548	-0.6190	-0.9809	-0.3360	
PC 19:0_22:5	0.3029	0.3111	0.2516	0.2554	-1.0198	-1.0467	-1.8491	-1.0173	
PC 19:0_22:6	0.6298	0.6806	0.7205	0.6055	-2.1944	-0.1260	-1.4693	-0.7937	x
PC 20:1_22:6	0.4380	0.6933	0.5863	0.4890	-0.8126	-1.3068	-1.0425	-0.7334	x
PC 20:3_22:6	0.6007	0.6897	0.7464	0.4475	-0.9191	-0.7577	-1.1855	-0.9237	x
PC 22:4_22:6	0.6794	0.8738	0.8080	0.6351	-0.5952	-1.3619	-1.3940	-1.3869	x
PC 22:5_22:6	0.0995	0.3978	0.1849	0.2164	-0.9370	-0.8926	-0.9347	-0.9254	x
PC 22:6_22:6	0.3684	0.7815	0.7418	0.6349	-0.9282	-0.8842	-0.9260	-0.9167	x
PC 29:0	-0.3026	-0.4174	-0.4177	-0.1943	1.0924	1.0979	0.9759	1.2198	
PC 31:0	-0.5115	-0.4735	-0.4255	-0.4140	1.1952	1.1671	1.0671	1.3409	
PC 33:0	0.2290	0.2315	-0.5337	-0.3179	0.7977	0.7533	0.4920	1.1059	
PC 33:1	-0.3018	0.2814	0.0687	-0.0177	0.8332	0.8720	0.7372	0.8573	
PC 33:2	-0.4316	-0.3923	0.0124	-0.1540	0.7986	0.7617	0.5632	0.9137	
PC 34:2	0.3434	-0.2522	0.2337	-0.2601	0.8322	0.3999	0.9449	1.1552	
PC 34:3	-0.0923	-0.0546	-0.0840	0.0106	0.7161	0.6766	0.5743	1.0336	
PC 34:4	-0.8861	-0.2696	-0.1170	-0.2561	1.0500	1.1224	1.1538	1.3402	
PC 35:2	-0.1340	-0.3208	-0.1897	-0.4293	0.7787	0.7882	0.5686	1.0187	
PC 35:3	-0.2909	-0.0544	0.4787	-0.8956	0.5320	0.8235	0.8509	0.8811	
PC 35:4	0.1425	-0.0740	0.0574	-0.0515	0.5691	0.6249	0.5322	0.7021	
PC 37:6	0.6153	0.6695	0.5926	0.3283	-0.5139	-0.4657	-0.8736	-0.6235	
PC 38:4	-0.0737	0.3768	0.4421	0.1911	-0.5610	-0.3766	-0.7614	-0.2991	
PC 38:7	0.6812	0.8386	0.8965	0.6763	-1.3546	-0.4294	-1.0094	-1.0949	
PC 38:7_2	0.7559	0.7676	0.8626	0.5916	-0.8316	-0.1720	-1.6461	-1.6389	
PC 39:5	0.5998	0.6902	0.5205	0.4475	-1.2615	-0.6581	-1.9516	-0.7592	
PC 39:5_1	0.2122	0.3792	1.0813	0.4571	-0.2771	-0.3182	-0.3571	-0.3603	
PC 39:6	0.7547	0.7861	0.8891	0.7539	-0.8339	-0.9463	-1.5482	-0.8317	
PC 40:7	0.4908	0.6098	0.6468	0.4834	-1.5877	-1.0952	-1.1913	-1.0553	
PC 40:9	0.6620	0.6969	0.5319	0.5980	-0.7646	-0.9980	-1.8546	-0.9771	

PC 42:10	0.6187	0.8290	0.7857	0.7721	-1.6142	-0.8980	-1.6127	-1.6066	
PC 42:4	0.2405	0.3091	-0.0749	-0.0067	-0.0579	-1.7559	-0.7159	-0.8205	
PC 42:5_1	-0.3556	0.0335	0.4772	-0.2162	-1.0777	-0.3899	-0.7269	-0.9854	
PC 42:6	0.2748	0.2841	0.5121	0.4255	-0.7161	-0.6087	-1.2962	-0.3708	
PC 42:6_1	0.4770	0.5984	0.5589	0.3438	-1.3533	-0.8759	-1.4140	-1.0402	
PC 42:8_1	0.1728	0.7722	0.5890	0.5691	-1.8863	-0.7449	-1.0523	-0.3588	
PE 14:0_16:0	-0.4295	-0.1746	-0.1223	-0.2798	0.7715	0.3664	0.3388	0.5559	
PE 14:0_18:2	-0.3529	-0.1217	-0.1878	-0.5974	1.0321	1.0278	0.9787	0.9442	
PE 14:0_22:6	0.6550	0.7662	0.6748	0.5907	0.0673	-1.7120	-0.7317	-0.0089	x
PE 15:0_18:2	-0.8582	-0.7963	-0.7608	-0.0141	1.1769	1.1261	1.0898	1.3013	
PE 15:0_22:6	0.8384	0.7739	0.8096	0.7478	-1.4838	-1.4522	-1.4821	-0.7287	x
PE 16:0_16:1	-0.2703	-0.2457	-0.1058	-0.0166	0.9677	0.7019	0.7441	0.8790	
PE 16:0_18:1	-0.2555	-0.2651	-0.2450	-0.0708	1.1104	-0.0632	0.7810	0.6037	
PE 16:0_18:1_1	-0.1002	-0.1648	-0.4212	-0.1597	0.9707	1.0658	0.9120	0.9272	
PE 16:0_18:2	-0.5654	-0.3766	-0.3569	-0.5494	1.1451	0.9632	0.9821	1.0460	
PE 16:0_20:3	0.0353	-0.0241	-0.0497	0.2084	0.7331	0.4295	0.4244	0.6487	
PE 16:0_20:4	-0.2939	-0.0662	-0.0727	-0.2315	0.8178	0.6601	0.5904	0.7643	
PE 16:0_22:5	0.1152	0.3586	0.3049	0.0952	-0.9523	-0.9090	-0.9500	-0.9409	
PE 16:0_22:6	0.5769	0.7743	0.7012	0.5630	-1.1358	-1.1447	-1.3054	-1.0450	x
PE 16:1_18:2	-0.2113	-0.4165	-0.3178	-0.2113	1.2228	1.0961	1.0603	1.1455	
PE 16:1_20:4	-0.2611	-0.2089	-0.2690	-0.0957	1.1082	0.9906	0.8177	1.1170	
PE 16:1_22:6	0.5201	0.7730	0.7816	0.6493	-0.4522	-0.6791	-0.9301	-0.6561	x
PE 16:2_18:2	-0.4565	-0.3674	-0.3163	-0.3682	0.9960	0.7819	0.8179	0.9575	
PE 17:0_18:2	-0.5968	-0.4558	-0.3597	-0.4386	1.2072	1.0749	1.0441	1.1509	
PE 17:0_20:4	-0.1052	0.2247	0.1011	-0.0642	0.8977	0.7356	0.5501	0.8917	
PE 17:0_22:5	0.1731	0.3838	0.0107	0.3643	-0.5998	-0.5654	-0.6010	-0.7157	
PE 17:0_22:6	0.7930	0.8721	0.8417	0.6563	-1.5909	-1.5615	-1.5894	-0.8866	x
PE 17:1_18:2	0.1101	-0.5223	-0.4064	-0.4859	1.0603	0.9430	0.7649	1.0382	

PE 17:1_22:6	0.6842	0.7932	0.7679	0.6975	-0.7429	-0.7425	-0.6979	-0.5099	x
PE 18:0_18:1	0.0653	-0.2972	-0.1894	0.3478	0.6003	0.2965	0.4782	0.4671	
PE 18:0_18:1_1	-0.4078	-0.1682	-0.0854	-0.3438	0.7937	0.6601	0.6592	0.7142	
PE 18:0_18:2	-0.4844	-0.3104	-0.3396	-0.5264	1.1870	0.8879	0.8989	1.0164	
PE 18:0_18:2_1	-0.4952	-0.2341	-0.1320	-0.5501	0.7216	0.7715	0.5770	0.7193	
PE 18:0_18:2_2	-0.7995	-0.7564	-0.7316	-0.7568	2.8648	2.7056	-0.0409	-0.0430	
PE 18:0_20:3	-0.1017	-0.2033	-0.4522	-0.0618	0.5829	0.7303	0.5296	0.9129	
PE 18:0_20:3_1	-0.4617	-0.2384	-0.4473	-0.3870	1.2446	0.9573	0.7453	0.9699	
PE 18:0_20:4	-0.1784	0.0555	-0.0188	-0.1152	0.8518	0.7019	0.4921	0.7651	
PE 18:0_20:4_1	0.0958	0.3794	0.3740	0.2005	-0.4777	-0.3806	-0.2644	-0.7700	
PE 18:0_20:4_3	-0.1784	0.0554	-0.0189	-0.1152	0.8516	0.7029	0.4919	0.7650	
PE 18:0_22:5	0.1847	0.3455	0.2894	0.1516	-1.0329	-1.0745	-1.1270	-0.9572	
PE 18:0_22:5_1	0.7676	0.7983	0.8622	0.7489	-0.4461	-0.9064	-0.2025	-1.7619	
PE 18:0_22:6	0.5908	0.7629	0.7195	0.6132	-0.9750	-1.1011	-1.2057	-1.0454	x
PE 18:0_22:6_1	0.3750	0.7196	0.7630	0.3230	-0.1348	-0.7163	-0.6121	-0.4134	x
PE 18:0_22:6_3	0.3514	0.6936	0.7367	0.2997	-0.1372	-0.7323	-0.6288	-0.4315	x
PE 18:1_18:2	-0.5215	-0.4288	-0.3972	-0.5170	1.2680	1.1179	1.0914	1.1684	
PE 18:1_18:2_1	-0.2520	-0.6632	-0.0870	-0.2691	1.0531	1.0704	0.9530	0.9972	
PE 18:1_20:3	-0.1262	0.1789	-0.0808	0.0289	0.7661	0.4980	0.4283	0.6547	
PE 18:1_20:4	-0.3556	-0.1600	-0.1288	-0.3537	1.0596	0.9877	0.8702	1.0212	
PE 18:1_22:5	0.0181	0.3366	0.2991	0.0599	-0.4340	-0.8397	-0.9816	-0.5814	
PE 18:1_22:6	0.5543	0.6577	0.6516	0.5789	-0.8795	-0.9391	-1.1500	-0.8991	x
PE 18:1_22:6_2	0.5541	0.6571	0.6515	0.5788	-0.8796	-0.9392	-1.1501	-0.8992	x
PE 18:2_18:2	-0.4706	-0.2821	-0.2276	-0.4944	1.1201	0.9299	0.9117	1.0646	
PE 18:2_18:2_1	-0.4597	-0.3713	-0.3207	-0.3722	0.8470	0.7672	0.9908	0.9815	
PE 18:2_18:3	-0.6093	-0.4297	-0.3863	-0.7913	1.1437	1.0314	0.9359	1.0878	
PE 18:2_20:2	-0.5714	-0.3723	-0.1394	-0.5445	0.8142	0.8752	0.8833	0.8659	
PE 18:2_20:4	-0.1745	-0.0009	0.0295	-0.0427	0.8168	0.7651	0.5155	0.8418	

PE 18:2_22:5	0.4224	0.8856	0.7810	0.4226	-0.5348	-0.1976	-0.4051	-0.0973	
PE 18:2_22:6	0.6721	0.8229	0.7499	0.6228	-0.8847	-0.9521	-1.0916	-1.0613	x
PE 18:3_20:4	-0.1969	-0.0330	-0.1195	-0.2131	1.2207	1.0731	0.9272	0.6433	
PE 18:3_22:6	0.5732	0.8605	0.7756	0.6854	-1.4957	-1.4640	-0.7434	-1.4874	x
PE 19:0_18:2	-0.4272	-0.2630	-0.4353	-0.9170	0.9417	0.7908	0.7849	0.9844	
PE 19:0_18:2_1	-0.1690	-0.1282	-0.3807	-0.3778	0.6025	0.8784	0.8062	0.9229	
PE 19:0_20:3	-0.4573	-0.3986	-0.1513	-0.3006	0.5878	0.6029	0.1442	0.3802	
PE 19:0_20:4	-0.4678	-0.3348	-0.2971	-0.6640	0.7017	0.7911	0.6105	0.9652	
PE 19:0_22:6	0.5619	0.6772	0.3648	0.4521	-0.9418	-0.8974	-0.9395	-0.9302	x
PE 19:1_18:2	-0.0660	-0.2873	-0.1319	-0.2073	0.5748	0.4422	0.4059	0.6841	
PE 20:0_18:2	-0.5447	-0.3416	0.0694	-0.4579	0.8223	0.5837	0.4761	0.6006	
PE 20:0_18:2_1	-1.1715	-1.1189	-1.0889	-1.1194	1.3215	0.8390	1.0697	1.1847	
PE 20:0_20:4	-0.5577	-0.1310	-0.2111	-0.4300	0.8088	0.6785	0.6069	0.9451	
PE 20:0_20:4_1	0.0863	0.5690	0.3783	0.2509	-0.4548	-0.6521	-0.8397	-0.4718	
PE 20:0_22:5	0.1790	0.4574	0.5792	0.3196	-1.7575	-1.0081	-0.7783	-1.7496	
PE 20:0_22:5_1	0.4115	0.5619	0.5565	0.4693	-0.8205	-0.9439	-1.3666	-0.6986	
PE 20:0_22:6	0.4835	0.7220	0.6841	0.5067	-0.6143	-0.7221	-0.8392	-0.8649	x
PE 20:0_22:6_1	0.4369	0.9271	0.6191	0.2884	-1.0706	-0.6621	-1.3926	-0.8271	x
PE 20:1_20:4	0.2477	0.5408	0.5070	0.8746	-1.3358	-0.3307	-0.6445	-1.0174	
PE 20:1_22:5	-0.0660	0.2381	0.0726	-0.1039	-0.2139	-0.2173	-0.7510	-0.4995	
PE 20:1_22:6	0.5079	0.7128	0.6047	0.4798	-0.3401	-1.8182	-0.6765	-0.1545	x
PE 20:2_22:6	0.1000	0.3430	0.6392	0.5725	-0.7312	-1.2852	-0.1740	-1.0081	x
PE 20:2_22:6_1	0.5173	0.7935	0.7738	0.4882	-1.0111	-1.6833	-0.7777	-0.9383	x
PE 20:3_22:6	0.6611	0.7735	0.7260	0.6593	-1.7745	-0.3069	-0.3732	-1.3467	x
PE 20:4_22:6	0.5775	0.9167	0.7721	0.6812	-0.6313	-1.3882	-1.4198	-1.4128	x
PE 21:0_18:2	0.0924	-0.6723	-0.6273	0.2064	0.8528	0.3899	0.8951	0.5057	
PE 21:0_20:4_1	-0.7048	-0.6359	-0.5965	-0.6366	0.3569	1.4200	0.6624	1.1130	
PE 22:4_22:6	0.6422	0.8179	0.8168	0.2130	-0.9971	-0.3427	-0.7841	-1.0473	x

PE 22:5_22:6	0.6750	0.7278	0.8028	0.5590	-1.2046	-1.2005	-1.2563	-1.1930	x
PE 22:6_22:6	0.7344	0.8799	0.9322	0.9375	-1.0238	-0.9862	-1.0219	-1.0140	x
PE 24:0_18:2	-0.4426	-0.3148	-0.4659	-1.0079	0.2597	0.2699	0.3111	0.3526	
PE 32:0	-0.2036	-0.2286	0.0732	-0.4604	0.2990	0.6746	0.7040	0.6469	
PE 38:1	-0.1862	-0.6149	-0.0552	-0.0224	0.5359	0.6339	0.5673	0.3959	
PE 38:3	-0.6670	-0.3968	-0.0341	-0.3868	0.5553	0.8871	0.8554	0.9125	
PE 38:7	-0.5288	-0.0282	-0.2286	-0.5643	0.5370	0.6994	0.5148	0.6947	
PE 39:7	0.5097	0.6532	0.6535	0.5493	-0.9096	-0.8678	-0.6306	-0.7208	
PE 40:9	0.3505	0.5716	0.5899	0.4832	-0.8332	-0.6066	-0.9688	-0.6461	
PE 40:9_1	0.1479	0.3682	0.0526	0.3595	-0.3937	-0.6176	-0.9158	-0.5411	
PE 42:10	0.4127	0.7490	0.7924	0.5715	-0.7997	-0.7110	-0.9738	-0.7239	
PE 42:10_1	0.6281	0.8470	0.8733	0.6321	-1.3431	-1.2163	-1.3230	-1.2118	
PE 42:9	0.4452	0.5463	0.5626	0.5567	-0.9976	-0.7168	-0.9879	-0.8362	
PG 16:0_16:0	-0.2325	0.1731	0.1624	-0.1580	0.9714	0.3020	0.3795	0.7584	
PG 16:0_18:2	-0.3191	0.3223	-0.0707	-0.1717	0.5192	0.6257	0.0734	0.9356	
PG 16:0_22:6	0.6673	0.7621	0.5669	0.5908	-0.4729	-0.4978	-1.4591	-1.4510	x
PG 16:1_18:2	-0.0913	0.3806	-0.1571	-0.2658	1.1194	1.0229	0.9405	1.0834	
PG 16:1_18:2_1	0.0489	0.0757	-0.0542	0.1213	1.0784	0.9591	0.9730	1.0591	
PG 16:2_18:2	-0.4572	-0.3688	-0.3181	-0.3696	0.9897	0.7829	0.7270	1.0641	
PG 18:1_18:2	-0.1748	-0.1117	-0.1854	-0.1381	0.8612	0.7797	0.6980	0.8807	
PG 18:1_20:4	0.5341	0.6347	0.4675	0.5742	0.1578	-0.1774	-0.5814	0.0423	
PG 18:1_20:4_1	0.4098	0.8650	0.9493	0.3588	-0.4014	-0.4163	-0.4683	-0.5270	
PG 18:1_22:6	-0.2716	0.1507	0.5586	0.3470	-0.8426	-1.0522	-1.8980	-0.5334	x
PG 18:2_18:2_1	-0.1538	-0.1118	-0.0789	-0.1495	0.7115	0.7439	0.5604	0.8127	
PG 18:2_20:4	0.4847	0.6147	0.6751	0.2279	-0.4463	-0.4993	-0.5985	-0.2170	
PG 18:2_22:6	0.5483	0.6382	0.5752	0.6265	-0.9269	-0.8821	-0.9246	-0.9152	x
PG 20:4_22:5	-0.2635	0.3162	0.3418	0.3519	-1.0865	-1.0497	-1.0846	-1.0769	
PG 20:4_22:6	0.5886	0.7529	0.6074	0.6473	-0.9636	-0.9213	-0.9614	-0.9526	x

PG 22:4_22:5	0.2510	0.1745	0.0643	0.2013	-0.9754	-0.9343	-0.9733	-0.9647	
PG 22:5_22:5	0.1170	-0.1691	-0.1695	-0.0554	-1.0536	-1.0186	-1.0518	-1.0445	
PG 22:5_22:6	0.2447	0.1483	0.1910	0.3934	-0.9283	-0.8840	-0.9260	-0.9167	x
PI 16:0_18:2	0.2133	-0.2829	-0.3381	0.0321	0.9606	0.4961	0.4531	0.8630	
PI 18:0_18:1	-0.0532	-0.2308	-1.2410	0.1328	1.3039	0.7691	0.8724	0.8254	
PI 18:0_18:2	-0.0145	-0.2805	-0.3105	-0.1618	1.1742	0.4608	0.5588	0.9871	
PI 18:0_20:3	0.0663	-0.1804	-0.1631	-0.0843	1.1149	0.4125	0.4156	0.9553	
PI 18:0_22:5	0.2966	0.0352	0.0461	0.3173	-0.6453	-0.9992	-1.1044	-0.7144	
PI 18:0_22:5_1	0.9063	0.5956	0.4074	0.7387	-0.1936	-0.6999	-0.6649	-0.3865	
PI 18:0_22:6	0.9856	0.6712	0.5951	0.8276	-0.9989	-1.1085	-1.1514	-0.9544	x
PI 18:1_18:2	0.2673	-0.0701	-0.2404	0.3928	1.2269	0.3652	0.0546	0.2526	
PMeOH 16:0_18:2	0.1670	0.2759	-0.0369	-0.1880	0.6176	0.5080	0.3476	0.7153	
PS 18:1_20:4	0.6711	0.7619	0.7866	0.6713	0.4232	-0.5085	-1.0669	0.1766	
SM d36:0	-0.5420	-0.4569	-0.4081	1.0132	0.6332	0.8982	0.5977	0.7475	
SM d36:1	-0.3976	-0.3139	-0.2659	-0.3147	1.1420	0.7663	1.4732	-0.3249	
SM d36:2	-0.2582	-0.2459	-0.0207	0.1617	0.8988	0.6811	0.3479	0.6277	
SM d38:2	-0.3085	-0.1592	-0.0127	-0.0714	0.5508	0.6564	0.5067	0.6195	
SM d42:3	-0.1396	-0.3144	-0.1498	-0.0805	0.3552	0.5429	0.2665	0.5659	
TG 18:2_18:2_18:3	0.8644	0.2891	0.3691	0.7946	-0.1241	-0.0170	-0.0450	0.0787	
TG 54:6	1.1179	0.5833	0.1241	0.6083	-1.6421	-0.7986	-0.0061	-1.6332	
TG 54:7	0.8674	0.2927	0.3711	0.7976	-0.1201	-0.0054	-0.0411	0.0523	
TG 56:6	1.5009	0.4102	0.2407	1.3263	-0.6878	-1.4144	0.4933	-0.4267	
TG 56:7	1.5728	0.4514	0.2667	1.3017	-0.8052	-0.4768	-0.5827	-0.7428	
TG 56:8	1.5667	0.5101	-0.1861	1.3887	-0.9802	-0.7140	0.1391	-1.7419	
TG 58:10	0.7112	0.5527	0.4935	0.5498	-0.1663	-0.0461	-0.2949	0.0428	
TG 58:7	1.4181	0.6929	0.6490	1.1345	-1.5391	-0.7545	-0.5597	-0.5088	
TG 60:10	1.2130	0.7041	0.4635	0.8076	-1.5661	-0.7620	-1.5645	-0.6097	
TG 62:11	1.4068	0.5914	-0.0960	1.1849	-1.0561	-1.0218	-1.0543	-1.0471	

Red denotes upregulation, blue denotes downregulation.

Supplementary Table 9: Significantly-Altered Lipids in *Tmem135*$^{FUN025/FUN025}$ (*FUN025*) Plasmas Compared to WT.

Lipid Species	Log10 Lipid Concentrations							Contains DHA
	WT 1	WT 2	WT 3	WT 4	*FUN025* 1	*FUN025* 2	*FUN025* 3	
ACar 14:0	3.6746	3.8951	3.6231	3.6502	3.9707	4.0346	4.0298	
ACar 16:1	3.7186	4.0175	3.6711	3.6500	4.1158	4.1444	4.1732	
ACar 18:1	4.4540	4.5951	4.2476	4.2888	4.6184	4.6850	4.6510	
CE 16:1	5.4822	5.4028	5.4981	5.3387	5.6588	5.6601	5.5911	
CE 18:1	6.0255	6.0421	6.0759	5.9298	6.2972	6.3163	6.2954	
CE 18:3	5.3687	5.3834	5.3816	5.6616	5.7845	5.7528	5.7727	
CE 20:3	5.8976	5.8355	5.8043	5.8756	6.1101	6.0605	6.1179	
CE 20:4	7.1060	7.1589	7.0898	7.1014	6.7627	6.7526	6.7662	
CE 22:5	5.2291	5.2082	5.2820	4.9785	4.4017	4.3976	4.6113	
CE 22:6	6.4584	6.5110	6.4166	6.5356	5.1825	5.1631	5.1842	x
Cer_ADS d39:0	4.2734	4.3091	4.3155	4.2519	3.0664	3.7654	3.0664	
Cer_ADS d40:0	5.4088	5.3957	5.3860	5.6277	5.2106	5.1162	5.1606	
Cer_AS d18:1_23:0	4.1443	4.1183	4.2298	4.2012	3.2006	3.8995	3.2006	
Cer_BDS d22:0_16:0	4.0827	4.3099	4.1632	4.3483	3.9333	3.8555	3.9170	
Cer_BDS d41:0	4.9186	4.8722	4.8443	4.9623	4.5714	4.5830	4.5968	
Cer_NDS d18:0_16:0	4.1828	4.1810	4.3565	4.1114	3.2726	3.2726	3.2726	
Cer_NDS d18:0_16:0_1	4.1828	4.1810	4.3565	4.1114	3.2878	3.2878	3.2878	
Cer_NP t18:0_16:0	4.6689	4.7109	4.7867	4.6693	4.4506	4.4746	4.4350	
Cer_NP t18:0_16:0_1	4.6700	4.7109	4.7867	4.6693	4.4506	4.4746	4.4350	
Cer_NP t18:0_22:0	5.4088	5.3957	5.3860	5.6277	5.2106	5.1162	5.1606	
Cer_NP t18:0_23:0	4.9186	4.8722	4.8443	4.9623	4.5714	4.5830	4.5968	
EtherPE 14:1e_20:4	4.7502	4.7165	4.7541	4.5643	3.9260	3.8268	3.9740	
EtherPE 16:0e_18:2	5.6412	5.6123	5.5554	5.6876	5.7932	5.7560	5.8263	
EtherPE 16:0e_22:6	6.1717	6.2094	6.0416	6.1235	5.0883	3.9062	4.7126	x
EtherPE 16:1e_18:1	5.2599	5.1620	5.1928	5.2703	5.3359	5.3192	5.3640	

EtherPE 16:1e_18:2	6.0077	5.9656	5.9345	6.0725	6.1214	6.1176	6.1627	
EtherPE 16:1e_20:4	6.1758	6.2286	6.1565	6.2141	5.7485	5.7864	5.8518	
EtherPE 16:1e_22:5	5.8039	5.8010	5.7903	5.7354	5.2356	5.1744	5.3286	
EtherPE 16:1e_22:6	6.4351	6.4406	6.3791	6.3871	5.4206	5.4493	5.4868	x
EtherPE 16:2e_22:6	5.0891	5.1241	5.0205	4.9000	4.3779	4.3437	4.4509	x
EtherPE 18:0e_20:4	5.9114	5.9397	5.8684	6.0701	5.5841	5.5515	5.5229	
EtherPE 18:0e_22:4	5.5471	5.5899	5.6664	5.7387	5.3177	5.2416	5.2577	
EtherPE 18:0e_22:5	5.2085	5.3200	5.2196	5.5023	4.7333	4.7724	4.5468	
EtherPE 18:0e_22:6	6.2832	6.3271	6.1795	6.3493	5.0382	4.4823	5.0764	x
EtherPE 18:1e_20:4	6.0152	5.6463	5.9595	6.1160	5.5870	5.3287	5.5836	
EtherPE 18:1e_22:6	6.1907	6.2613	6.1607	6.3124	4.9002	4.8729	4.9295	x
EtherPE 18:2e_20:4	5.7479	5.7160	5.7241	5.7643	5.4751	5.3075	5.5951	
EtherPE 18:2e_22:6	5.7078	5.7004	5.6771	5.6571	4.6282	4.6353	4.7103	x
FA 18:1_1	4.7528	4.5600	4.8062	4.7012	4.8678	4.9068	4.9053	
FA 22:4	4.9204	5.0348	5.1037	5.0347	4.5979	4.6734	4.6018	
HexCer_NDS d21:0_18:1	4.7650	4.8641	4.6627	4.8191	5.0383	4.9887	4.9909	
HexCer_NDS d40:1	5.6304	5.7773	5.5708	5.8099	5.9287	5.9225	5.9331	
HexCer_NDS d40:2	5.3008	5.3396	5.2392	5.3146	5.4755	5.4699	5.4562	
HexCer_NDS d41:2	4.8197	4.9289	4.8099	4.8252	5.1027	5.1333	5.0774	
HexCer_NDS d42:2	5.7022	5.7310	5.7015	5.7213	5.9610	5.9870	5.9721	
HexCer_NS d18:1_16:0	4.4513	4.4504	4.3733	4.4327	4.5927	4.6311	4.5389	
HexCer_NS d18:1_24:1	4.8837	4.8168	4.8615	4.8247	5.0777	5.1556	5.1140	
HexCer_NS d36:1	4.3393	4.4350	4.3712	4.3462	4.7334	4.7487	4.6908	
HexCer_NS d38:1	4.7916	4.9352	4.7539	4.8758	5.1293	5.0940	5.0842	
HexCer_NS d38:1_1	4.7916	4.9352	4.7539	4.8758	5.1293	5.0940	5.0842	
HexCer_NS d40:1	5.6081	5.7773	5.5708	5.8079	5.9287	5.9225	5.9331	
HexCer_NS d42:1	5.3564	5.4556	5.4049	5.3927	5.6536	5.6569	5.5766	
HexCer_NS d42:2	5.7022	5.7353	5.7015	5.7224	5.9610	5.9870	5.9821	

**LPC 0:0/18:0**	5.3775	5.4158	5.4190	5.4670	5.3273	5.3444	5.3354	
**LPC 0:0/18:1**	5.3236	5.2630	5.3731	5.3310	5.5117	5.5173	5.5195	
**LPC 0:0/20:4**	5.1628	5.1636	5.2654	5.2368	4.7852	4.7783	4.7887	
**LPC 0:0/22:2**	3.7758	3.6194	3.7475	3.9595	2.9205	2.9205	2.9205	
**LPC 0:0/22:5**	3.8583	3.8910	4.1200	3.9030	3.1222	2.4802	3.3765	
**LPC 16:1/0:0**	5.2296	5.1273	5.1857	5.1373	5.3429	5.3872	5.3614	
**LPC 17:0/0:0**	4.8652	4.8236	4.8380	4.8290	4.4987	4.4370	4.4781	
**LPC 18:0/0:0**	6.5382	6.5727	6.5465	6.6577	6.4846	6.4833	6.4941	
**LPC 18:1/0:0**	6.3043	6.2116	6.3051	6.2242	6.3976	6.4247	6.4195	
**LPC 19:0/0:0**	5.0281	4.9704	4.9483	4.9013	4.6810	4.6075	4.7545	
**LPC 20:0/0:0**	5.1793	5.1961	5.1031	5.2786	4.9929	4.9492	5.0209	
**LPC 20:0/0:0_1**	4.2259	4.2393	4.3258	4.0803	3.9950	3.5807	3.9582	
**LPC 20:1/0:0**	5.2100	5.0713	5.1690	5.0455	4.9150	4.9474	4.9979	
**LPC 20:3/0:0**	5.4025	5.3040	5.4680	5.4261	5.5360	5.5729	5.5672	
**LPC 20:4/0:0**	6.0238	5.9671	6.0590	5.9984	5.5879	5.5715	5.5480	
**LPC 22:0/0:0**	4.5147	4.5055	4.5215	4.5610	4.3770	4.3725	4.3177	
**LPC 22:1/0:0**	4.4184	4.2796	4.4492	4.2505	3.9500	4.0169	4.1142	
**LPC 22:4/0:0**	4.2106	4.0253	4.3033	4.1807	3.8766	3.9071	3.9513	
**LPC 22:5/0:0**	4.5444	4.5086	4.7151	4.3366	3.7465	3.6866	3.6416	
**LPC 22:5/0:0_1**	4.4237	4.2808	4.5145	4.6194	4.0672	4.0335	3.8546	
**LPC 22:6/0:0**	5.5969	5.5742	5.6743	5.7032	4.1758	4.2852	4.1791	x
**LPC 23:0/0:0**	4.0131	4.0125	4.0142	3.9579	3.8837	3.7075	3.8686	
**LPC 24:0/0:0**	4.6412	4.6476	4.6613	4.6955	4.5925	4.5498	4.5759	
**LPE 18:0**	4.0479	4.0537	4.1000	4.1948	3.8151	3.9681	3.8803	
**LPE 18:1**	5.5777	5.4165	5.6364	5.5467	5.7805	5.8194	5.7252	
**LPE 18:2**	5.8945	5.7946	6.0519	6.1178	6.3650	6.3595	6.2427	
**LPE 18:2**	4.0430	3.8635	4.1042	4.2025	4.3551	4.3554	4.2424	
**LPE 20:0**	4.6195	4.6572	4.5649	4.7636	4.2069	4.3265	4.4137	

LPE 20:3	4.9567	4.8120	5.1305	4.9459	5.5351	5.5676	5.3512	
LPE 22:6	6.1852	6.1934	6.3736	6.3461	5.1892	5.2128	5.0334	x
PC 15:0_18:2	5.5652	5.4409	5.5297	5.5891	5.3052	5.2649	5.3146	
PC 15:0_20:4	5.3581	5.2442	5.3173	5.3477	4.9871	5.0164	5.0764	
PC 15:0_22:6	5.2378	5.2572	5.2049	5.3155	4.8920	4.9295	4.9367	x
PC 16:0_16:0	6.3608	6.2978	6.2542	6.3127	6.0867	6.0418	6.0621	
PC 16:0_18:0	5.6925	5.6578	5.5749	5.7679	5.2963	5.3476	5.3991	
PC 16:0_18:1	7.5071	7.4792	7.5572	7.5314	7.7690	7.7746	7.7737	
PC 16:0_18:3	2.6254	2.6254	2.6254	3.3240	3.7993	4.0530	4.1577	
PC 16:0_20:3	6.7783	6.7468	6.8324	6.8876	6.9812	6.9931	7.0254	
PC 16:0_20:4	7.5196	7.5115	7.5270	7.5267	6.9622	6.9271	6.9683	
PC 16:0_22:5	6.1040	6.0855	6.1665	6.1616	5.3963	5.4142	5.4840	
PC 16:0_22:6	7.1701	7.1961	7.1220	7.3050	5.6126	5.5824	5.6406	x
PC 16:1_22:6	5.5084	5.5218	5.4999	5.5793	4.3964	3.9666	3.2047	x
PC 17:0_18:2	6.0064	5.9619	5.9894	6.0899	5.7061	5.6449	5.7523	
PC 17:1_20:5	5.5819	5.5575	5.5670	5.7241	3.5279	4.4851	5.0583	
PC 18:0_18:1	6.6118	6.6111	6.6718	6.6744	6.8763	6.8877	6.8998	
PC 18:0_20:2	6.0498	6.0000	5.5316	6.1677	6.0447	6.0071	6.0826	
PC 18:0_20:3	6.4576	6.4488	6.4750	6.5582	6.6619	6.6642	6.7018	
PC 18:0_20:4	7.2582	7.3281	7.2986	7.3128	6.8029	6.7646	6.8118	
PC 18:0_22:6	6.5252	6.6308	6.5558	6.7204	5.1113	5.0344	5.1385	x
PC 18:1_20:3_1	5.3419	5.2444	5.3404	5.3394	5.5480	5.5742	5.5681	
PC 18:1_20:4	6.9095	6.8343	6.8918	6.7730	6.3274	6.3017	6.3535	
PC 18:1_22:6	6.3382	6.2910	6.2636	6.2507	4.7755	4.7529	4.8227	x
PC 18:2_20:4	6.2479	6.1512	6.0970	6.2425	5.8283	5.7687	5.8247	
PC 18:2_22:4	5.5118	5.3170	5.4226	5.4209	4.2539	5.0188	4.7387	
PC 18:2_22:6	5.6902	5.6571	5.6570	5.6720	4.6577	4.6083	4.7105	x
PC 18:3_20:4	4.3846	4.5250	4.6603	5.0256	4.2666	3.5676	3.5676	

**PC 19:0_18:1**	**5.0712**	**5.0469**	**5.1921**	**4.8629**	**4.8141**	**4.8671**	**4.5546**	
**PC 19:0_18:2**	**5.9821**	**5.9376**	**5.9745**	**5.9434**	**5.7107**	**5.6321**	**5.7654**	
**PC 19:0_22:6**	**4.8582**	**4.9142**	**4.8803**	**4.9452**	**4.1537**	**4.1537**	**4.1537**	x
**PC 20:0_20:4**	**4.4827**	**4.6097**	**4.6560**	**4.6243**	**4.7640**	**4.8471**	**4.7979**	
**PC 20:0_22:6**	**4.9074**	**4.9655**	**5.0204**	**5.2114**	**3.9614**	**3.9614**	**3.9614**	x
**PC 20:1_20:4**	**5.9328**	**5.9469**	**5.9936**	**5.9503**	**5.4325**	**5.4435**	**5.4742**	
**PC 20:3_22:6**	**4.3324**	**4.2591**	**4.2338**	**4.3383**	**4.0237**	**4.0516**	**4.0299**	x
**PC 20:4_22:5**	**4.7440**	**4.3313**	**4.2678**	**4.6546**	**3.7921**	**3.0932**	**3.0932**	
**PC 22:6_22:6**	**4.0787**	**4.0631**	**4.1842**	**4.1513**	**3.3063**	**3.3063**	**3.3063**	x
**PC 34:4**	**4.9496**	**4.6831**	**4.7355**	**4.8692**	**4.5894**	**4.3582**	**4.1026**	
**PC 36:5**	**5.8685**	**5.7611**	**5.7936**	**5.6967**	**5.2919**	**5.3756**	**5.4080**	
**PC 36:5_1**	**5.5909**	**5.4997**	**5.5813**	**6.1254**	**5.2892**	**5.1995**	**5.1411**	
**PC 36:6**	**4.6115**	**4.5131**	**4.5590**	**4.6698**	**3.7719**	**3.7719**	**3.7719**	
**PC 37:3**	**5.1716**	**4.9706**	**5.1544**	**4.8920**	**4.6413**	**4.5679**	**4.8829**	
**PC 37:4**	**5.2299**	**5.1641**	**5.2290**	**4.9081**	**3.9921**	**4.5211**	**4.5899**	
**PC 38:7**	**4.5137**	**4.6367**	**4.8468**	**4.8520**	**3.8147**	**3.8147**	**3.8147**	
**PC 39:4**	**5.3820**	**5.4146**	**5.4218**	**5.2755**	**4.6282**	**4.4042**	**4.4587**	
**PC 39:4_1**	**5.0071**	**4.9341**	**4.8743**	**4.3518**	**3.9051**	**3.2062**	**3.2062**	
**PC 39:5**	**4.8392**	**4.8036**	**4.7723**	**4.6777**	**4.5188**	**4.4422**	**4.5227**	
**PC 39:6**	**5.2266**	**5.1855**	**5.1976**	**5.1969**	**4.3536**	**4.3536**	**4.3536**	
**PC 39:6_1**	**5.0497**	**5.0484**	**4.9884**	**5.1212**	**3.8987**	**3.9853**	**3.7186**	
**PC 39:7**	**4.6940**	**4.5290**	**4.5826**	**4.5448**	**3.3344**	**3.3344**	**3.3344**	
**PC 40:6**	**5.5161**	**4.9368**	**5.5171**	**5.3910**	**4.2076**	**3.1556**	**4.9416**	
**PC 40:9**	**4.3055**	**4.2140**	**4.0627**	**4.5530**	**2.7099**	**2.7099**	**2.7099**	
**PC 41:7**	**4.1704**	**4.0615**	**4.2621**	**3.3625**	**3.3625**	**3.3625**	**3.3625**	
**PC 42:1**	**4.0005**	**4.2057**	**4.3282**	**4.1348**	**3.8291**	**3.5495**	**3.7997**	
**PC 42:10**	**5.1939**	**5.1859**	**5.1763**	**5.1112**	**4.2810**	**4.2810**	**4.2810**	
**PC 42:5**	**4.7728**	**4.4459**	**4.6948**	**4.4139**	**3.3565**	**4.0555**	**3.3565**	

PC 42:5_1	4.2817	3.8861	3.7044	3.9420	3.0055	3.0055	3.0055	
PC 42:6	4.4822	4.3880	4.5618	4.4916	4.0677	4.0843	4.1741	
PC 42:7	5.1805	5.1485	5.1615	5.0939	4.3949	4.3949	4.3949	
PC 44:7	4.2011	3.7866	4.1188	3.8123	3.0877	3.0877	3.0877	
PE 15:0_22:6	4.9373	4.9015	4.8305	4.7972	3.0958	3.7948	3.8790	x
PE 16:0_16:1	4.5575	4.4949	4.0288	4.4720	4.8598	5.0520	5.0905	
PE 16:0_18:0	4.9920	4.9405	4.8823	4.9224	4.7470	4.7442	4.6660	
PE 16:0_18:2	6.3479	6.2874	6.3038	6.3799	6.6562	6.6827	6.7011	
PE 16:0_20:4	6.3159	6.3592	6.2617	6.2959	6.4054	6.6216	6.7024	
PE 16:0_22:5	5.5171	5.4666	5.4829	5.5049	4.9424	4.9551	4.9759	
PE 16:0_22:6	6.7790	6.7616	6.6816	6.8166	5.4103	5.4495	5.4712	x
PE 16:1_18:2	5.6664	5.5752	5.5007	5.6109	5.9503	6.0234	6.0476	x
PE 16:1_22:6	5.0037	4.9419	4.7639	5.0671	3.2174	3.9164	4.1387	
PE 17:0_18:2	5.2447	5.2291	5.2700	5.3479	4.9767	5.0176	5.0715	x
PE 17:0_20:4	4.9284	4.9236	4.8963	4.8844	4.3985	4.4285	4.3822	
PE 17:1_22:6	5.1576	5.0810	4.9787	5.0311	4.0682	4.0852	4.0456	
PE 18:0_16:1	4.8952	4.8437	4.9095	4.8921	5.1661	5.1495	5.1034	x
PE 18:0_18:1	6.0462	6.0388	6.0856	6.0454	6.2761	6.2912	6.2728	
PE 18:0_20:4	6.8106	6.8589	6.8018	6.9170	6.6571	6.6340	6.6856	
PE 18:0_20:4_1	6.0832	6.0996	6.1048	6.1125	5.7860	5.7487	5.7720	
PE 18:0_22:5	5.0864	5.1174	5.0702	4.8110	4.3289	4.2814	4.2995	
PE 18:0_22:6	6.3423	6.4237	6.2953	6.4854	4.9331	4.8898	4.9170	
PE 18:0_22:6_1	5.9167	5.9641	5.9234	6.0111	4.1217	3.9076	3.5955	x
PE 18:1_18:2	6.2679	6.1481	6.1630	6.2476	6.3702	6.3548	6.4741	x
PE 18:1_18:2_1	5.2768	5.3834	5.2304	5.3687	5.5219	5.4982	5.5195	
PE 18:1_20:4_1	4.9914	4.9586	4.8177	4.8283	4.3434	4.3944	4.2323	
PE 18:1_22:6	6.3289	6.2439	6.1602	6.2477	4.9225	4.9002	4.9523	
PE 18:2_18:2	5.6392	5.5082	5.5048	5.7410	5.0316	4.8983	5.0781	x

PE 19:0_18:2	4.9259	4.8437	4.9423	4.9393	4.6111	4.6422	4.7517	
PE 19:0_20:4	4.8651	4.7516	4.7023	4.7053	4.3157	4.1805	4.3482	
PE 20:0_18:1	5.5056	5.4989	5.5419	5.5314	5.7193	5.7291	5.7319	
PE 20:0_20:3	5.4383	5.4184	5.4772	5.4891	5.5894	5.6227	5.6578	
PE 20:0_20:4	5.7795	5.8967	5.8531	5.8722	5.3910	5.3790	5.4698	
PE 20:0_20:4_1	4.4741	4.6438	4.7219	4.7922	4.2847	4.2744	4.3929	
PE 20:1_20:4	5.6061	5.5640	5.5415	5.6914	5.3071	5.2630	5.3213	
PE 20:1_20:4_1	5.5795	5.5529	5.5134	5.4638	5.2642	5.1307	5.2550	
PE 20:1_22:6	5.1538	5.1309	5.0231	5.0084	3.6945	2.7864	3.4853	
PE 40:6	5.2378	5.2572	5.2518	5.3155	4.9209	4.9295	4.9367	x
PE 40:8	5.2261	4.9244	4.8602	4.8060	4.4937	4.4441	4.4303	
PG 16:0_18:2	5.3319	5.2744	5.3140	5.6110	4.9278	4.8076	4.9055	
PG 18:1_20:4	4.7770	4.7618	4.7809	4.8530	4.4969	4.5713	4.6207	
PI 16:0_22:6	5.4887	5.4820	5.4571	5.7362	4.6718	4.3133	4.6460	
PI 17:0_20:4	5.7077	5.6104	5.7337	5.6729	5.0059	4.7569	4.6753	x
PI 18:0_20:4	7.6924	7.6802	7.7162	7.7500	7.6041	7.3058	7.2130	
PI 18:0_22:5	5.4081	5.2631	5.3979	5.1581	4.6757	4.4132	4.4263	
PI 18:0_22:6	5.8774	5.8560	5.8636	6.0357	4.5325	4.1920	4.2008	
PI 18:1_18:2	6.7530	6.6864	6.7562	6.8865	6.5533	6.2461	6.2569	x
PI 18:1_18:3	4.9642	4.8258	4.9183	5.0030	4.6022	4.4588	4.3684	
PI 18:1_20:4	6.4485	6.4957	6.4818	6.3983	6.2605	6.0148	5.9547	
PI 18:1_20:4_1	6.5168	6.3845	6.5715	6.4836	6.3748	6.1000	6.0436	
PI 18:2_20:4	5.1691	4.9248	5.1755	5.0775	4.8072	4.6110	4.6007	
PI 19:0_20:4	5.3395	5.4123	5.3566	5.4469	5.2295	5.1949	5.1279	
PI 19:0_20:4_1	5.4872	5.4389	5.5156	5.4305	4.9720	4.7298	4.6584	
PI 19:1_20:4	4.9494	4.5882	4.9586	4.7033	4.4900	4.3350	4.1607	
PI 20:3_20:4	4.8086	4.7006	4.7833	4.7385	3.7194	3.7194	3.7194	
PI 38:5	5.1975	5.1757	5.3035	4.4768	4.4768	4.4768	4.4768	

**PI 40:4**	**5.4877**	**5.4296**	**5.5157**	**5.6050**	**5.1793**	**4.8360**	**4.7953**	
**PI 40:6**	**5.1639**	**5.1456**	**5.2162**	**5.0517**	**4.5641**	**4.5143**	**4.3158**	
**SM d33:1**	**5.1378**	**5.1412**	**5.0726**	**5.0864**	**5.0103**	**4.9491**	**4.9635**	
**SM d34:1**	**6.1716**	**6.2015**	**6.1506**	**6.2240**	**6.0271**	**6.0213**	**6.0081**	
**SM d34:2**	**5.3331**	**5.3935**	**5.3238**	**5.4374**	**5.2976**	**5.2875**	**5.2756**	
**SM d38:1**	**4.8720**	**4.9493**	**4.8706**	**4.9151**	**4.8053**	**4.7211**	**4.7309**	
**SM d40:1**	**5.8074**	**5.9050**	**5.7990**	**5.9125**	**5.6409**	**5.5901**	**5.5589**	
**SM d40:2**	**5.5908**	**5.6299**	**5.5906**	**5.5964**	**5.4561**	**5.4016**	**5.3637**	
**SM d41:1**	**5.3872**	**5.3919**	**5.3848**	**5.3248**	**5.2235**	**5.1885**	**5.0862**	
**SM d42:1**	**5.6308**	**5.7262**	**5.7115**	**5.7385**	**5.6008**	**5.5408**	**5.5227**	
**SM d42:2**	**6.2167**	**6.2076**	**6.2351**	**6.2082**	**6.1298**	**6.1352**	**6.0784**	
**SM d42:3_1**	**5.5035**	**5.5696**	**5.5710**	**5.5766**	**4.9195**	**4.8175**	**4.9350**	
**TG 15:0_16:0_18:2**	**5.4829**	**5.2461**	**5.4889**	**5.2757**	**4.9997**	**4.9432**	**5.0460**	
**TG 15:0_18:2_18:2**	**5.4594**	**5.2358**	**5.3987**	**5.3775**	**4.6555**	**4.7044**	**4.0722**	
**TG 16:0_16:1_18:2**	**6.7846**	**6.6246**	**6.6975**	**6.7542**	**6.3987**	**6.4289**	**6.4109**	
**TG 16:0_17:0_18:1**	**5.2801**	**5.1380**	**5.1921**	**5.1748**	**4.8901**	**4.8528**	**4.5970**	
**TG 16:0_17:1_18:1**	**5.8129**	**5.6290**	**5.8266**	**5.6438**	**5.3052**	**5.3234**	**5.3774**	
**TG 16:0_18:1_18:2**	**7.3617**	**7.2473**	**7.3317**	**7.4075**	**7.0052**	**7.0112**	**7.0520**	
**TG 16:0_18:1_19:0**	**5.1423**	**4.9864**	**5.0082**	**4.8500**	**4.1397**	**4.6252**	**4.5702**	
**TG 16:0_18:1_20:3**	**5.4018**	**5.4136**	**5.4656**	**5.4895**	**5.2562**	**5.2126**	**5.2747**	
**TG 16:0_18:1_20:4**	**6.4326**	**6.3604**	**6.4969**	**6.3952**	**5.5972**	**5.5759**	**5.6042**	
**TG 16:0_18:1_21:1**	**5.2218**	**5.1488**	**5.2669**	**4.9650**	**4.8100**	**4.7870**	**4.9063**	
**TG 16:0_18:1_22:5**	**5.8312**	**5.7578**	**5.8578**	**5.5975**	**4.2265**	**3.9534**	**4.9373**	
**TG 16:0_18:1_22:6**	**6.2914**	**6.1698**	**6.2565**	**6.3596**	**3.3710**	**4.3866**	**4.1605**	
**TG 16:0_18:2_18:2**	**7.1900**	**7.1200**	**7.1776**	**7.3041**	**6.6226**	**6.6350**	**6.6702**	x
**TG 16:0_18:2_18:3**	**6.4734**	**6.4063**	**6.4318**	**6.6438**	**5.1787**	**5.8360**	**5.8984**	
**TG 16:0_18:2_20:4**	**6.4366**	**6.3972**	**6.4502**	**6.4186**	**5.4154**	**5.4305**	**5.4566**	
**TG 16:0_18:2_22:6**	**6.0213**	**6.0069**	**5.9879**	**6.3113**	**4.4412**	**2.5209**	**3.2190**	

TG 16:1_16:1_18:2	5.9893	5.8825	5.8818	5.9827	5.6853	5.6180	5.6329	x
TG 17:0_18:1_18:2	5.8377	5.6818	5.7461	5.5994	5.2473	5.1105	5.1927	
TG 17:1_18:1_18:2	5.6338	5.5115	5.6399	5.5582	4.8785	4.9032	4.9835	
TG 17:1_18:1_20:4	4.9077	4.6792	5.0003	4.9230	3.2395	3.9622	3.9384	
TG 17:1_18:2_18:2	5.1445	4.9507	5.2137	5.2666	4.4040	4.2527	4.2227	
TG 18:0_18:0_18:0	6.2328	6.1966	6.1560	6.1460	6.1202	6.0726	6.0063	
TG 18:0_18:1_20:4	5.7084	5.6491	5.6787	5.6545	5.1255	5.0734	5.0777	
TG 18:1_18:1_18:2	6.9999	6.8551	7.0287	7.0939	6.5454	6.5773	6.6038	
TG 18:1_18:1_20:1	6.1290	5.9601	6.1279	6.0840	5.7352	5.7844	5.8766	
TG 18:1_18:2_18:2	6.9534	6.8280	7.0500	7.1484	6.1781	6.1831	6.2553	
TG 18:1_18:2_19:0	5.4405	5.0622	5.4347	5.1719	4.7795	4.7887	4.9425	
TG 18:1_18:2_20:1	6.3623	6.1806	6.3280	6.3131	5.8723	5.8611	5.9691	
TG 18:1_18:2_20:2	6.4062	6.2102	6.3853	6.3660	5.7518	5.7940	5.8324	
TG 18:1_18:2_20:4	6.2946	6.2726	6.3158	6.3141	5.2470	5.1188	5.1630	
TG 18:1_18:2_22:0	5.6219	5.3184	5.5788	5.5567	5.2028	5.1867	5.2523	
TG 18:1_18:2_22:1	5.7131	5.4971	5.6198	5.5684	5.1507	5.0035	5.2097	
TG 18:1_18:2_22:5	6.0871	6.0068	6.1128	5.5358	4.0375	4.4914	4.8090	
TG 18:1_20:4_22:5	5.1723	5.2933	5.3472	5.1815	3.1951	3.8941	3.1951	
TG 18:1_20:4_22:6	5.3993	5.4919	5.2693	5.4150	4.5703	4.5703	4.5703	
TG 18:2_18:2_18:2	6.5337	6.4067	6.7001	6.8830	5.4633	5.1089	5.6414	x
TG 18:2_18:2_18:3	5.7860	5.6178	5.9829	6.3656	4.5417	3.7342	4.3319	
TG 18:2_18:2_19:0	5.3440	5.4357	5.4150	5.3142	4.9078	4.6173	4.7874	
TG 18:2_18:2_19:1	4.5233	4.8627	4.5067	4.4151	3.5136	2.8149	3.7914	
TG 18:2_18:2_20:4	5.8609	5.9494	5.9628	6.0499	4.8724	4.9825	4.4480	
TG 18:2_18:2_22:6	5.8196	5.9617	5.8145	5.9618	4.1579	4.1407	4.1722	
TG 18:2_20:5_22:6	4.6069	4.7229	4.6314	4.9904	3.4666	3.4666	3.4666	x
TG 49:2	5.4927	5.2461	5.4888	5.2757	4.9967	4.9109	5.0460	x
TG 51:3	5.8375	5.6529	5.8236	5.7262	5.1787	5.2186	5.3030	

TG 51:3_1	5.8456	5.6654	5.8223	5.7317	5.1787	5.2261	5.3030	
TG 51:5	4.7280	4.9494	5.0578	4.5730	2.8029	2.8029	2.8029	
TG 53:8	4.2775	4.3629	4.3318	3.3089	3.3089	3.3089	3.3089	
TG 54:7	5.7860	5.6183	5.9829	6.3656	4.7567	4.7913	4.3319	
TG 54:7_1	5.6206	5.9733	5.5900	6.1849	5.1734	5.2242	3.8781	
TG 55:7	4.8369	3.7130	4.2560	4.7047	3.0141	3.0141	3.0141	
TG 56:6	6.2522	6.3748	6.4369	6.3718	5.4655	5.4180	5.5322	
TG 56:6_1	5.9613	5.9508	6.0982	6.2153	5.2458	5.1315	4.9210	
TG 56:9	5.6875	5.7406	5.7226	6.0049	4.0795	3.0288	3.7277	
TG 57:2	4.8101	4.7608	4.9668	4.7923	4.6773	4.2617	4.6005	
TG 58:11	5.2175	5.4226	5.3058	5.5120	4.3497	4.3497	4.3497	
TG 58:12	3.9769	4.3356	4.0800	4.9260	3.1530	3.1530	3.1530	
TG 58:7	6.1360	5.9393	5.7931	5.8577	3.4548	4.9509	5.1986	
TG 58:8	6.1210	6.0060	6.1131	5.7478	4.0202	4.4707	4.4471	
TG 60:12	5.3734	5.4903	5.2262	5.4122	4.3741	4.3741	4.3741	
TG 62:12	4.5371	4.4410	4.5943	4.6226	3.7421	3.7421	3.7421	
TG 62:13	4.7791	5.0212	4.6902	4.9388	3.9912	3.9912	3.9912	
TG 64:12	4.5277	4.2340	4.0890	3.7864	3.0875	3.0875	3.0875	

Red denotes upregulation, blue denotes downregulation.

Supplementary Table 10: Significantly-Altered Plasma Lipids in Male *Tmem135*/Leptin Mutant (FUN025/*ob*) Mice Compared to Leptin mutant (*ob*) Mice.

Lipid Species	Normalized Log10 Lipid Concentrations												Contains DHA
	ob 1	*ob* 2	*ob* 3	*ob* 4	*ob* 5	*ob* 6	*ob* 7	ob 8	*FUN025/ob* 1	*FUN025/ob* 2	*FUN025/ob* 3	*FUN025/ob* 4	
ACar 14:1	4.0722	3.7630	4.0815	3.8566	3.5481	4.0298	4.1784	3.7164	4.3060	4.4116	4.2333	4.1024	
ACar 20:1	3.5195	3.5193	2.9542	3.9514	3.7764	3.6991	3.9278	3.3642	2.1164	2.8144	3.1727	2.1164	
CE 16:0	4.9433	4.8848	4.9425	4.9010	4.8546	4.8682	4.9354	5.0091	4.7511	4.8175	4.7175	4.7093	
CE 18:3	5.3270	5.3101	5.2450	5.3513	5.3739	5.1159	5.2527	5.3112	5.4858	5.5838	5.3733	5.5282	
CE 20:4	7.3056	7.3089	7.3344	7.3782	7.0123	7.2883	7.4030	7.4572	6.4954	6.6801	6.4703	6.5329	
CE 20:5	5.9474	5.9254	5.5293	5.9572	5.4902	5.5697	5.6027	5.6232	5.1355	5.2635	5.0754	5.1967	
CE 22:5	5.3807	5.1066	5.7784	5.3233	4.7535	5.6097	5.6692	5.6371	4.3432	4.4993	4.3539	3.9736	
CE 22:6	6.7208	6.7662	6.6151	6.7641	6.4870	6.6690	6.7121	6.8038	5.0257	5.2175	5.0257	5.0918	x
Cer_ADS d26:0_15:0	5.6376	5.6527	5.6177	5.7690	5.5958	5.5557	5.7442	5.6038	5.5855	5.5376	5.4059	5.5219	
Cer_ADS d40:0	5.7449	5.7121	5.7490	5.9309	5.7179	5.6758	5.8239	5.8009	5.6812	5.6384	5.5267	5.5680	
Cer_AS d26:2_16:0	4.7352	4.7395	4.7731	4.8452	4.6222	4.6911	4.8270	4.7557	4.5174	4.5924	4.2279	4.4518	
Cer_NDS d18:0_16:0	5.2363	5.3023	5.3862	5.3531	5.2869	5.3432	5.3676	5.3829	5.2724	5.3085	5.2209	5.1678	
Cer_NP t18:0_20:0	4.9489	4.9553	4.9874	5.0811	4.9567	4.9396	5.1035	5.0720	4.8852	4.8975	4.7107	4.7166	
Cer_NP t18:0_21:0	4.8397	4.7923	4.7831	4.9516	4.9367	4.8399	4.9759	4.7901	4.6088	4.7248	4.4376	4.5750	
Cer_NP t18:0_22:0	5.7449	5.7121	5.7339	5.9309	5.7179	5.6758	5.8239	5.8009	5.6812	5.6384	5.5267	5.5680	
Cer_NP t18:0_23:0	5.6384	5.6527	5.6128	5.7690	5.5961	5.5558	5.7453	5.6038	5.5865	5.5376	5.3842	5.5227	
Cer_NP t41:1	4.7850	4.8428	4.8719	4.9123	4.8719	4.8173	4.9868	4.8684	4.6647	4.8140	4.4998	4.6031	
Cer_NS d18:1_22:1	5.8923	5.9693	5.8473	5.8210	5.8452	5.8671	6.0449	6.3689	5.3970	5.6715	5.3858	5.4639	
Cer_NS d18:2_24:1	5.8179	5.8852	5.7581	5.8558	5.7957	5.7504	5.8820	5.9882	5.6078	5.8253	5.5624	5.6541	
DG 16:0_18:1	4.6195	4.6031	4.6086	4.5107	4.4418	4.5770	4.7228	4.5432	4.8859	4.9332	4.7788	4.7843	
DG 18:1_20:4	4.2312	4.4749	4.1609	3.8339	4.3880	4.3685	4.4897	4.6016	2.9750	3.6890	3.5452	2.2765	
EtherOxPC 18:1e_20:3+1O	6.3095	6.1288	6.2541	6.2696	6.3569	6.3177	6.3300	6.1834	6.0803	6.1085	6.0682	6.1035	
EtherPC 14:0e_8:0	5.1148	5.1704	5.1580	5.1534	5.3630	5.0882	5.1500	5.1396	5.0238	5.0109	4.9444	5.0412	
EtherPC 16:0e_18:2	5.0444	5.0661	4.9531	5.0766	5.7016	5.3122	5.2843	5.1914	5.7676	5.6209	5.6234	5.5089	

EtherPC 16:1e_18:2	5.6674	5.3758	5.7899	5.6226	5.8346	5.6742	5.7541	5.6313	6.0132	5.9560	5.9431	5.9885	
EtherPC 16:1e_20:4	5.6179	5.5047	5.8285	5.6257	5.6405	5.7211	5.7612	5.8395	5.1563	5.0026	4.8571	5.1030	
EtherPC 16:1e_22:6	5.3598	5.4228	5.3667	5.3998	5.4581	5.3776	5.3752	5.4086	5.0645	5.0966	4.9218	5.0210	x
EtherPC 18:0e_20:4	5.7971	5.8573	5.9636	5.8650	5.9148	5.9103	5.9499	5.9931	5.4752	5.4578	5.4195	5.4329	
EtherPC 18:0e_22:4	5.5672	5.6311	5.7293	5.6616	5.5672	5.7084	5.7261	5.7908	5.1895	5.0998	5.0279	5.1319	
EtherPC 18:1e_18:2	4.1791	4.5704	4.6992	4.7032	5.0203	4.5070	4.5162	4.6613	5.0127	4.9159	4.9177	5.0142	
EtherPC 18:1e_20:4	6.1476	6.1158	6.1676	6.1161	6.1756	6.1777	6.1643	6.1894	5.9866	5.9042	5.8698	5.9542	
EtherPC 18:1e_22:5	5.1414	5.1343	4.9120	4.9213	5.2273	5.1441	4.9914	4.7583	3.4155	4.5189	4.4710	4.7908	
EtherPC 24:0e	5.3970	5.3722	5.4236	5.4552	5.6563	5.3968	5.4254	5.4422	5.3392	5.2733	5.2496	5.3679	
EtherPC 32:1e	4.9404	4.4455	4.6112	4.5800	4.5964	4.8577	3.4906	4.7006	5.2120	4.9143	5.3899	5.3933	
EtherPE 16:0e_18:1	4.9768	4.9666	5.1753	4.8877	4.8732	5.0465	5.1191	5.2005	5.3740	5.3219	5.1878	5.3125	
EtherPE 16:1e_18:2	5.7431	5.7253	5.9626	5.6871	5.9325	5.6952	5.8756	5.8331	6.0386	5.9826	5.8800	5.9959	
EtherPE 16:1e_20:4	6.1893	6.1651	6.3806	6.1950	6.1603	6.1996	6.3990	6.4375	5.5925	5.7785	5.6376	5.7715	
EtherPE 16:1e_22:5	5.3733	5.4507	5.8103	5.5432	5.5512	5.5891	5.7470	5.6720	5.0683	5.0850	5.0568	5.1037	
EtherPE 16:1e_22:6	6.3276	6.3665	6.4812	6.3903	6.5140	6.4138	6.4787	6.5411	5.5443	5.5976	5.4428	5.5077	x
EtherPE 18:0e_20:4	6.1966	6.1916	6.3619	6.2363	6.0152	6.2459	6.3969	6.3726	5.7378	5.7787	5.6344	5.7324	
EtherPE 18:0e_20:4_1	4.8851	4.9938	5.0881	4.9851	5.1692	5.0236	4.9845	5.0763	4.5895	4.6574	4.5578	4.7681	
EtherPE 18:0e_20:5	5.1012	4.9196	5.1349	5.3507	5.0815	5.0278	5.2105	5.0103	4.8295	4.7762	4.4682	5.0244	
EtherPE 18:0e_22:4	5.6982	5.7093	6.0291	5.8189	5.8321	5.7955	5.9183	5.9244	5.3379	5.3427	5.2590	5.3664	
EtherPE 18:0e_22:5	5.1566	5.2600	5.5521	5.3225	5.3650	5.3306	5.5077	5.5313	5.2068	4.7955	4.7688	5.0637	
EtherPE 18:1e_20:4	6.1415	6.1516	6.2740	6.2407	6.1985	6.1879	6.3033	6.3105	5.6424	5.7933	5.5548	5.6469	
EtherPE 18:1e_22:5	5.0517	4.9642	5.4250	5.0644	5.0265	5.2130	5.3298	5.3307	4.6732	4.7034	4.5841	4.4635	
EtherPE 18:1e_22:6	6.4042	6.4997	6.5351	6.4885	5.8745	6.4659	6.5797	6.6362	5.1352	5.2972	5.0104	5.0871	x
EtherPE 18:2e_18:2	5.7208	5.7613	5.8926	5.7591	5.9394	5.7119	5.9060	5.7832	5.7590	5.6107	5.5062	5.6305	
EtherPE 18:2e_18:2_1	4.2922	3.1255	3.8245	3.1255	3.1255	3.1255	3.8637	4.2393	5.3619	5.3906	4.5039	5.3233	
EtherPE 18:2e_20:4	5.8099	5.7676	5.9263	5.8544	5.6567	5.7028	5.9908	5.9339	5.4511	5.5629	5.2521	5.4320	
EtherPE 18:2e_22:6	5.8926	5.9080	5.9191	5.9663	5.8288	5.9386	5.9744	5.9667	4.5800	4.8495	4.7336	4.5581	x
EtherPE 38:6e	5.7448	5.7787	5.9181	5.7175	6.0719	5.8307	5.8775	5.9248	5.2687	5.1128	5.0507	5.1106	

EtherPE 40:6e	6.2390	6.3089	6.3498	6.2989	6.4635	6.3222	6.3735	6.3684	5.0073	5.1161	4.5641	4.9531	
FA 16:0_1	5.2543	5.2388	5.3181	5.2559	5.3085	5.2526	5.3453	5.3410	5.2609	5.1206	5.1392	5.2745	
FA 16:1	5.5204	5.4090	5.8504	5.5131	5.5729	5.5507	5.5617	5.5243	5.6331	5.8909	5.8242	5.7682	
FA 20:3_1	4.8268	4.6840	4.9249	4.8116	4.9902	4.7655	4.6994	4.8012	4.6274	4.5357	4.5425	4.6701	
FA 22:4	4.7618	4.6428	5.0072	4.7874	5.0111	4.9165	4.8294	4.8567	4.2354	4.1965	4.1413	4.1912	
HexCer_AP t18:0_16:0	6.0356	6.0775	6.2079	6.1488	6.0046	6.0596	6.2343	6.2734	5.9491	6.0539	5.8215	5.9380	
HexCer_NDS d40:2	5.7642	5.7874	5.7285	5.6510	5.7885	5.7814	5.8342	5.9677	5.6600	5.6826	5.6007	5.6452	
HexCer_NDS d40:2_1	5.8327	5.7859	5.7285	5.6510	5.7885	5.8138	5.8342	5.9686	5.6600	5.6929	5.6007	5.6452	
HexCer_NDS d42:2	6.3806	6.3304	6.4021	6.3398	6.2065	6.3865	6.3896	6.3969	6.4861	6.5757	6.4534	6.4981	
HexCer_NDS d42:2_1	6.3806	6.3292	6.4021	6.3446	6.2010	6.3865	6.3897	6.3969	6.4861	6.5757	6.4545	6.4989	
HexCer_NDS d43:1	4.7630	4.7706	4.8877	4.9462	4.5942	4.8920	4.8704	4.7148	4.6588	4.8219	4.7557	4.9165	
LPA 16:0	4.6015	4.6038	4.6905	4.6336	4.6156	4.5785	4.7135	4.7197	4.6104	4.5261	4.5110	4.5611	
LPA 18:0	4.5079	4.5038	4.5044	4.5845	4.5230	4.4714	4.5632	4.5754	4.4132	4.4833	4.4309	4.4419	
LPC 0:0/18:0	5.3253	5.2343	5.3667	5.4234	5.3403	5.2164	5.3550	5.3584	5.2315	5.1749	5.2111	5.2521	
LPC 0:0/20:4	5.3538	5.6079	5.2446	5.3427	5.1927	5.5921	5.7593	5.7647	4.4981	4.8738	4.4874	4.4544	
LPC 0:0/22:5	4.2616	3.9560	4.3382	4.2151	4.7047	3.8368	3.9511	4.1136	3.4873	3.2752	3.6392	3.7663	
LPC 0:0/22:5_1	4.0339	4.1694	3.7574	3.8223	3.7593	3.9375	4.1291	4.2341	2.3548	3.0535	2.3548	2.3548	
LPC 0:0/22:6	5.0246	5.2713	4.8849	5.0257	5.0516	5.2020	5.2546	5.3492	3.3025	3.8776	3.5191	3.5458	x
LPC 0:0/24:1	4.0552	3.6066	4.0906	4.2265	4.2225	4.0244	3.8483	4.0147	3.8090	3.5652	3.5213	3.5915	
LPC 15:0/0:0	4.6443	4.6272	4.8584	4.6813	4.8918	4.8214	4.7546	4.7971	4.5981	4.5718	4.5511	4.5919	
LPC 16:0/0:0	7.1802	7.1845	7.2642	7.2180	7.2431	7.1879	7.2659	7.3011	7.1796	7.0991	7.1204	7.1862	
LPC 16:1/0:0	5.9028	5.8016	6.0217	5.8669	5.7455	5.9261	5.9015	5.9090	6.0877	5.9769	6.0283	6.0490	
LPC 17:0/0:0	5.2449	5.2790	5.3345	5.3104	5.5707	5.3248	5.3515	5.4050	4.9658	4.8912	4.9072	4.9949	
LPC 17:0/0:0_1	4.8300	4.8849	5.0333	4.9430	5.1314	5.0124	4.9409	4.9802	4.0789	4.4367	4.4275	4.6029	
LPC 18:0/0:0	7.1541	7.1640	7.1714	7.2097	7.1565	7.1140	7.2005	7.2301	7.0526	7.0866	7.0191	7.0732	
LPC 18:0/0:0_1	6.0653	5.9978	6.1090	6.1484	6.1204	5.9782	6.0808	6.1061	5.9743	5.9200	5.9728	6.0198	
LPC 18:1/0:0	7.0496	6.8909	7.1389	7.0010	6.7883	7.0511	7.0313	7.0260	7.1681	7.1476	7.1176	7.1358	
LPC 18:3/0:0	4.2676	4.2429	4.3265	4.3216	4.5479	4.0714	4.2830	4.2714	4.6442	4.5415	4.5730	4.5661	

LPC 19:0/0:0_1	4.7772	4.7201	4.8183	4.7753	5.1571	4.8488	4.7661	4.7881	4.4650	4.3661	4.2384	4.4989	
LPC 19:1/0:0	4.5481	4.2648	4.6873	4.6229	4.7514	4.6030	4.5749	4.3956	4.2975	3.3540	4.1317	4.3188	
LPC 20:2/0:0_1	4.7715	4.5014	4.8121	4.5592	4.5565	4.7145	4.6474	4.6617	5.2311	5.2244	5.2588	5.2289	
LPC 20:2/0:0_2	4.6873	4.8471	4.3775	4.3625	4.5049	4.9354	5.0185	4.8171	4.1724	4.8184	3.8186	3.8501	
LPC 20:3/0:0_1	4.7244	4.4155	5.0619	4.4453	4.1564	4.8259	4.7483	4.6882	5.1121	5.1784	5.0833	4.9596	
LPC 20:3/0:0_2	5.4881	5.0637	5.7345	5.2632	4.8195	5.5051	5.4375	5.3364	5.8025	5.8006	5.7856	5.7126	
LPC 20:4/0:0	6.6027	6.5039	6.7399	6.6976	6.6665	6.5565	6.5733	6.6781	5.9717	5.9015	5.9384	5.9121	
LPC 20:4/0:0_1	5.9990	6.3143	5.8919	5.9855	5.8760	6.3013	6.4278	5.7445	5.2026	5.5558	5.1316	5.1484	
LPC 20:5/0:0	5.3614	5.0925	5.0990	5.3955	5.3358	4.8548	5.0218	5.0533	4.7793	3.6103	4.7536	4.7603	
LPC 22:1/0:0	4.8145	4.6344	4.8531	4.6391	5.0215	4.7593	4.7004	4.8871	4.6179	4.6564	4.5385	4.5979	
LPC 22:5/0:0	4.3086	3.9490	4.8835	4.1924	4.3551	4.4495	4.4486	4.4466	3.2988	3.4907	3.6330	3.4624	
LPC 22:5/0:0_2	4.9156	4.7585	5.4916	4.7976	5.1220	5.1986	5.0984	4.7161	4.2503	3.6492	4.1420	4.2287	
LPC 22:6/0:0	6.3071	6.3253	6.3195	6.3652	6.5274	6.2955	6.2491	6.3941	3.0688	4.7614	4.7794	4.7959	x
LPC 24:1/0:0	4.7499	4.6860	4.8346	4.7693	4.9337	4.8060	4.8203	4.8381	4.5718	4.5479	4.4731	4.5720	
LPE 16:1	4.1819	4.1015	4.4544	4.1343	4.2489	4.3126	4.3844	4.3362	4.6014	4.5392	4.5499	4.6274	
LPE 18:0	5.7395	5.7713	5.8044	5.8822	5.8293	5.6494	5.8689	5.8532	5.6167	5.7163	5.5560	5.6284	
LPE 18:1	5.4483	5.3379	5.6313	5.4303	5.3680	5.4159	5.4817	5.4400	5.7574	5.6484	5.7015	5.7329	
LPE 18:2	5.5420	5.3044	5.6782	5.6616	5.5689	5.2328	5.2563	5.2869	5.9543	5.6439	5.9040	5.9433	
LPE 20:3	4.7846	4.5769	4.9419	4.6619	4.8195	4.7214	4.7722	4.7582	5.0368	5.0862	5.1917	5.2480	
LPE 20:4_1	5.3592	5.5901	5.4244	5.4306	5.3926	5.6746	5.7638	5.8586	5.0171	5.4703	5.0799	4.9643	
LPE 22:6	5.5184	5.7911	6.0577	5.9613	6.1500	5.8080	5.8204	5.9728	4.8032	4.6486	4.6301	4.7477	x
LPG 18:1	4.2910	4.1054	4.4175	4.3012	4.1699	4.1219	4.0779	4.1709	4.5658	4.4120	4.4674	4.5003	
PC 14:0_16:0	5.0630	5.0653	5.1966	5.1024	5.1619	5.1322	5.1491	5.0780	5.2227	5.2335	5.1824	5.2330	
PC 14:0_18:2	5.7832	5.7730	5.8139	5.7782	5.9124	5.8330	5.8529	5.8302	5.6982	5.6916	5.6078	5.6778	
PC 14:0_20:4	5.2651	5.3574	5.3583	5.3291	5.5047	5.3816	5.4402	5.4494	4.5112	4.6992	4.6131	4.6089	
PC 14:0_22:6	5.3223	5.4282	5.1411	5.3940	5.4238	5.2669	5.3192	5.4106	3.7828	3.9771	3.6953	3.6429	x
PC 15:0_18:2	5.0073	5.0402	5.1801	5.1887	5.5140	5.1383	5.1277	5.2605	4.8704	4.7950	4.7449	4.9859	
PC 15:0_20:4	5.9496	6.0033	6.0151	5.9439	5.9946	5.9758	6.0982	6.1369	5.7762	5.9154	5.5102	5.7156	

PC 15:0_22:6	5.3689	5.5690	5.4335	5.5588	5.5961	5.3765	5.4409	5.5917	4.5790	4.5579	4.6334	4.6106	x
PC 16:0_16:0	6.2261	6.2551	6.2910	6.2719	6.3869	6.2127	6.2767	6.3279	5.9848	5.9456	5.8871	5.9789	
PC 16:0_16:1	6.2790	6.2298	6.4092	6.1848	6.0985	6.3334	6.3735	6.3409	6.6247	6.5788	6.5670	6.6567	
PC 16:0_18:0	5.9585	6.0256	5.9793	6.0310	6.0833	5.8772	6.0489	6.1086	5.5134	5.5055	5.4378	5.5157	
PC 16:0_18:1	7.6328	7.5941	7.7511	7.5863	7.3853	7.6795	7.7359	7.7086	7.8534	7.8815	7.8195	7.8653	
PC 16:0_18:2_1	5.4069	5.4040	5.6501	5.6594	5.4295	5.3178	5.3322	5.3635	5.7180	5.6805	5.5391	5.7543	
PC 16:0_18:3	5.9257	6.0016	6.0495	5.9751	5.9474	5.9104	6.0711	6.0560	6.5258	6.4578	6.3946	6.4941	
PC 16:0_20:4	7.5947	7.6667	7.6707	7.6768	7.5890	7.6187	7.7342	7.7838	7.0408	7.1139	6.9999	7.0214	
PC 16:0_20:4_1	4.7884	4.8326	4.7632	4.7024	4.6451	4.8048	4.8715	4.9858	3.9037	3.5433	4.2607	4.3290	
PC 16:0_20:5	6.4545	6.5592	6.2412	6.5240	6.3567	6.2124	6.3074	6.3166	5.8294	5.8274	5.7604	5.8443	
PC 16:0_22:6	7.5143	7.6253	7.5015	7.6030	7.5367	7.5313	7.5791	7.6611	6.0945	6.1664	6.0636	5.8128	x
PC 16:1_18:2	6.6155	6.6394	6.6300	6.6961	6.8309	6.5928	6.6694	6.6922	6.4556	6.4180	6.2918	6.4617	
PC 16:1_20:4	6.3122	6.3728	6.3952	6.4038	6.2996	6.4162	6.4474	6.5066	5.5226	5.6105	5.4348	5.4676	
PC 16:1_22:5	5.5626	5.4972	5.5543	5.5609	5.3539	5.5433	5.6076	5.5237	4.7935	4.8089	4.7326	4.7404	
PC 16:1_22:6	6.2806	6.3477	6.1171	6.3182	6.1836	6.2812	6.2264	6.3670	4.5454	3.6726	2.9801	4.2000	x
PC 16:2_18:2	4.9018	4.9333	4.9286	4.9549	4.9603	4.9038	5.0008	5.0299	4.4086	4.4445	4.3822	4.2029	
PC 17:0_20:4	5.9999	6.0739	6.0811	6.1218	6.2126	6.1035	6.1337	6.1136	5.3007	5.2592	5.1139	5.2652	
PC 17:0_22:6	3.7613	5.4445	5.5676	5.4181	5.6279	4.8000	4.5916	5.3419	3.6633	4.4005	3.6344	3.8043	x
PC 17:1_18:2	4.9882	4.6968	4.9777	4.9332	5.3256	4.9981	4.8021	4.9745	4.0853	4.7183	4.3527	4.5569	
PC 17:1_22:6	4.4848	4.5621	4.4808	4.5970	4.5599	4.5355	4.3901	4.2649	3.5659	3.5659	3.5659	3.5659	x
PC 18:0_18:0	5.4009	5.4399	5.2773	5.3799	5.5345	5.1619	5.3663	5.5093	4.8607	4.8830	4.8560	5.0061	
PC 18:0_18:1	7.3239	7.2596	7.3377	7.2642	6.8728	7.3221	7.3682	7.3282	7.5712	7.7142	7.5341	7.5756	
PC 18:0_18:1_1	5.0428	4.2921	3.9102	4.0483	4.1565	4.9219	4.8663	3.2113	5.3202	5.4743	5.2383	4.8005	
PC 18:0_20:1	5.8545	5.7072	5.8408	5.6979	5.6423	5.6372	5.6994	5.9177	6.0058	6.1476	5.9907	5.9296	
PC 18:0_20:2	6.5584	6.4611	6.4238	6.4662	6.5542	6.5780	6.5364	6.5144	6.6151	6.7338	6.6042	6.6114	
PC 18:0_20:4	7.5186	7.6116	7.5315	7.6788	7.2900	7.5035	7.6725	7.7301	6.6545	6.8756	5.8435	5.8629	
PC 18:0_20:4_1	4.2753	4.4918	4.6401	4.6945	4.3197	3.7651	4.5552	4.5826	3.8336	3.2295	3.9947	3.4398	
PC 18:0_20:5	6.3414	6.3120	6.6056	6.3518	6.0274	6.3630	6.5202	6.5283	5.4630	5.5552	4.3945	5.4298	

PC 18:0_22:5	6.2404	5.9187	6.4775	6.0397	5.4414	6.3880	6.4930	6.4530	5.2045	5.2537	5.1681	5.1418	
PC 18:0_22:5_1	6.1090	6.0204	5.7761	5.9763	5.9790	5.9098	5.2970	6.1538	5.4173	5.5447	5.3812	5.4438	
PC 18:0_22:6	7.3184	7.4078	7.2390	7.3981	7.1802	7.3088	7.3326	7.3705	5.9405	6.0610	5.8697	5.9030	x
PC 18:1_18:2	7.2016	7.0970	7.2010	7.2416	7.0677	7.1475	7.2166	7.1051	7.0703	7.0914	6.9739	7.0241	
PC 18:1_20:3	6.8879	6.8307	6.8626	6.8497	6.5434	6.9394	6.8879	6.8484	6.6575	6.4421	6.6091	6.3992	
PC 18:1_20:4	7.0931	7.0915	7.1164	7.1504	6.9024	7.1925	7.1525	7.1178	6.4795	6.5892	6.3571	6.4641	
PC 18:1_20:4_1	5.0715	5.3450	5.3976	5.2499	5.0463	5.3690	5.3922	5.2698	5.0034	5.1799	4.5000	4.7684	
PC 18:1_22:5	5.8534	5.4693	6.1057	5.8382	5.5817	6.0843	5.7578	5.7929	5.1492	4.8915	3.8966	4.5637	
PC 18:1_22:6	6.8693	6.8657	6.7824	6.8764	6.5770	6.9565	6.8447	6.8012	5.6268	5.7190	5.5829	5.5446	x
PC 18:2_20:4	6.7388	6.7770	6.6788	6.8027	6.7681	6.6419	6.7197	6.7067	6.0140	6.1641	6.0396	6.0267	
PC 18:2_20:5	5.7261	5.8098	5.4152	5.8524	5.9895	5.4297	5.4949	5.4985	4.9920	4.9867	4.8832	5.0476	
PC 18:3_20:4	4.6160	4.8809	4.2900	4.9098	4.9278	4.5657	4.6400	4.5384	2.9895	4.0094	4.0264	4.1080	
PC 18:3_22:6	4.9875	5.1145	4.6283	5.1096	5.2298	4.7474	4.7321	4.8210	3.5159	3.6368	3.5058	3.4603	x
PC 20:0_22:6	5.3109	5.4337	5.1192	5.2171	5.8526	5.0214	5.2496	5.7305	3.9043	3.9338	3.4536	3.4788	x
PC 20:1_20:4	6.1133	6.0783	6.0410	6.1519	5.9740	6.1044	6.1341	6.1850	5.4386	5.5382	5.3884	5.3545	
PC 20:1_22:6	5.8585	5.8011	5.8006	5.8230	5.7710	5.9973	5.8241	5.9144	3.0962	4.2615	4.4688	3.5135	x
PC 20:2_20:4	5.6996	4.9960	4.8101	5.7318	5.3811	5.8022	5.4931	5.0257	3.3941	3.5735	2.6956	3.6072	
PC 20:3_20:4	5.8951	5.8894	5.8035	5.8528	5.6563	5.7717	5.8178	5.9616	5.2559	5.4344	5.2211	5.2300	
PC 20:3_22:6	5.0745	5.0759	5.2259	5.1887	5.2361	5.2893	5.2363	5.2997	4.0509	4.4360	3.5122	4.4089	x
PC 20:4_22:6	5.9167	5.9334	5.7519	5.8554	5.9414	5.8395	5.8095	5.9196	4.0445	4.1420	4.1047	3.8546	x
PC 22:6_22:6	4.8618	4.9061	4.6584	4.8272	4.9015	4.8364	4.7687	4.7823	3.9594	3.9594	3.9594	3.9594	x
PC 30:0	4.9176	4.8918	4.9855	4.9077	4.9143	4.9467	5.0079	5.0041	4.7879	4.8423	4.7261	4.7644	
PC 31:1	3.5942	3.6497	3.8973	3.9419	4.0130	3.2555	3.8091	4.2533	3.5180	3.1162	3.0486	3.2399	
PC 33:1	5.3376	5.2177	5.5670	5.2982	5.3547	5.4528	5.4959	5.5192	5.1822	5.3188	4.9814	5.2023	
PC 35:1	5.5242	5.5018	5.6401	5.4939	5.4870	5.5918	5.6458	5.6052	5.4053	5.4713	5.3065	5.3610	
PC 35:2	5.7421	5.7480	5.7654	5.7962	6.0928	5.6474	5.8029	5.7787	5.5554	5.5339	5.4471	5.5288	
PC 35:2_1	5.3649	5.3122	5.4812	5.2806	5.4962	5.4614	5.4986	5.4611	5.0661	4.9926	4.8128	5.0362	
PC 35:4	5.1432	5.2469	5.3054	5.3144	5.3886	5.1808	5.3519	5.3508	5.0139	4.9785	4.9228	4.9477	

**PC 36:6**	**4.7669**	**4.8225**	**4.7246**	**4.7948**	**4.8109**	**4.7357**	**4.7459**	**4.8263**	**2.9703**	**3.8038**	**3.1988**	**3.6644**	
**PC 37:5**	**5.1140**	**5.2090**	**5.1838**	**5.2194**	**4.6882**	**5.1978**	**5.2343**	**5.2563**	**3.9893**	**3.9893**	**3.9893**	**3.9893**	
**PC 38:5**	**6.4762**	**6.4601**	**6.7276**	**6.5036**	**6.2806**	**6.6154**	**6.7042**	**6.6674**	**5.8024**	**5.8450**	**5.2700**	**5.7362**	
**PC 38:7**	**5.5852**	**5.6669**	**5.4392**	**5.6185**	**5.4766**	**5.5887**	**5.5817**	**5.7249**	**3.7456**	**3.9920**	**3.3110**	**3.5071**	
**PC 38:7_1**	**4.9949**	**5.2056**	**5.0013**	**5.0704**	**4.7250**	**4.9927**	**5.1529**	**5.3851**	**2.4478**	**3.5776**	**3.4800**	**3.1465**	
**PC 39:4**	**5.0119**	**5.1474**	**4.8270**	**5.1005**	**5.4115**	**5.1501**	**5.2373**	**5.3587**	**3.9299**	**4.2701**	**3.8778**	**3.7832**	
**PC 39:5**	**4.8886**	**4.7591**	**4.8114**	**4.9384**	**4.7878**	**4.9236**	**4.8649**	**4.8282**	**4.3370**	**4.4418**	**4.2848**	**4.3496**	
**PC 40:4_1**	**4.8231**	**4.8306**	**4.6253**	**4.7385**	**5.4893**	**5.0046**	**4.6535**	**5.3471**	**4.2485**	**4.7719**	**3.8334**	**4.3047**	
**PC 40:5**	**6.1079**	**6.0783**	**6.0410**	**6.1500**	**5.9826**	**6.0707**	**6.1341**	**6.1869**	**5.4370**	**5.5052**	**5.3966**	**5.3524**	
**PC 40:6**	**5.6474**	**5.0938**	**6.0150**	**5.1292**	**5.1674**	**5.8940**	**5.8773**	**5.8354**	**4.5470**	**4.6986**	**4.8034**	**4.2404**	
**PC 40:6_1**	**5.6330**	**5.6694**	**5.3987**	**5.4610**	**5.5679**	**5.3877**	**5.5429**	**5.5400**	**3.9791**	**4.2505**	**3.2802**	**4.0197**	
**PC 40:7**	**5.5699**	**5.5239**	**5.4337**	**5.5192**	**5.3172**	**5.4458**	**5.5277**	**5.6687**	**4.8197**	**5.0750**	**4.7346**	**4.8689**	
**PC 40:8**	**6.3266**	**6.3687**	**6.2446**	**6.3460**	**6.4059**	**6.2898**	**6.2787**	**6.3757**	**4.9441**	**5.1769**	**4.9545**	**4.9114**	
**PC 40:9**	**4.3582**	**4.4495**	**4.0762**	**3.9817**	**4.6134**	**4.1337**	**4.0896**	**3.9887**	**2.9286**	**2.9095**	**3.2826**	**3.5231**	
**PC 41:6**	**4.9881**	**5.0386**	**5.0265**	**5.0311**	**5.2047**	**5.0752**	**5.0568**	**5.0991**	**3.8498**	**3.4268**	**3.3844**	**3.4888**	
**PC 41:7**	**4.5174**	**4.1364**	**4.5487**	**4.0923**	**2.7886**	**4.6376**	**4.4493**	**4.5673**	**3.3877**	**3.3692**	**3.0339**	**2.8687**	
**PC 42:10**	**5.2518**	**5.2701**	**5.1006**	**5.2167**	**5.2572**	**5.1496**	**5.1570**	**5.2643**	**3.5516**	**2.9991**	**2.3006**	**3.0599**	
**PC 42:4**	**4.0474**	**3.8141**	**3.0058**	**4.1925**	**4.4146**	**4.0958**	**3.7780**	**4.3354**	**3.3556**	**3.1949**	**3.4365**	**3.0972**	
**PC 42:6**	**4.5590**	**4.6178**	**4.3367**	**4.4803**	**5.1249**	**4.4156**	**4.5515**	**4.7646**	**2.3225**	**2.3225**	**3.0211**	**2.3225**	
**PC 42:6_1**	**4.3272**	**4.0637**	**3.6431**	**4.3495**	**4.0214**	**4.4671**	**4.2635**	**4.2923**	**3.4647**	**3.1618**	**3.2853**	**3.7677**	
**PC 42:7**	**5.3880**	**5.3213**	**5.3028**	**5.3824**	**5.1054**	**5.4345**	**5.3998**	**5.4292**	**4.2321**	**3.9971**	**3.7723**	**3.7156**	
**PC 42:9**	**4.6628**	**4.8076**	**4.4104**	**4.6361**	**4.7822**	**4.4374**	**4.6907**	**3.6542**	**3.4786**	**3.7007**	**3.4403**	**2.7414**	
**PC 44:4**	**4.0949**	**3.5523**	**3.8841**	**3.6045**	**3.9147**	**3.9825**	**3.8671**	**4.2031**	**3.2243**	**3.3384**	**2.9295**	**2.9903**	
**PE 16:0_16:1**	**4.3654**	**4.1962**	**4.5604**	**4.1896**	**4.2479**	**4.3142**	**4.4111**	**4.2100**	**5.1139**	**5.0865**	**4.9437**	**5.1859**	
**PE 16:0_18:1**	**5.7126**	**5.6481**	**5.8605**	**5.6537**	**5.5601**	**5.7585**	**5.8538**	**5.6560**	**6.3081**	**6.3958**	**6.1731**	**6.2754**	
**PE 16:0_18:2**	**6.0999**	**6.1099**	**6.0864**	**6.1734**	**6.3067**	**5.9862**	**6.1257**	**6.0097**	**6.5370**	**6.4963**	**6.4005**	**6.5285**	
**PE 16:0_18:3**	**4.8024**	**4.6706**	**4.8041**	**4.8595**	**4.9679**	**4.5581**	**4.9396**	**4.8486**	**4.4595**	**3.5533**	**3.5533**	**4.2523**	
**PE 16:0_20:3**	**6.2399**	**6.2202**	**6.4052**	**6.2455**	**6.2391**	**6.2375**	**6.4361**	**6.4575**	**6.0568**	**6.0966**	**5.8590**	**5.9792**	

PE 16:0_20:4_1	4.8999	4.8595	4.9232	4.9167	4.8217	4.8981	4.9945	5.0411	4.7425	4.8391	4.6918	4.6659	
PE 16:0_22:5	5.5198	5.4506	5.7901	5.4494	4.9125	5.7062	5.8042	5.6366	5.0569	5.1068	5.0868	5.1115	
PE 16:0_22:6	6.6697	6.8194	6.6518	6.7595	6.7899	6.7311	6.8044	6.7699	5.0445	5.4404	4.9655	5.0496	x
PE 16:1_20:4	4.9201	4.9834	4.8771	4.9737	5.1704	4.8583	5.0272	4.9932	4.5207	4.6228	4.3890	4.4692	
PE 17:0_20:4	4.7465	4.7933	4.7954	4.8114	4.8707	4.7481	4.9002	4.8504	4.2714	4.4129	4.1652	4.1312	
PE 17:1_22:6	4.7752	4.9316	4.8060	4.9311	4.9207	4.9751	4.9629	4.8938	3.7419	3.9581	3.3957	3.5585	x
PE 18:0_16:1	5.0566	4.9542	4.7964	5.0147	4.2686	4.4566	4.8488	5.0500	5.2845	5.2924	5.2272	5.3082	
PE 18:0_18:1	6.2257	6.1778	6.3313	6.1594	6.0123	6.2652	6.3194	6.2818	6.4390	6.4475	6.3888	6.4368	
PE 18:0_18:1_1	5.2900	5.2751	5.3306	5.3931	5.3285	5.2592	5.5398	5.3004	5.6836	5.7418	5.5238	5.5903	
PE 18:0_20:3_1	6.2227	6.2352	6.3407	6.2854	6.2524	6.2488	6.3748	6.3792	5.8846	6.0694	5.7755	5.8303	
PE 18:0_20:3_2	5.1589	4.9908	5.2963	5.0736	4.9877	5.2356	5.4034	5.2318	5.5249	5.7045	5.4499	5.4495	
PE 18:0_20:4	6.8004	6.9010	6.8311	6.8622	6.7842	6.8126	6.9447	6.9373	6.4804	6.6729	6.3926	6.4036	
PE 18:0_20:4_1	6.1745	6.2495	6.2623	6.2690	6.1895	6.2088	6.3254	6.3483	5.6707	5.7459	5.6160	5.6256	
PE 18:0_22:6	6.2743	6.4432	6.2056	6.3343	6.3565	6.3033	6.4035	6.3958	4.7327	4.9008	4.5103	4.6990	x
PE 18:0_22:6_1	6.0718	6.2175	6.0802	6.1703	6.0996	6.0899	6.1598	6.2454	4.4072	4.2395	3.8546	4.4202	x
PE 18:1_18:2	6.1317	6.0992	6.0290	6.1747	6.1776	6.0403	6.0879	5.9522	6.4082	6.4705	6.2685	6.3509	
PE 18:1_20:3	5.7452	5.7784	5.9691	5.9088	5.7302	5.8394	6.0533	6.0078	5.6525	5.8156	5.4558	5.6143	
PE 18:1_20:4_1	4.8086	5.0930	4.8596	4.9203	4.9764	4.9662	4.7976	4.9605	3.8276	3.9027	3.6919	2.9930	
PE 18:1_22:6	6.3505	6.4235	6.1687	6.3485	6.1600	6.3676	6.3290	6.2775	4.8318	5.1652	4.7781	4.7200	x
PE 18:2_20:2	5.9493	6.0033	6.0084	5.9439	5.9946	5.9709	6.0977	6.1369	5.7754	5.9154	5.5102	5.7156	
PE 18:2_20:4	6.1365	6.0798	5.9417	6.1500	6.0224	5.9329	6.0618	6.0717	5.8705	6.0300	5.5514	5.6795	
PE 19:0_18:2	4.6836	4.5948	4.5865	4.7497	4.8532	4.6043	4.6987	4.8510	5.1296	4.8536	4.9754	5.1254	
PE 20:0_18:1	5.9324	5.8773	5.9540	5.8841	5.5253	5.9382	5.9683	5.9718	6.1587	6.2726	6.1141	6.1449	
PE 20:0_20:3_1	4.4482	4.3906	4.6279	4.7390	5.2845	5.1752	4.6185	4.5287	5.2051	5.4714	5.3403	5.0995	
PE 20:0_20:4	6.0446	6.1434	5.9204	6.1920	6.0158	5.9844	6.1211	6.1743	5.3588	5.4468	5.0869	5.3938	
PE 20:0_22:4	5.5151	5.3910	5.4765	5.5502	5.9871	4.7981	5.7948	5.7994	4.5550	4.2417	4.2614	4.6571	
PE 20:1_18:2	5.7448	5.6987	5.7326	5.6890	5.6848	5.7291	5.7513	5.6364	5.5864	5.6634	5.5505	5.6020	
PE 20:1_20:3	5.9999	6.0789	6.0696	6.1123	6.2144	6.1035	6.1306	6.1777	5.3494	5.3044	5.1139	5.2722	

PE 20:1_20:4	5.6355	5.6644	5.6993	5.6998	5.4599	5.7187	5.7295	5.7079	5.0446	5.4888	5.4043	5.3812	
PE 20:1_22:6	5.4857	5.5003	5.2412	5.3720	4.8745	5.5589	5.0221	5.4277	4.6135	4.5354	4.3981	4.6161	x
PE 20:2_20:4	5.3794	5.5690	5.4311	5.5587	5.5961	5.3704	5.4409	5.5917	4.5790	4.5579	4.6319	4.6106	
PE 20:4_20:4	5.1847	5.0273	4.8707	4.5418	4.6306	3.9518	4.0295	4.8643	3.2528	4.3443	3.2528	3.2528	
PE 38:6	5.2258	5.3630	5.2797	5.2424	5.2132	5.3169	5.2976	5.2915	2.7215	2.7215	3.4204	2.7215	
PE 40:5	6.4727	6.5503	6.5705	6.5330	6.5154	6.5271	6.6326	6.6755	5.5305	5.6884	5.4415	5.4658	
PE 40:7	5.0305	4.9730	5.0370	5.2596	4.8585	4.9450	5.0936	5.2850	3.4004	3.9798	4.6331	3.3415	
PG 16:0_18:2	4.8251	5.1152	4.8976	5.1627	5.5182	4.9044	4.9539	5.1008	4.7100	4.5856	4.6143	4.7474	
PI 16:0_16:1	4.7754	4.8410	4.9942	4.4316	4.8352	4.7983	4.9499	5.1701	5.1498	5.3484	5.1471	5.3736	
PI 16:0_18:1	5.4343	5.5453	5.7817	5.4712	5.6700	5.5553	5.6857	5.6195	6.1669	6.1492	6.0733	6.1912	
PI 16:0_20:3	5.7247	5.4530	5.9522	5.3902	4.8619	5.7806	5.8007	5.6247	6.4847	6.5307	6.4036	6.4532	
PI 16:0_20:3_1	5.8105	5.8381	5.7174	5.6724	5.8881	5.6502	5.7593	5.7265	6.1507	6.1241	6.0410	6.1865	
PI 16:0_22:6	5.1428	5.4327	5.2882	5.1535	5.5230	5.1772	5.3254	5.3748	3.7145	3.8253	3.8271	3.0157	x
PI 17:0_20:4	5.8206	5.8670	5.8501	5.8403	5.9342	5.8266	5.8457	5.8862	5.0873	5.0682	5.0036	5.0707	
PI 18:0_18:1	5.3702	5.4299	5.5684	5.2617	5.3894	5.4258	5.4737	5.3397	5.9940	6.0576	5.9244	5.9793	
PI 18:0_18:2_1	5.7521	5.8987	5.9360	5.6987	6.2999	5.7477	5.8524	5.8139	6.3002	6.2040	6.1860	6.1831	
PI 18:0_20:2	5.3688	5.1214	5.5239	5.1495	4.9618	5.5903	5.4242	5.3072	6.6779	6.6510	6.6359	6.5645	
PI 18:0_20:3	6.6777	6.5330	6.9092	6.4207	5.7386	6.8201	6.8037	6.6034	7.1945	7.2794	7.1726	7.0981	
PI 18:0_20:3_1	6.5401	6.6718	6.4407	6.4367	6.3278	6.4760	6.5416	6.6131	6.6590	6.8989	6.6937	6.7734	
PI 18:0_20:4	7.7683	7.8459	7.4385	7.7577	7.7244	7.7409	7.7892	7.5190	7.4758	7.5654	7.4314	7.5111	
PI 18:0_22:3	4.8939	4.7549	4.9817	4.7872	4.2626	4.9263	4.8479	4.9539	5.3780	5.4797	5.3308	5.2557	
PI 18:0_22:5	5.4128	5.3758	5.6837	5.2093	4.8116	5.6296	5.5551	5.5777	4.8884	4.9592	4.8166	4.5558	
PI 18:0_22:6	5.8292	6.0629	5.7062	5.8952	6.0387	5.8022	5.7488	5.9305	4.4925	4.5877	4.3804	4.5301	x
PI 18:1_20:3	5.4744	5.5694	5.4507	5.2701	5.1143	5.3321	5.2735	5.4355	5.7450	5.7862	5.6702	5.7870	
PI 18:1_20:4	6.9683	6.9940	6.9701	6.9480	6.8121	6.9673	6.9802	7.0146	6.7303	6.8500	6.6280	6.8035	
PI 18:1_22:5	5.1854	5.2823	5.1002	5.2996	5.2032	5.1218	5.1093	5.2236	4.3142	4.5801	4.2205	4.5821	
PI 18:1_22:6	4.8214	4.8869	4.6006	4.8153	4.7556	4.6813	4.6853	4.7532	4.2581	4.4231	4.0483	4.1350	x
PI 18:2_20:4	4.6158	4.9426	5.0279	4.8696	4.0846	4.9975	4.9229	4.7406	2.4821	3.8810	2.4821	3.1801	

PI 38:4	5.6672	5.4201	5.8294	5.1462	4.9968	5.7733	5.6052	5.4852	6.2329	6.3235	6.1801	6.1479	
PI 40:5	5.6394	5.8146	5.4485	5.6540	5.6861	5.5936	5.5213	5.6284	5.2034	5.2912	5.1699	5.1903	
SM d14:3_28:2	5.1742	5.2491	5.4144	5.4791	4.8922	5.3458	5.4336	4.8920	4.5623	4.2855	4.3592	4.4961	
SM d15:1_26:0	6.4031	6.4562	6.4216	6.5431	6.5101	6.4110	6.5007	6.3705	6.1072	6.1452	6.0042	6.1562	
SM d18:1_22:0	6.2745	6.3992	6.2177	6.3231	6.6252	6.2185	6.3480	6.5238	5.9909	6.0919	5.8991	5.9932	
SM d18:2_23:0	6.1160	6.1289	6.0586	6.2359	6.3608	6.1482	6.1886	6.1075	5.9336	5.9099	5.8633	6.0339	
SM d32:1	5.3783	5.3343	5.4618	5.4597	5.2865	5.3896	5.4595	5.3660	5.2482	5.2159	5.1548	5.2255	
SM d33:1	5.3532	5.2724	5.4358	5.5934	5.5530	5.4634	5.5009	5.3974	5.2574	5.1254	5.0974	5.2360	
SM d34:0	5.0618	5.0073	5.1963	5.1697	5.1560	5.1086	5.1722	5.2261	5.0695	5.0771	4.9354	5.0109	
SM d34:1	6.2398	6.2389	6.3085	6.3369	6.1478	6.2040	6.3706	6.3516	5.9211	6.0065	5.8306	5.8947	
SM d34:2	6.1774	6.1892	6.1918	6.2340	6.2588	6.1701	6.2369	6.2631	6.0526	6.0910	5.9388	6.0394	
SM d35:1	3.5993	2.9827	4.5629	4.5320	4.5209	4.4436	4.0583	4.5186	4.3184	4.2809	3.7549	3.8273	
SM d36:1	5.9156	5.9368	6.0309	6.0023	5.8685	5.9121	6.0725	6.0517	5.6555	5.7163	5.5659	5.6153	
SM d38:1	5.2434	5.6062	5.4612	5.4921	5.5190	5.4676	5.6233	5.6984	5.0286	5.2009	5.0324	5.0513	
SM d39:1	5.4654	5.5301	5.4038	5.5896	6.0506	5.3776	5.5329	5.5776	5.2830	5.1782	5.1345	5.3028	
SM d39:2	5.0755	4.9929	5.1628	5.0429	4.3400	5.1323	4.8957	5.0573	4.7062	4.3340	4.2533	3.4862	
SM d40:1_1	5.4693	5.5962	5.4450	5.5314	5.8036	5.3994	5.5808	5.7316	5.1641	5.2905	5.1135	5.1461	
SM d40:2	6.0018	6.0648	5.9548	5.9905	6.2891	6.0100	5.8537	6.2623	5.6026	5.5978	5.5330	5.6096	
SM d40:3	4.9650	5.0279	4.8539	4.9483	5.1863	4.9781	5.0561	5.1221	4.7215	4.7444	4.4449	4.7468	
SM d41:1	5.2917	5.3220	5.3062	5.3814	5.3827	5.2469	5.3672	5.2233	5.0549	5.0518	4.9140	5.0135	
SM d41:2	5.2541	5.1537	5.1604	5.2747	5.4038	5.1417	5.2805	4.7572	4.9940	5.0672	4.9325	4.4198	
SM d42:1	6.2922	6.3346	6.2889	6.3718	6.5158	6.2371	6.3290	6.3182	6.0637	6.1120	5.9245	6.0783	
SM d42:2	6.9287	6.9275	6.9302	6.9008	6.8500	6.9304	6.9815	6.9617	6.7853	6.8615	6.7550	6.7919	
SM d42:3	5.8544	5.9513	5.8746	6.0279	5.6223	5.8486	6.0220	6.0846	4.8534	5.1077	4.8059	4.7303	
SM d42:3	6.2247	6.2325	6.1740	6.2402	6.3575	6.2006	6.2272	6.2402	5.9811	5.9876	5.9400	6.0099	
SM d43:2	5.2886	5.2640	5.2983	5.3681	5.3053	5.2906	5.4048	5.2456	5.1652	5.1167	4.8685	5.1742	
TG 15:0_16:0_16:1	5.8645	5.8620	5.8719	5.8937	5.6638	5.8623	5.9406	5.9223	5.3939	5.5317	5.3717	5.4295	
TG 16:0_16:0_16:0	5.3225	5.2522	5.4607	5.3069	5.3618	5.2461	5.2977	5.3123	5.7219	5.5681	5.5669	5.6586	

TG 16:0_16:0_18:0	4.9119	5.2151	5.2531	5.1505	5.2344	5.1124	4.9314	4.9264	5.4185	5.3783	5.1629	5.3328	
TG 16:0_16:0_18:1	6.2353	6.2738	6.4767	6.2162	6.3717	6.2266	6.4692	6.4241	6.9014	6.7917	6.8031	6.8584	
TG 16:0_16:1_18:1	6.3944	6.4807	6.6242	6.4030	6.7237	6.3514	6.6410	6.5661	6.8035	6.6500	6.6747	6.7897	
TG 16:0_18:1_18:1	7.0946	7.1419	7.3096	7.0541	7.1275	7.1012	7.3759	7.2663	7.5223	7.5098	7.4561	7.4819	
TG 16:0_18:1_20:1	6.0272	6.0297	6.1165	5.9403	6.1242	6.0215	6.2129	6.2191	6.4786	6.5378	6.4438	6.3821	
TG 16:0_18:1_20:4	5.7762	5.9401	6.0025	5.8975	6.2222	5.4617	6.0986	6.1729	5.4876	5.4620	5.2844	5.3968	
TG 16:0_18:1_22:6	5.8533	6.1057	5.8683	5.9774	5.2567	5.4958	5.9706	6.1278	3.8169	3.6700	4.2746	3.3409	x
TG 16:0_18:2_20:4	5.6575	5.9127	5.7854	5.8460	6.4201	5.2973	5.8692	5.9937	5.1457	5.0303	4.2578	4.6024	
TG 16:0_18:2_22:6	5.4542	5.5920	5.1900	5.7216	6.3797	5.4511	5.3044	5.8063	4.1623	3.4622	2.9035	3.5215	x
TG 18:1_18:1_20:1	5.8423	5.7403	5.8689	5.6748	5.9404	5.8247	5.9512	6.0090	6.0975	6.1639	6.0562	5.9604	
TG 18:1_18:1_20:4	6.0300	6.1043	6.0110	6.0496	6.4393	5.9759	6.2337	6.3024	5.5359	5.4640	5.4097	5.4632	
TG 18:1_18:2_20:4	5.0113	5.7824	5.5508	5.7724	6.3001	4.8227	5.4739	5.7305	5.0653	4.7899	4.8230	4.3935	
TG 18:1_18:2_22:6	5.5084	5.6599	5.4835	5.6283	5.9919	5.4188	5.3516	5.6546	4.1149	3.9903	3.6871	4.1044	x
TG 18:2_20:1_22:6	4.8897	3.8527	4.4039	3.9701	5.2283	4.6154	4.6305	5.0640	2.9072	3.5959	3.4189	3.8387	x
TG 18:2_22:6_22:6	4.1609	4.5789	3.4378	4.6151	5.0823	3.8187	4.0341	4.4537	2.2221	2.9204	2.2221	2.2221	x
TG 46:2	4.6729	4.6069	4.6690	4.6493	4.7953	4.6267	5.1996	4.6414	5.0877	4.9260	4.9905	5.0655	
TG 48:0	5.4403	5.4291	5.5784	5.4664	5.5050	5.3596	5.4598	5.4701	5.7902	5.6626	5.6616	5.7368	
TG 48:1	5.3303	5.3159	5.5775	5.2841	5.5873	4.8200	5.6616	5.4764	5.8753	5.7321	5.7802	5.8904	
TG 52:1	5.4946	5.6318	5.7702	5.5346	5.7588	5.5336	5.7545	5.7652	6.2382	6.2346	6.1592	6.1441	
TG 54:1	4.9746	5.0959	4.8095	5.0527	5.3817	4.9305	5.0677	5.2407	5.4743	5.3652	5.2820	5.2517	
TG 56:8	4.7906	5.4302	5.3243	5.4176	6.1344	4.9869	5.0231	5.3662	4.6982	4.6644	4.3484	4.4124	
TG 58:10	4.9983	5.4565	5.2696	5.4490	5.9938	5.0777	4.9317	5.4717	3.4240	2.9287	3.4354	3.3926	
TG 58:10_1	4.9983	5.4565	5.2696	5.4490	5.9941	5.0759	4.6716	5.4717	3.3561	2.9187	3.4284	3.4010	
TG 58:6	5.2535	4.9771	5.3985	4.7996	5.8158	5.3258	5.5350	5.6156	4.7396	5.1223	4.7539	4.6364	
TG 58:7	5.5475	5.7858	5.6057	5.3933	5.6342	4.7918	5.8098	5.4337	5.1733	4.8431	4.8137	4.7093	
TG 58:8	5.7440	5.8927	5.7088	5.8318	5.8370	5.5938	5.7356	6.0030	4.3982	4.4250	3.9102	3.4702	
TG 60:10	4.2138	4.3622	4.7348	4.8323	4.9597	2.8967	4.8873	5.1110	3.7150	3.1435	3.0797	2.1984	
TG 60:12	4.3816	5.0802	4.4972	5.0790	5.5434	4.4081	3.8638	5.0259	2.2377	2.2377	2.2377	2.9361	

**TG 62:13**	**4.4709**	**4.8513**	**4.1658**	**4.8638**	**4.9869**	**3.8836**	**4.0590**	**4.3046**	**2.9676**	**2.9574**	**2.2590**	**3.0249**	
**TG 62:14**	**4.1609**	**4.5789**	**3.4204**	**4.6151**	**5.0827**	**3.8185**	**4.0144**	**4.4526**	**2.2317**	**2.9300**	**2.2317**	**2.2317**	

Red denotes upregulation, blue denotes downregulation.

Supplementary Table 11: Primers Used in this Study.

Gene	Forward Primer	Reverse Primer
Fads1	GAAGAAGCACATGCCATACAACC	TCCGCTGAACCACAAAATAGAAA
Fads2	GCCTGGTTCATCCTCTCGTACTT	GAAAGGTGGCCATAGTCATGTTG
Elovl2	AGCTGCCATGCCCTTTCTGA	CCCTGGGGCTCTGTTGATTATG
Elovl5	ATGGAACATTTCGATGCGTCA	GTCCCAGCCATACAATGAGTAAG
Abcd2	GTTCAAAGAGAAGGAGGATGGGATG	TGCTCACGGCACTGGTACATTC
Rplp0	GGACCCGAGAAGACCTCCTT	GCACATCACTCAGAATTTCAATGG

Supplementary Table 12: Antibodies used for Western blot analysis in this study.

Antibody	Catalogue Number	Company	Antibody Dilution
anti-ACOX1	#10957-1-AP	Proteintech	1:1000
anti-HSD17B4/DBP	#15116-1-AP	Proteintech	1:1000
anti-ACAA1	#12319-1-AP	Proteintech	1:1000
anti-SCP2/SCPx	#23006-1-AP	Proteintech	1:1000
anti-PEX14	#10594-1-AP	Proteintech	1:500
anti-PMP70	#ab3421	Abcam	1:1000
anti-CAT	#C0979	Sigma-Aldrich	1:2000
anti-CYP4A10	#PA3-033	Thermo Scientific	1:1000
anti-CPT1A	#ab128568	Abcam	1:1000
anti-CACT/SLC25A20	#19363-1-AP	Proteintech	1:1000
anti-CPT2	#26555-1-AP	Proteintech	1:1000
anti-ECHS1	#ab153732	Abcam	1:1000
anti-ACSF3	#PA525803	Thermo Fisher Scientific	1:500
anti-MLYCD	#ab95945	Abcam	1:500
anti-SLC25A1	#15235-1-AP	Proteintech	1:1000
anti-APOB	#AB742	Millipore Sigma	1:1000
anti-APOA1	#11A-G2b	Academy Bio-Medical	1:200
anti-ACTB	#4970S	Cell Signaling Technologies	1:500
anti-TUB	#3873S	Cell Signaling Technologies	1:1000
anti-GAPDH	#MCA-1D4	EnCor Biotechnology	1:2000
anti-VIN	#sc-25336	Santa Cruz Biotechnologies	1:500
anti-transferrin	#A80-128A	Bethyl Laboratories	1:1000
anti-HSC70	#sc-7298	Santa Cruz Biotechnology	1:1000
anti-PLIN2	#GP40S	Progen	1:200
