# Supplementary material for: Transmembrane protein 135 regulates lipid homeostasis through its role in peroxisomal DHA metabolism
Source: Commun Biol. 2023 Jan 4;6:8. doi: 10.1038/s42003-022-04404-7 (PMC9813353; doi:10.1038/s42003-022-04404-7)
Supplement: Supplementary file 3 — Description of Additional Supplementary Files [file 42003_2022_4404_MOESM3_ESM.pdf]

Description of Additional Supplementary Files

File name: Supplementary Data 1

Description: The source data behind the graphs and tables in the paper.
