# Supplementary material for: Transmembrane protein 135 regulates lipid homeostasis through its role in peroxisomal DHA metabolism
Source: Commun Biol. 2023 Jan 4;6:8. doi: 10.1038/s42003-022-04404-7 (PMC9813353; doi:10.1038/s42003-022-04404-7)
Supplement: Supplementary file 5 — Reporting Summary [file 42003_2022_4404_MOESM5_ESM.pdf]

nature portfolio

Corresponding author(s): Akihiro Ikeda

Last updated by author(s): 12/08/2022

Reporting Summary

Nature Portfolio wishes to improve the reproducibility of the work that we publish. This form provides structure for consistency and transparency in reporting. For further information on Nature Portfolio policies, see our Editorial Policies and the Editorial Policy Checklist.

Statistics

For all statistical analyses, confirm that the following items are present in the figure legend, table legend, main text, or Methods section.

n/a	Confirmed	
☐	☒	The exact sample size (*n*) for each experimental group/condition, given as a discrete number and unit of measurement
☐	☒	A statement on whether measurements were taken from distinct samples or whether the same sample was measured repeatedly
☐	☒	The statistical test(s) used AND whether they are one- or two-sided<br>*Only common tests should be described solely by name; describe more complex techniques in the Methods section.*
☒	☐	A description of all covariates tested
☐	☒	A description of any assumptions or corrections, such as tests of normality and adjustment for multiple comparisons
☐	☒	A full description of the statistical parameters including central tendency (e.g. means) or other basic estimates (e.g. regression coefficient) AND variation (e.g. standard deviation) or associated estimates of uncertainty (e.g. confidence intervals)
☐	☒	For null hypothesis testing, the test statistic (e.g. *F*, *t*, *r*) with confidence intervals, effect sizes, degrees of freedom and *P* value noted<br>*Give P values as exact values whenever suitable.*
☒	☐	For Bayesian analysis, information on the choice of priors and Markov chain Monte Carlo settings
☒	☐	For hierarchical and complex designs, identification of the appropriate level for tests and full reporting of outcomes
☒	☐	Estimates of effect sizes (e.g. Cohen's *d*, Pearson's *r*), indicating how they were calculated

Our web collection on statistics for biologists contains articles on many of the points above.

Software and code

Policy information about availability of computer code

Data collection	No software was used in the Data Collection Process.
Data analysis	MS data were analyzed using a combination of Agilent's Lipid Annotator and Profinder applications as well as the web-based MetaboAnalyst 5.0 program.

For manuscripts utilizing custom algorithms or software that are central to the research but not yet described in published literature, software must be made available to editors and reviewers. We strongly encourage code deposition in a community repository (e.g. GitHub). See the Nature Portfolio guidelines for submitting code & software for further information.

Data

Policy information about availability of data

All manuscripts must include a data availability statement. This statement should provide the following information, where applicable:

- Accession codes, unique identifiers, or web links for publicly available datasets
- A description of any restrictions on data availability
- For clinical datasets or third party data, please ensure that the statement adheres to our policy

All data including Western blot images is included in our excel spreadsheet containing the raw data from this study. All lipidomics data used for the Metaboanalyst 5.0 program is publicly available on the Dryad public database (doi:10.5061/dryad.vx0k6djvm). Upon request, the raw mass spectrometry data will be given to those who are interested.

Field-specific reporting

Please select the one below that is the best fit for your research. If you are not sure, read the appropriate sections before making your selection.

☒ Life sciences ☐ Behavioural & social sciences ☐ Ecological, evolutionary & environmental sciences

For a reference copy of the document with all sections, see nature.com/documents/nr-reporting-summary-flat.pdf

Life sciences study design

All studies must disclose on these points even when the disclosure is negative.

Sample size	We used the G*Power application to determine sample sizes that would give us statistical power for 95% confidence with 80% power with an average standard deviation of 25%.
Data exclusions	No data was excluded in this study.
Replication	All experiments were repeated at least twice and yielded the same conclusions.
Randomization	When possible, data and tissue samples were collected from all genotypes and genders of mice in this study. Samples were randomly chosen from each genotype and gender for each experiment.
Blinding	Investigators were only blinded when determining NAFLD severity scores of liver sections.

Reporting for specific materials, systems and methods

We require information from authors about some types of materials, experimental systems and methods used in many studies. Here, indicate whether each material, system or method listed is relevant to your study. If you are not sure if a list item applies to your research, read the appropriate section before selecting a response.

Materials & experimental systems

n/a	Involved in the study	
☐	☒	Antibodies
☐	☒	Eukaryotic cell lines
☒	☐	Palaeontology and archaeology
☐	☒	Animals and other organisms
☒	☐	Human research participants
☒	☐	Clinical data
☒	☐	Dual use research of concern

Methods

n/a	Involved in the study	
☒	☐	ChIP-seq
☒	☐	Flow cytometry
☒	☐	MRI-based neuroimaging

Antibodies

Antibodies used

The following antibodies were used in this study:

Primary Antibodies	Supplier Name	Catalog Number	Lot Number	Application
anti-ACOX1	Proteintech	10957-1-AP	N/A	Western
anti-HSD17B4/DBP	Proteintech	15116-1-AP	00092793	Western
anti-ACAA1	Proteintech	12319-1-AP	N/A	Western
anti-SCP2/SCPx	Proteintech	23006-1-AP	N/A	Western
anti-CAT	Sigma-Aldrich	C0979	059M4838V	Western
anti-CYP4A10	Invitrogen	PA3-033	XG361352	Western
anti-CPT1A	Abcam	ab128568	GR3252377-2	Western
anti-CACT/SLC25A20	Proteintech	19363-1-AP	00059541	Western
anti-CPT2	Proteintech	26555-1-AP	N/A	Western
anti-ECHS1	Abcam	ab153732	GR125011-5	Western
anti-ACSF3	Thermo Fisher	PA525803	VA2920924	Western
anti-MLYCD	Abcam	ab95945	GR187607-35	Western
anti-SLC25A1	Proteintech	15235-1-AP	N/A	Western
anti-PMP70	Abcam	ab3421	GR3386502-1	Western/IHC
anti-PEX14	Proteintech	10594-1-AP	00058233	Western/IHC
anti-APOB	Millipore-Sigma	AB742	2788897	Western
anti-APOA1	Academy Bio-Medical	11A-G2b	012075	Western
anti-PLIN2	Progen	GP40S	802261S	Western
anti-TUB	CST	3873S	16	Western
anti-ACTB	CST	4970S	18	Western

anti-HSC70	Santa Cruz	sc-7298	D2121	Western
anti-GAPDH	EnCor	MCA-1D4	82219	Western
anti-transferrin	Bethyl Laboratories	A80-128A	N/A	Western

Secondary Antibodies	Supplier Name	Catalog Number	Lot Number	Application
Donkey anti-Rabbit IgG 680RD	LI-COR	926-68073	D10113-05	For Rabbit Polyclonal Primary Antibody Westerns
Donkey anti-Rabbit IgG 800CW	LI-COR	926-32213	D01216-10	For Rabbit Polyclonal Primary Antibody Westerns
Donkey anti-Guinea Pig IgG 800CW	LI-COR	925-32411	D10203-01	For PLIN2 Westerns
Donkey anti-Goat IgG 680RD	LI-COR	926-68074	C30826-01	For APOB, APOA, and transferrin Westerns
Goat anti-Mouse IgG1 800CW	LI-COR	926-32350	C90910-25	For TUB and CAT Westerns
Goat anti-Mouse IgG2a 800CW	LI-COR	926-32351	C90904-05	For HSC70 Westerns
Goat anti-Mouse IgM 800CW	LI-COR	925-32280	D00421-03	For GAPDH Western
Donkey anti-Rabbit 488 IgG	Invitrogen	A21206	1981155	For PMP70 and PEX14 IHC
Donkey anti-Rabbit 568 IgG	Invitrogen	A10042	N/A	For PMP70 IHC of murine fibroblasts

Validation

We chose the antibodies used in this study based on either if the antibody was validated by a knockout/knockdown model, manufacturer's website, previous publication, or the antibody database, www.citeab.com. Specifically, we chose to use the following antibodies for these reasons:

anti-ACOX1	Validated using Acox1 KO mouse livers (PMID: 32473093)
anti-HSD17B4/DBP	Validated using HSD17B4 knockdown cells (manufacturer's website)
anti-ACAA1	Routinely used in publications evaluating peroxisome proteins
anti-SCP2/SCPx	Validated by SCP2/SCPx knockdown (manufacturer's website)
anti-CAT	Validated using Pex5 KO Schwann Cells that shows diffuse catalase staining (PMID: 28470148)
anti-CYP4A10	Used with mouse liver samples (PMID:33450224)
anti-CPT1A	Validated using Cpt1a KO HAP1 cells (manufacturer's website)
anti-CACT/SLC25A20	Used with Mouse liver samples (PMID: 29116185)
anti-CPT2	Used with Mouse liver samples (PMID: 34564857)
anti-ECHS1	Validated using Echs1 Deficient Human Fibroblasts (PMID: 26251176)
anti-ACSF3	Validated using Acsf3 KO Human Fibroblasts (PMID: 28479296)
anti-MLYCD	Used with Mouse liver samples (PMID: 30201971)
anti-SLC25A1	Validated using Slc25a1 knockdown mouse NIH/3T3 mouse fibroblast cells (PMID: 32134147)
anti-PMP70	Routinely used to detect PMP70 on Western blots and tissue sections (manufacturer's website)
anti-PEX14	Routinely used to detect PEX14 on Western blots and tissue sections (manufacturer's website)
anti-APOB	Used previously with mouse plasmas (PMID: 30808757)
anti-APOA1	Used previously with mouse plasmas (PMID: 30808757)
anti-PLIN2	Used with Mouse MLTC-1 cells (PMID: 27554864)
anti-TUB	Routinely used as loading control (www.citeab.com)
anti-ACTB	Routinely used as loading control (www.citeab.com)
anti-HSC70	Routinely used as loading control (www.citeab.com)
anti-GAPDH	Routinely used as loading control (www.citeab.com)
anti-transferrin	Routinely used as loading control (www.citeab.com)

Eukaryotic cell lines

Policy information about cell lines

Cell line source(s)

Mouse fibroblasts were cultured from WT, Tmem135 TG, and Tmem135 mutant (FUN025) mouse ears as described in the Materials and Methods Section.

Authentication

The cultures were not authenticated with staining for fibroblast markers.

Mycoplasma contamination

All cell lines tested negative for Mycoplasma contamination.

Commonly misidentified lines
(See ICLAC register)

N/A

Animals and other organisms

Policy information about studies involving animals; ARRIVE guidelines recommended for reporting animal research

Laboratory animals

B6.Cg-Lepob/J (Lepob) (Stock #000632) and B6;129S4-Pparatm1Gonz (Ppara ko) (Stock #008154) were obtained from The Jackson Laboratory (Bar Harbor, ME) and bred in the animal facility at the University of Wisconsin-Madison. Tg(CAG-Tmem135)#Aike (Tmem135 TG) mice congenic on the C57BL/6J background and mutant Tmem135 (Tmem135 FUN025/FUN025) mice were generated as previously described (PMID: 27863209, 30102730, 33064130). C57BL/6J mice served as WT controls for this study. All mice were fed a global soy protein-free extruded rodent diet (#2020X, Envigo, Madison, WI) and housed in the Medical Sciences Center Vivarium at the University of Wisconsin-Madison. Both males and females were used in this study. All numbers of mice used

	in experiments are provided within the figures and their legends.
Wild animals	No wild animals were used in this study.
Field-collected samples	No field-collected samples were used in this study.
Ethics oversight	All mouse procedures were performed in accordance with the protocols approved by the Animal Care and Use Committee at the University of Wisconsin-Madison.

Note that full information on the approval of the study protocol must also be provided in the manuscript.
